# Genetic knockdown of DYRK1A attenuates cognitive impairment, Aβ pathology, tauopathy and neuroinflammatory responses in mouse models of AD

**DOI:** 10.3389/fimmu.2025.1661791

**Published:** 2025-11-11

**Authors:** Hyun-ju Lee, Sora Kang, Yoo Jin Lee, Seokjun Oh, Bitna Joo, Jeong-Woo Hwang, Jeongseop Kim, Tae-Eun Kim, Tae-Mi Jung, Yu-Jin Kim, Ji-Yeong Jang, Jeong-Heon Song, Ja Wook Koo, Hyang-Sook Hoe

**Affiliations:** 1 Department of Neural Development and Disease, Korea Brain Research Institute (KBRI), Daegu, Republic of Korea; 2 AI-based Neurodevelopmental Diseases Digital Therapeutics Group, Korea Brain Research Institute (KBRI), Daegu, Republic of Korea; 3 Department of Brain Sciences, Daegu Gyeongbuk Institute of Science & Technology, Daegu, Republic of Korea; 4 Department of Pharmacology, School of Medicine, Daegu Catholic University, Daegu, Republic of Korea

**Keywords:** DYRK1A, neuroinflammation, amyloid beta, tauopathy, cognitive function, Alzheimer’s disease

## Abstract

**Introduction:**

Dual specificity tyrosine phosphorylation-regulated kinase 1A (DYRK1A) is associated with the pathoprogression of neurodevelopmental and neurodegenerative disorders. However, the effects of direct genetic manipulation of DYRK1A in the brain on cognitive function, neuroinflammation and Alzheimer’s disease (AD) pathology and underlying molecular mechanisms have not been fully investigated.

**Methods:**

To determine whether overexpressing or knocking down DYRK1A expression directly in the brain affects cognitive function, neuroinflammation and AD pathology, adeno-associated viruses (AAVs) were injected into the hippocampus of wild-type (WT), 5xFAD, and PS19 mice. Then, cognitive function was assessed via Y-maze and novel object recognition (NOR) tests, and neuroinflammatory responses and AD pathologies were analyzed by real-time PCR, Western blotting, immunofluorescence staining, AD-associated protein activity assays and ELISA.

**Results and discussion:**

In WT mice, hippocampal DYRK1A overexpression significantly reduced short-term spatial/recognition memory and SynGAP expression while increasing p-P38 levels. Conversely, in amyloid-beta (Aβ)-overexpressing 5xFAD mice, hippocampal DYRK1A knockdown improved short-term spatial/recognition memory and significantly increased CaMKIIα and CREB phosphorylation. Moreover, hippocampal DYRK1A knockdown in 5xFAD mice significantly suppressed mRNA levels of proinflammatory cytokines and markers of AD-associated reactive astrocytes (RAs), disease-associated microglia (DAMs), and RA–DAM interactions. However, hippocampal DYRK1A overexpression in 5xFAD mice increased mRNA levels of the proinflammatory cytokine IL-1β, RA markers and the microglial marker Iba-1. Interestingly, hippocampal DYRK1A knockdown in 5xFAD mice significantly increased levels of the anti-oxidative/inflammatory molecule HO-1 without altering p-STAT3/p-NF-κB levels. By contrast, hippocampal DYRK1A overexpression in 5xFAD mice enhanced STAT3/NF-κB phosphorylation but did not affect ROS levels. Importantly, hippocampal DYRK1A knockdown in 5xFAD mice significantly reduced Aβ plaque number, soluble Aβ40 levels, and soluble/insoluble Aβ42 levels by suppressing β-secretase BACE1 activity but not tau hyperphosphorylation. Finally, hippocampal DYRK1A knockdown in PS19 mice [a model of AD that overexpresses human mutant tau (P301S)] selectively decreased insoluble tau hyperphosphorylation at Ser396 and Ser404 and alleviated proinflammatory responses/glial-associated neuroinflammatory dynamics. Taken together, our data indicate that DYRK1A modulates cognitive function, neuroinflammation, and AD pathology (Aβ and tauopathy) in mouse models of AD and/or WT mice and support DYRK1A as a potential therapeutic target for AD.

## Introduction

Alzheimer’s disease (AD) is a progressive neurodegenerative disease characterized by cognitive impairment and behavioral disturbances. A key neuropathological hallmark of AD is the extracellular accumulation of amyloid-beta (Aβ) plaques (1, 2). Previous studies reported that soluble oligomers of Aβ, which is formed by the sequential proteolytic cleavage of amyloid precursor protein (APP) by β- and γ-secretases (3), are responsible for the disruptions of synaptic communication, the induction of glial hyperactivation, and the subsequent neuroinflammation that ultimately lead to neuronal degeneration and cognitive decline (4). Another neuropathological hallmark of AD is the intracellular formation of neurofibrillary tangles (NFTs), which are composed of hyperphosphorylated tau protein. In healthy neurons, tau stabilizes microtubules and plays a critical role in axonal transport and neuronal function (5). However, when hyperphosphorylated, tau loses its ability to bind microtubules, leading to microtubule destabilization, impaired cellular transport, and consequently contributed to neuronal dysfunction and degeneration. NFTs are associated with neuronal dysfunction and death, memory loss, neuroinflammatory dynamics, and the progression of AD pathogenesis (6). Therefore, elucidating the underlying mechanisms for the regulation of Aβ accumulation and tau pathology is crucial for developing effective therapeutic strategies for AD.

Dual specificity tyrosine phosphorylation-regulated kinase 1A (DYRK1A) plays a crucial role in physiological and pathological processes in the brain. Several studies reported that DYRK1A is involved in essential neuronal functions such as neurogenesis, neuronal differentiation, and dendritic spine formation and maturation, as well as in fundamental cellular processes including cell growth and division (7–10). In addition, DYRK1A is located on human chromosome 21, and its overexpression has been implicated in multiple diseases, most notably Down syndrome and AD (11–13). Specifically, DYRK1A resides in the Down syndrome critical region (DSCR) and contributes to various phenotypes of Down syndrome, including cognitive disability and memory and learning impairments (14–19). Importantly, genetic overexpression of DYRK1A leads to APP phosphorylation at Thr688, which enhances the binding affinity of APP to β-/γ-secretases, resulting in Aβ accumulation (10). DYRK1A also directly phosphorylates tau, a key step required for the formation of NFT (20). We and others previously reported that small-molecule inhibitors of DYRK1A (e.g., KVN93 and Dyrk1-inh) alleviate LPS-induced neuroinflammation by modulating TLR4/AKT/STAT3 and TLR4/NF-κB signaling pathways and reduce AD-associated microglial/astroglial activation (21, 22). Pharmacological inhibition of DYRK1A also significantly decrease Aβ pathology in 5xFAD and 3xTg mice (22, 23). Collectively, previous findings suggest that DYRK1A could be a major regulator of AD pathology.

However, the precise molecular mechanisms underlying the direct effects of DYRK1A inhibition in the brain have not been fully elucidated. It is possible that pharmacological DYRK1A inhibitors modulate AD pathology through its off-target (e.g., MAO-A and CK1) which are also in volved in AD pathogenesis (24, 25). To separate the direct effects of DYRK1A inhibition from these off-target effects, in the present study, we examined the effects of direct modulation of DYRK1A expression in the brain on cognitive function and AD pathology as well as the underlying molecular mechanisms. An adeno-associated virus (AAV) vector was used to knock down or overexpress DYRK1A in the hippocampus of wild-type (WT) mice, 5xFAD mice (Aβ-overexpressing AD mouse model), and PS19 mice (tau-overexpressing AD mouse model). We found that hippocampal DYRK1A overexpression in WT mice significantly impaired short-term and long-term memory, along with reducing SynGAP levels and increasing P38 phosphorylation. However, knocking down DYRK1A in the hippocampus in 5xFAD mice improved short-term spatial/recognition memory and increased p-CaMKIIα/p-CREB levels. In addition, hippocampal DYRK1A knockdown in 5xFAD mice significantly downregulated mRNA levels of proinflammatory cytokines and markers of AD-related neuroinflammatory dynamics. Conversely, overexpressing DYRK1A in the hippocampus in 5xFAD mice selectively exacerbated AD-evoked neuroinflammatory mediators [IL-1β, RA (reactive astrocyte) markers, IBA-1, CR3]. Moreover, in 5xFAD mice, hippocampal DYRK1A knockdown increased levels of the anti-oxidative/inflammatory molecule HO-1 but not neuroinflammation-associated downstream STAT3/NF-κB signaling, whereas hippocampal DYRK1A overexpression significantly enhanced STAT3/NF-κB phosphorylation without altering ROS levels. More importantly, hippocampal DYRK1A knockdown significantly alleviated Aβ pathology (e.g., senile plaque accumulation and soluble/insoluble Aβ levels) by inhibiting BACE-1 activity in 5xFAD mice. Finally, hippocampal DYRK1A knockdown in PS19 mice selectively decreased insoluble p-Tau^Ser396^ and p-Tau^Ser404^ and suppressed tau-mediated neuroinflammatory responses/AD-related glial dynamics. Collectively, these results indicate that direct genetic DYRK1A modulation (knockdown or overexpression) in the brain modulates memory performance and various AD-related pathologies including proinflammatory responses, Aβ burden, and tauopathy in 5xFAD, PS19, and/or WT mice implicating DYRK1A as a promising target for AD intervention.

## Materials and methods

### 5xFAD, PS19, and wild-type mice

3.5- and 6-month-old male 5xFAD mice (B6Cg-Tg APPSwFlLon, PSEN1*M146L*L286V6799Vas/Mmjax; stock # 34848-JAX) and 4-month-old male human P301S tau transgenic mice (PS19 mice) (B6;C3-Tg (Prnp-MAPT*P301S)PS19Vle/J, Stock No. 008169) were purchased from Jackson Laboratory (Bar Harbor, ME, USA), and 3- and 3.5-month-old male C57BL6/N (WT) mice were purchased from Orient-Bio Company (Gyeonggi-do, Korea). All animals were housed in a pathogen-free facility with a photoperiod of 12 h and environmental control at 22°C. Food and water were freely accessible to the mice throughout the experiment.

### AAV-hSyn-mDYRK1A-EGFP

#### Cells

AAVpro^®^ 293T cells (cat. no. 632273, Clontech, Mountain View, CA, USA) were cultured in Dulbecco’s modified Eagle’s Medium (DMEM; cat. no. 11965092, Gibco, Grand Island, NY, USA) with 5% fetal bovine serum (FBS; cat. no. 16000-044, Invitrogen, Carlsbad, CA, USA) and penicillin-streptomycin solution (cat. no. 15140122, Gibco). The cells were maintained at 37°C in an atmosphere of humidified air containing 5% CO_2_.

#### Plasmids

The AAV plasmid backbone was based on pAAV-hSyn-EGFP (cat. no. 50465, Addgene, Watertown, MA, USA). The full-length *DYRK1A* gene was amplified from total RNA from mouse hippocampal tissue by real-time PCR with the primers pAAV-hSyn-DYRK1A-EGFP-F (5’-AGAAGGTACCGGATCCGTCGACGCCACCATGCatacag-3’, BamH1 restriction sequence underlined) and pAAV-hSyn-Dyrk1a-EGFP-R (5’- CCATGGTGGCGGATCCGTCGACTGCCGAGCTAGCTACA-3’, BamH1 restriction sequence underlined). Total RNA from mouse hippocampus tissue was isolated using an RNeasy mini kit (cat. no. 74106, Qiagen, Venlo, Netherlands), and real-time PCR was performed using the PrimeScript™ 1^st^ strand cDNA Synthesis Kit (cat. no. 6110A, Takara, Shiga, Japan). The amplified DYRK1A cDNA (~2.5 kb) was inserted into pAAV-hSyn-EGFP using the BamH1 restriction site. pAAV-RC and pHelper plasmids were purchased from Agilent (cat. no. 240071, Santa Clara, CA, USA).

#### Virus production and purification

AAVpro 293T cells were co-transfected with the recombinant pAAV expression plasmid (pAAV-RC) and pHelper using polyethylenimine (PEI; cat. no. 24765, Polyscience, Addgene). At least 72 h after transfection, AAV particles from the cell medium were harvested and purified as described in Addgene’s protocols (https://www.addgene.org/protocols/#virus, last accessed Sep. 29, 2020).

### AAV-U6-mDYRK1A shRNA-EGFP

To investigate the effects of DYRK1A knockdown on cognitive function, amyloidopathy, tau hyperphosphorylation, and neuroinflammation, AAV-U6-control shRNA-EGFP or AAV-U6-mDYRK1A shRNA-EGFP (cat. no. shAAV-257590, Vector Biolabs, Malvern, PA, USA) was injected into the mouse brain.

### Stereotaxic viral injection

All injections were conducted under intraperitoneally administered anesthesia with ketamine (100 mg/kg) and xylazine (10 mg/kg) in 0.1 M phosphate-buffered saline (PBS). The virus was injected into the bilateral hippocampus (bregma: -2.0 mm AP, ± 1.5 mm ML, and -1.55 mm DV) in a volume of 0.5 to 1.0 μL in each hemisphere at a rate of 0.1 μL/min using a 5-μL syringe (cat. no. 7641–01, Hamilton, Reno, Nevada, USA) with a 33-gauge needle (cat. no. 7762–06, Hamilton, Reno, Nevada, USA). After injection, the needle was left in place for at least 10 min to allow diffusion of the virus at the injection site. The mice were then allowed to recover for 3–4 weeks before further behavioral experiments.

### Behavioral testing paradigm

#### Y-maze test

The Y-maze test was performed to measure short-term spatial memory. A single mouse injected with AAV-control, AAV-DYRK1A, AAV-control shRNA, or AAV-DYRK1A shRNA was placed in one of the three arms (35 cm x 7 cm x 15 cm) of the maze, which met at an angle of 120°, and allowed to explore freely for 5 min. Spontaneous alternations were recorded and analyzed using SMART video tracking software (Panlab, Barcelona, Spain). The alternation percentage was calculated by dividing the number of alternations by the number of alternation triads.

#### Novel object recognition test

To evaluate recognition memory, the NOR test was performed as previously described with minor modifications (24, 25). Briefly, each mouse underwent a 5-min training phase in an open-field box (40 cm x 40 cm x 25 cm) containing two identical objects. Between trials, odor cues were eliminated by thoroughly swabbing the apparatus and objects with 70% ethanol. Twenty-four hours later, the mouse was returned to the same apparatus containing one familiar object and one novel object for a 5-min retention testing phase. The locations of the two objects in the apparatus were counterbalanced. The trials were recorded, and the recordings were used to manually count the time of exploratory behavior, defined as pointing of the mouse’s nose toward an object. Object preference (%) was calculated using the formula [Preference (%) of object = T _Novel_/(T _Familiar_ + T _Novel_) × 100], where T_novel_ is the time of exploration of the novel object and T_Familiar_ is the time of exploration of the familiar object.

### Real-time PCR

To analyze the effect of genetic DYRK1A modulation on DYRK1A and neuroinflammation-associated markers mRNA levels, RNA was extracted from hippocampal tissue of WT, 5xFAD, and/or PS19 mice using TRIzol (Invitrogen, Waltham, MA, USA) (25). The extracted RNA was used with the Superscript cDNA Premix Kit II (cat. no. SR-5000, GeNetBio, Daejeon, Republic of Korea) to synthesize cDNA for use in real-time PCR. Forty-cycle real-time PCR was performed in a QuantStudio™ 5 system (Thermo Fisher Scientific, Waltham, MA, USA) with Fast SYBR Green Master Mix (Thermo Fisher Scientific, Waltham, MA, USA). Normalization was performed using the cycle threshold (Ct) value for *gapdh*. The primer sequences are provided in **Supplementary Table 1**.

### Immunofluorescence staining

To assess whether DYRK1A overexpression or knockdown in brain directly affects DYRK1A protein expression and Aβ plaque accumulation in WT or 5xFAD mice, immunofluorescence staining was performed. For this experiment, mouse brain sections were first rinsed in PBST (PBS containing 0.2% Triton-X 100). Next, the brain sections were incubated in blocking solution [10% normal goat serum (cat. no. S-1000-20, Vector Laboratories, Burlingame, CA) in PBST] for 2 h at room temperature (RT). The primary antibodies were added, and the brain sections were incubated for 24–72 h at 4°C. After washing with PBST three times, the brain sections were incubated for 2 h at RT with Alexa Fluor 555-conjugated goat anti-rabbit or anti-mouse secondary antibodies. The brain sections were then washed with PBST, PBST/DAPI, and PBS before being mounted on glass slides with a mounting solution containing DAPI (cat. no. H-1200-10, Vector Laboratories). The immunostained tissue was imaged by fluorescence microscopy (DMi8, Leica Microsystems), and immunofluorescence staining was quantified using ImageJ software (http://imagej.net/ij, Version 1.53e, US National Institutes of Health, Bethesda, MD, USA, last accessed April 27, 2025). Detailed antibody information is provided in **Supplementary Table 2**.

### Western blotting

To determine the effects of DYRK1A gene manipulation on memory-regulating protein levels, DYRK1A expression, inflammation-associated molecule levels, and tau hyperphosphorylation in WT, 5xFAD, and/or PS19 mice, the mouse hippocampus was homogenized in RIPA lysis buffer (Merck Millipore, Billerica, MA, USA) containing 1% protease and phosphatase inhibitor cocktail (Thermo Scientific, Waltham, MA, USA) for 1 h on ice. The lysate was then centrifuged three times for 20 min at 20,000 × g and 4°C, and the supernatant was collected and stored at −20°C until analysis.

To assess the effect of DYRK1A knockdown on cognitive function and tauopathy in PS19 mice, the entorhinal cortex and hippocampus were dissected and homogenized in RIPA lysis buffer supplemented with a protease and phosphatase inhibitor cocktail (Thermo Scientific). The homogenates were incubated at 4°C for 1 h and centrifuged at 20,000 × g at 4°C for 20 min. The supernatant was collected as the RIPA-soluble fraction and stored at −80°C until analysis. The pellet was washed once with 1 M sucrose in RIPA lysis buffer, resuspended in 2% SDS solution, and incubated at RT for 1 h. The suspension was sonicated and centrifuged at 20,000 × g for 1 min at RT, and the supernatant was collected as the RIPA-insoluble fraction and stored at −80°C until analysis.

To separate proteins by electrophoresis, 10 μg of protein was heated for 10 min at 100°C and loaded onto an SDS-polyacrylamide gel. The separated proteins were then electrotransferred to a PVDF membrane (Millipore, Billerica, MA, USA), After blocking with 5% skim milk at RT for 1 h, the membrane was incubated with anti-DYRK1A, anti-SynGAP, anti-p-P38, anti-P38, anti-p-CaMKIIα, anti-CaMKIIα, anti-p-CREB, anti-CREB, anti-PLK2, anti-HO-1, anti-p-AKT, anti-AKT, anti-p-STAT3, anti-STAT3, anti-p-NF-κB, anti-NF-κB, anti-NR2A, anti-NR2B, anti-GluA1, anti-GluA2, anti-EAAT1, anti-EAAT2, anti-p-ERK, anti-ERK, anti-PS-1-CTF, anti-p-APP^Thr668^, anti-p-Tau^Ser202/Thr205^ (AT8), anti-p-Tau^Thr212/Ser214^ (AT100), anti-p-Tau^Thr231^ (AT180), anti-p-Tau^Ser396^, anti-p-Tau^Ser404^, p-GSK3α/β, anti-p-CDK5, anti-GAPDH or anti-β-actin antibodies overnight at 4°C. The following day, the membrane was incubated with HRP-conjugated goat anti-rabbit IgG or HRP-conjugated goat anti-mouse IgG for 1 h, and detection was realized with ECL Western Blotting Detection Reagent (GE Healthcare, Chicago, IL, USA). Images were acquired and analyzed by Fusion Capt Advance software (Vilber Lourmat, Collégien, France). Detailed antibody information is provided in **Supplementary Table 3**.

### Enzyme-linked immunosorbent assay

#### RIPA-soluble Aβ40 ELISA in 3.5-month-old 5xFAD mice

To investigate whether direct inhibition of DYRK1A gene expression alters Aβ pathology in the brains of younger AD model mice, hippocampal Aβ40 levels were measured by ELISA. Hippocampal tissue from 3.5-month-old 5xFAD mice injected with AAV-control shRNA or AAV-DYRK1A shRNA was homogenized in RIPA lysis buffer (Merck Millipore, Billerica, MA, USA) containing 1% protease and phosphatase inhibitor cocktail (Thermo Scientific, Waltham, MA, USA) for 1 h on ice. The lysates were then centrifuged three times for 20 min at 20,000 × g and 4°C, and the supernatant (RIPA-soluble fraction) was collected for analysis.

Aβ40 levels were analyzed by using the Human Amyloid beta 40 ELISA Kit (cat. no. KHB3481, Invitrogen, Carlsbad, CA, USA) according to the manufacturer’s instructions. Briefly, serially diluted Human Aβ40 standards (500 pg/ml to 0 pg/ml, 50 μl/well) or RIPA-soluble fraction (50 μl/well) were loaded into the pre-coated 96-well plate followed by human Aβ40 detection antibody (50 μl/well) and incubated for 3 h at RT. Next, the plate was washed with 1× wash buffer four times, and anti-rabbit IgG HRP (100 μl/well) was added and incubated for 1 h at RT. Then, the plate was washed with 1× wash buffer six times, and stabilized chromogen (tetramethylbenzidine) was added and incubated for 30 min. Finally, stop solution was added, and optical density was measured at 450 nm.

#### DEA-soluble and DEA-insoluble Aβ40 and Aβ 42 ELISA in 6-month-old 5xFAD mice

To assess the effect of DYRK1A knockdown on Aβ pathology in aged AD mice, soluble and insoluble Aβ40 and Aβ42 levels were measured by ELISA. For this experiment, 6-month-old 5xFAD mice were injected with AAV-control shRNA or AAV-DYRK1A shRNA, and hippocampal tissue was dissected and homogenized in tissue homogenization buffer (250 mM sucrose, 20 mM Tris-HCl, 1 mM EDTA, 1 mM EGTA). The tissue homogenate was then added to 0.4% diethylamino (DEA) solution containing 1% protease and phosphatase inhibitor cocktail (Thermo Scientific, Waltham, MA, USA), sonicated, and ultracentrifuged at 47,000 rpm for 1 h at 4°C. The supernatant was collected, neutralized with Tris-HCL buffer (pH 6.8), and stored at –80°C until analysis of DEA-soluble Aβ levels. The remaining pellet was resuspended in formic acid and ultracentrifuged at 47,000 rpm for 1 h at 4°C, and the supernatant was collected, neutralized with Tris-HCl buffer (pH 8.8), and stored at –80°C until analysis of DEA-insoluble Aβ levels.

DEA-soluble/DEA-insoluble Aβ40 and Aβ42 levels were analyzed using the Human Amyloid beta 40 ELISA Kit (cat. no. KHB3481, Invitrogen, Carlsbad, CA, USA) and the Human Amyloid beta 42 ELISA Kit (cat. no. KHB3441, Invitrogen, Carlsbad, CA, USA), respectively, according to the manufacturer’s instructions. Briefly, to detect human Aβ40, serially diluted Human Aβ40 standards (500 pg/ml to 0 pg/ml, 50 μl/well) and the DEA-soluble or DEA-insoluble fraction (50 μl/well) were loaded into the pre-coated 96-well plate followed by human Aβ40 detection antibody (50 μl/well) and incubated for 3 h at RT. To detect human Aβ42, serially diluted Human Aβ42 standards (500 pg/ml to 0 pg/ml, 50 μl/well) and the DEA-soluble or DEA-insoluble fraction (50 μl/well) were loaded into the pre-coated 96-well plate followed by human Aβ42 detection antibody (50 μl/well) and incubated for 3 h at RT. Next, the plate was washed with 1× wash buffer four times, and anti-rabbit IgG HRP (100 μl/well) was added and incubated for 1h at RT. Then, the plate was washed with 1× wash buffer six times, and stabilized chromogen (tetramethylbenzidine) was added and incubated for 30 min. Finally, stop solution was added, and the optical density was measured at 450 nm.

#### Proinflammatory cytokine ELISA in 3.5-month-old 5xFAD mice

To determine whether genetic DYRK1A knockdown alters proinflammatory responses at the protein level, 3.5-month-old 5xFAD mice were injected with AAV-control shRNA or AAV-DYRK1A shRNA in the hippocampus. Three weeks after the injection, the hippocampal tissue was dissected and homogenized in RIPA lysis buffer (Merck Millipore, Billerica, MA, USA) containing 1% protease and phosphatase inhibitor cocktail (Thermo Scientific, Waltham, MA, USA) for 1 h on ice. The lysates were then centrifuged three times for 20 min at 20,000 × g and 4°C, and the supernatant (RIPA-soluble fraction) was collected and used to determine the protein concentration. COX-2, IL-1β, IL-6, and TNF-α protein levels were measured using a COX-2 ELISA kit (DYC4198-5, R&D Systems, Minneapolis, MN, USA) and IL-1β, IL-6, and TNF-α ELISA kit (88-7013A-88 for IL-1β, 88-7064–22 for IL-6, 88-7324–22 for TNF-α, Invitrogen, Waltham, Massachusetts, USA) according to the manufacturer’s instructions.

### ROS assessment

To investigate the effect of genetic knockdown of DYRK1A on oxidative stress in 5xFAD mice, 3.5-month-old 5xFAD mice were injected with AAV-control shRNA or AAV-DYRK1A shRNA in the hippocampus. In addition, to test whether overexpression of the DYRK1A gene affects oxidative stress in 5xFAD mice, 3.5-month-old 5xFAD mice were injected with AAV-control or AAV-DYRK1A in the hippocampus. Three weeks after the injection, the hippocampal tissue was dissected and homogenized in RIPA lysis buffer (Merck Millipore, Billerica, MA, USA) containing 1% protease and phosphatase inhibitor cocktail (Thermo Scientific, Waltham, MA, USA) for 1 h on ice. The lysates were then centrifuged three times for 20 min at 20,000 × g and 4°C, and the supernatant (RIPA-soluble fraction) was collected. ROS levels were measured using 2’,7’-dichlorofluorescein diacetate (DCFH-DA, cat. no. 287810, Sigma–Aldrich, Burlington, MA, USA). Briefly, the RIPA-soluble fraction (50 μl/well) was added to a 96-well plate, and 500 μM DCFH-DA solution (50 μl/well) was added. After incubating the plate for 1.5 h at 37°C, fluorescence intensity was measured at Ex/Em=488 nm/522 nm.

### Activity test

#### ADAM17 activity

To examine the underlying molecular mechanisms for the effect of DYRK1A knockdown on Aβ plaque deposition and Aβ levels in 5xFAD mice, the activity of ADAM17, an α-secretase involved in non-amyloidogenic APP proteolytic processing, was measured. For this experiment, hippocampal tissues of AAV-control shRNA-injected or AAV-DYRK1A shRNA-injected 5xFAD mice were homogenized in RIPA lysis buffer (Merck Millipore, Billerica, MA, USA) containing 1% protease and phosphatase inhibitor cocktail (Thermo Scientific, Waltham, MA, USA) for 1 h on ice. The lysates were then centrifuged three times for 20 min at 20,000 × g and 4°C, and the supernatant was collected for analysis. ADAM17 activity was assessed by using the SensoLyte^®^ 520 ADAM17 Activity Assay Kit (cat. no. AS-72085, AnaSpec, Fremont, CA, USA) according to the manufacturer’s instructions. Briefly, hippocampal homogenate and ADAM17-specific fluorogenic substrate were loaded into a 96-well plate and incubated for 3 h, stop solution was added, and the fluorescence intensity was measured at Ex/Em=490 nm/520 nm.

#### BACE-1 activity

To elucidate underlying mechanisms by which DYRK1A suppression ameliorates Aβ pathology in 5xFAD mice, the activity of BACE-1, a β-secretase involved in amyloidogenic processing of APP, was analyzed. To assess this, hippocampal lysates of 5xFAD mice injected with AAV-control shRNA or AAV-DYRK1A shRNA were prepared as described in *ADAM17 activity*. BACE-1 activity was assessed by using the SensoLyte^®^ 520 ß-Secretase (BACE1) Activity Assay Kit (cat. no. AS-71144, AnaSpec, Fremont, CA, USA) according to the manufacturer’s instructions. Briefly, hippocampal homogenate and β-secretase-specific fluorogenic substrate were loaded into a 96-well plate and incubated for 3 h. Next, stop solution was added, and the fluorescence intensity was measured at Ex/Em=490 nm/520 nm.

#### IDE activity

The activity of insulin-degrading enzyme (IDE), an Aβ-degrading enzyme, was measured to determine whether DYRK1A inhibition decreases Aβ pathology via IDE activity in 5xFAD mice. For this experiment, hippocampal lysates of 5xFAD mice injected with AAV-control shRNA or AAV-DYRK1A shRNA were prepared as described in *ADAM17 activity*. IDE activity was assessed by using the SensoLyte^®^ 520 IDE Activity Assay Kit (cat. no. AS-72231, AnaSpec, Fremont, CA, USA) according to the manufacturer’s instructions. Briefly, hippocampal homogenate and IDE-specific fluorogenic substrate were loaded into a 96-well plate and incubated for 3 h. Then, stop solution was added, and fluorescence intensity was measured at Ex/Em=490 nm/520 nm.

#### NEP activity

To analyze the specific molecular mechanisms by which DYRK1A knockdown mitigates Aβ pathology in 5xFAD mice, the activity of neprilysin (NEP), an Aβ-degrading enzyme, was measured. To assess this, hippocampal lysates from 5xFAD mice treated with AAV-control shRNA or AAV-DYRK1A shRNA were prepared as described in *ADAM17 activity*. NEP activity was quantified by using the SensoLyte^®^ 520 NEP Activity Assay Kit (cat. no. AS-72223, AnaSpec, Fremont, CA, USA) according to the manufacturer’s instructions. Briefly, hippocampal homogenate and NEP-specific fluorogenic substrate were loaded into a 96-well plate and incubated for 3h. Then, stop solution was added, and fluorescence intensity was measured at Ex/Em=490 nm/520 nm.

### Statistical analysis

All data were analyzed using a two-tailed unpaired *t*-test in GraphPad Prism 10 (GraphPad Software, San Diego, CA. USA). Data are presented as the mean ± S.E.M. (*p < 0.05, **p < 0.01, ***p < 0.001, ****p < 0.0001). Detailed statistical analysis results are provided in **Supplementary Table 4**.

## Results

### DYRK1A overexpression decreases short-term spatial/recognition memory, suppresses SynGAP expression, and increases p-P38 levels in WT mice

**Figure 1:**
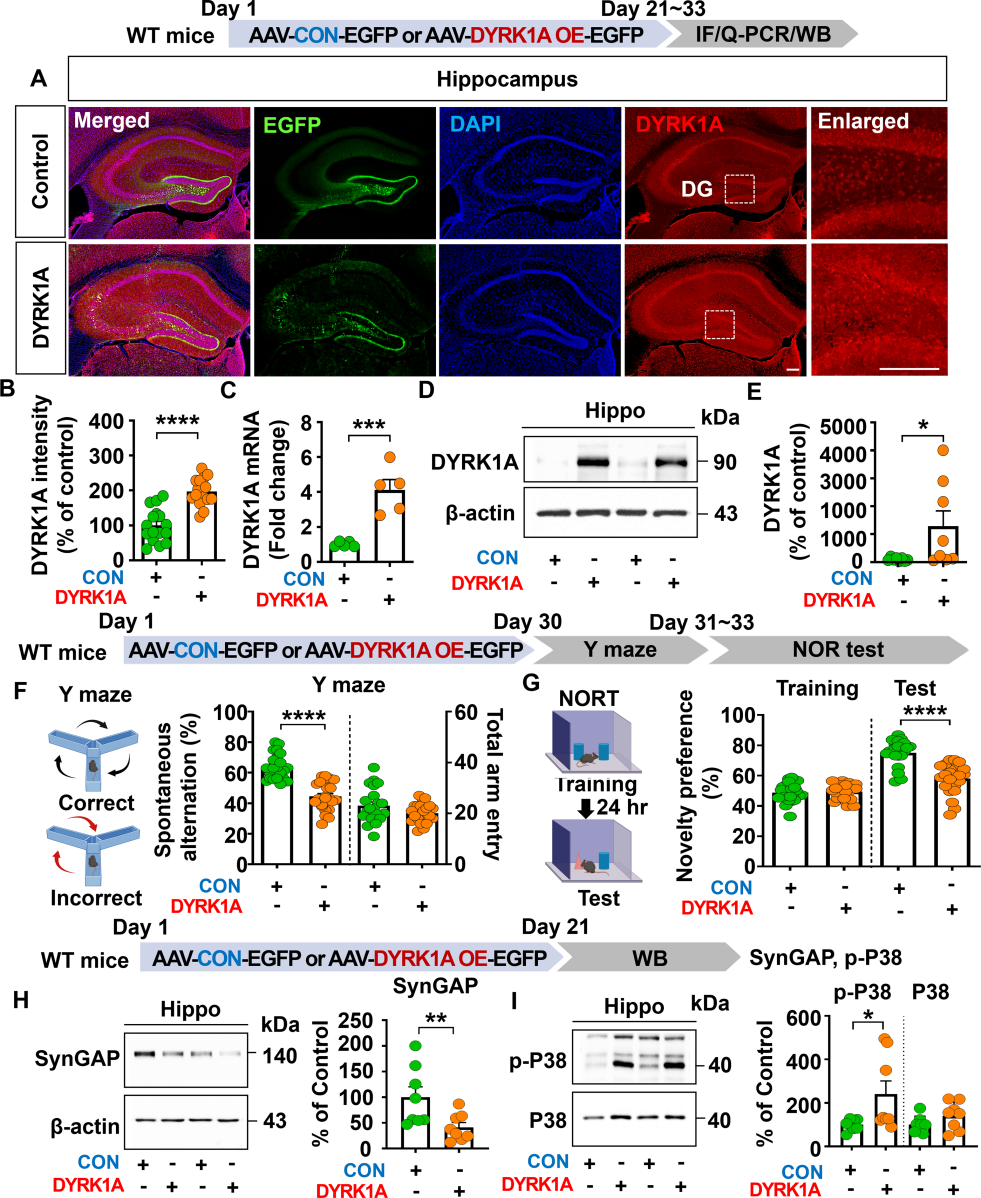
DYRK1A overexpression impairs cognitive function, reduces SynGAP expression, and increases P38 signaling in WT mice. **(A, B)** DYRK1A immunofluorescence in hippocampal slices from WT mice injected with AAV-Control or AAV-DYRK1A (n = 16 brain slices from 4 mice/group). **(C)** Real-time PCR analysis of hippocampal DYRK1A mRNA levels in WT mice treated as describe above (n = 5 mice/group). **(D, E)** Western blotting of hippocampal DYRK1A levels in WT mice treated as describe above (n = 8 mice/group). **(F, G)** Results of Y-maze and NOR tests of WT mice 30 days after treatment as describe above (n= 22–23 mice/group). **(H, I)** Western blotting analysis of hippocampal lysates from WT mice treated as describe above with anti-SynGAP, anti-p-P38, anti-P38 and anti-β-actin antibodies (n = 8 mice/group). *p < 0.05, **p < 0.01, ***p < 0.001, ****p < 0.0001. Scale bar = 200 µm.

To investigate the effects of DYRK1A overexpression on cognitive function *in vivo*, 3-month-old WT mice were injected with AAV-control or AAV-DYRK1A in the hippocampus. Three weeks after injection, immunofluorescence staining of hippocampal tissue with an anti-DYRK1A antibody showed that DYRK1A fluorescence intensity was significantly increased in AAV-DYRK1A-injected WT mice than in AAV-control-injected WT mice (**Figures 1A, B**).

Consistently, real-time PCR analysis revealed that hippocampal DYRK1A mRNA expression was markedly enhanced by 408.63% in AAV-DYRK1A–injected WT mice compared to AAV–control-injected WT mice, confirming successful overexpression of DYRK1A (**Figure 1C**). Further confirming these findings, western blotting showed that hippocampal DYRK1A expression was significantly upregulated in AAV-DYRK1A-injected WT mice than AAV-control injection (**Figures 1D, E**). In behavioral assessments, AAV-DYRK1A-injected WT mice exhibited significantly reduced spontaneous alterations in the Y-maze test and novelty preference in the novel object recognition (NOR) test compared to AAV-control-injected WT mice (**Figures 1F, G**).

To investigate the molecular mechanisms by which DYRK1A overexpression impairs learning and memory *in vivo*, WT mice were injected as described above, and the hippocampus was dissected. To assess the expression of Ras signaling-associated molecules, western blotting was performed with anti-p-CaMKIIα/CaMKIIα, anti-p-CREB/CREB, and anti-p-ERK/ERK antibodies, and found that the phosphorylation of CaMKIIα, CREB, and ERK did not altered in AAV-DYRK1A-injected compared to AAV-control-injected WT mice (**Supplementary Figures 1A–C**). To assess other memory-regulating molecules, western blotting was conducted with antibodies against SynGAP (an inactivator of Ras and Rap GTPases), PLK2 (Rap signaling molecule), and p-P38 (signal transducer for long-term depression). We found that AAV-DYRK1A injection significantly decreased SynGAP expression and upregulated p-P38 levels in WT mice compared to AAV-control injection but not PLK2 expression (**Figures 1H, I**, **Supplementary Figure 1D**). These data suggest that DYRK1A overexpression in the hippocampus of WT mice impairs short-term spatial/recognition memory and diminishes SynGAP levels while enhancing p-P38 levels.

### Short-term spatial and recognition memory are impaired in 5xFAD mice compared with WT mice

Given that Aβ accumulation is associated with learning and memory impairments (26, 27), we next examined whether cognitive functions are decreased in 5xFAD mice, a model of AD in which Aβ is overexpressed, compared with WT mice. We found that 3.5-month-old 5xFAD mice exhibited significant reductions in spontaneous alterations and novelty preference in the Y-maze and NOR tests compared to WT mice, indicating cognitive deficits (**Supplementary Figures 2A–D**).

### DYRK1A knockdown enhances short-term spatial/recognition memory and increases CaMKIIα-CREB signaling in 3.5-month-old 5xFAD mice

**Figure 2:**
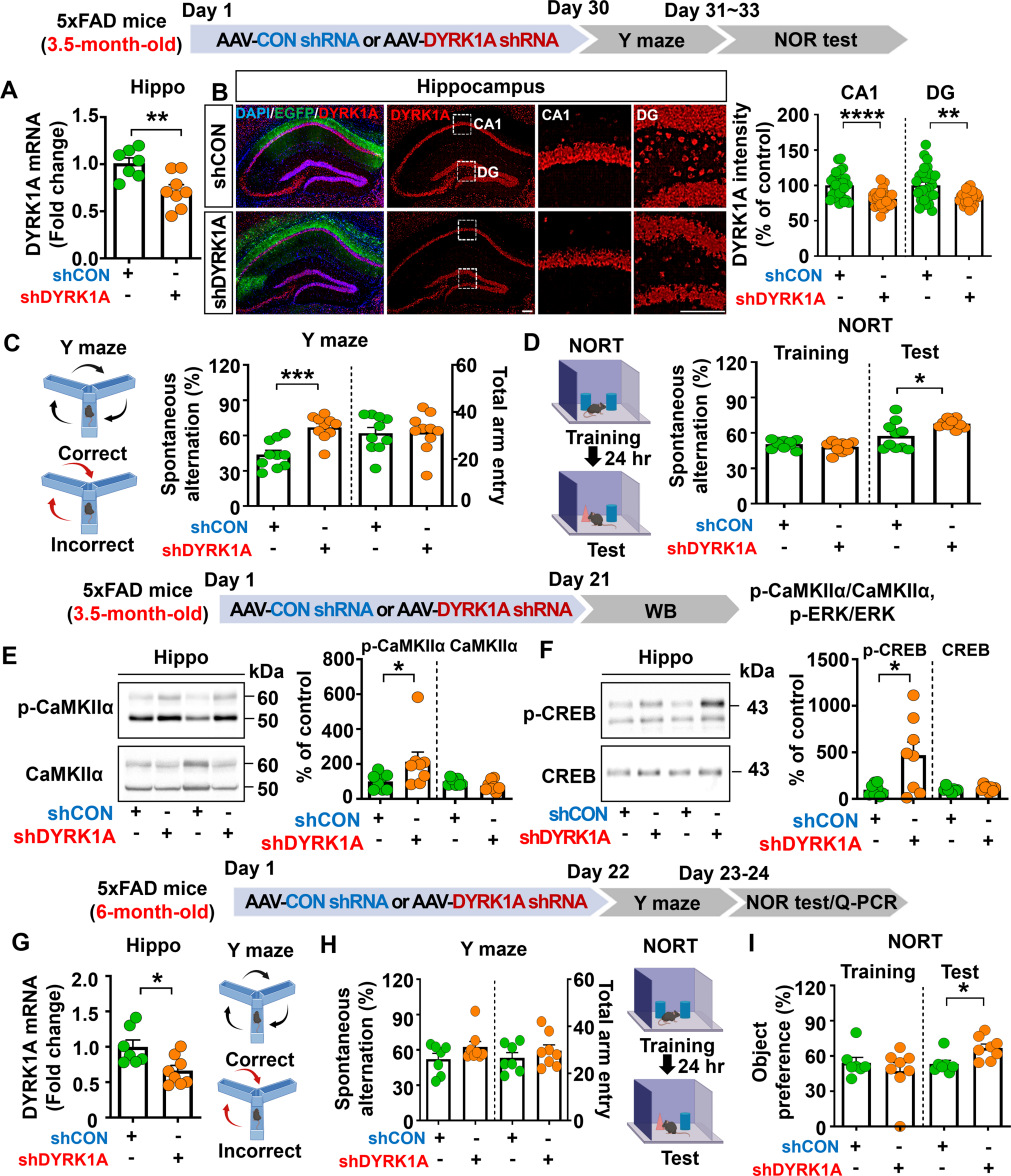
DYRK1A knockdown improves memory performance and enhances CaMKIIα/CREB phosphorylation in 5xFAD mice. **(A)** Real-time PCR analysis of hippocampal DYRK1A mRNA levels in 3.5-month-old 5xFAD mice injected with AAV-Control shRNA or AAV-DYRK1A shRNA (n = 7–8 mice/group). **(B)** Immunofluorescence staining of DYRK1A in hippocampal slices from 3.5-month-old 5xFAD mice treated as described above (n = 27–28 brain slices from 7 mice/group). An enlarged view of the hippocampal CA1 and dentate gyrus (DG) regions is indicated by the white box. **(C, D)** The results of Y-maze and NOR tests of 3.5-month-old 5xFAD mice treated as describe above (n = 10 mice/group). **(E, F)** Western blotting analysis with anti-p-CaMKIIα, anti-CaMKIIα, anti-p-CREB, and anti-CREB antibodies of hippocampal lysates from 3.5-month-old 5xFAD mice treated as described above (n = 8 mice/group). **(G)** 6-month-old 5xFAD mice were injected with AAV-Control shRNA or AAV-DYRK1A shRNA, and real-time PCR was conducted to measure hippocampal DYRK1A mRNA levels (n=7–8 mice/group). **(H, I)** Results of Y-maze and NOR tests of 6-month-old 5xFAD mice treated as describe above (n=7–8 mice/group). *p < 0.05, **p < 0.01, ***p < 0.001, ****p < 0.0001. Scale bar = 200 µm.

Given that memory function was impaired in 3.5-month-old 5xFAD mice compared with WT mice, we investigated whether direct hippocampal knockdown of DYRK1A affects learning and memory in this model of AD. To test this, 3.5-month-old 5xFAD mice were bilaterally injected with AAV-control shRNA or AAV-DYRK1A shRNA in the hippocampus. Thirty days after the injection, Y-maze and NOR tests were performed, and DYRK1A mRNA levels in brain tissue were measured. AAV-DYRK1A shRNA injection significantly suppressed DYRK1A mRNA and protein levels in 3.5-month-old 5xFAD mice compared to AAV-control shRNA injection (**Figures 2A, B**). In addition, AAV-DYRK1A shRNA-injected 5xFAD mice exhibited a significant increase in spontaneous alternation and a higher preference for the novel object compared to AAV-control shRNA-injected 5xFAD mice (**Figures 2C, D**). These data indicate that DYRK1A directly affects short-term spatial and recognition memory in 3.5-month-old 5xFAD mice.

We then examined whether direct hippocampal knockdown of DYRK1A modulates memory-associated Ras signaling in 3.5-month-old 5xFAD mice. We found that DYRK1A knockdown did not alter total levels of NMDA receptor subunits (NR2A, NR2B) and AMPA receptor subunits (GluA1, GluA2) in the hippocampus (**Supplementary Figures 3A–C**). In addition, glutamate transporter (e.g., EAAT1 and EAAT2) levels did not alter in AAV-DYRK1A shRNA-injected compared to AAV-control shRNA-injected 5xFAD mice (**Supplementary Figure 3D**). Most importantly, levels of the Ras signaling-associated molecules p-CaMKIIα and p-CREB were significantly increased in AAV-DYRK1A shRNA-injected 3.5-month-old 5xFAD mice compared to AAV-control shRNA-injected 5xFAD mice, whereas ERK phosphorylation was not altered (**Figures 2E, F**, **Supplementary Figure 3E**). These findings indicate that DYRK1A knockdown improves cognitive function accompanied by enhanced Ras signaling in 3.5-month-old 5xFAD mice.

### DYRK1A knockdown improves recognition memory in 6-month-old 5xFAD mice

Since DYRK1A knockdown improved memory performance in a mouse model of the early phase of AD (3.5-month-old 5xFAD mice), we investigated the effects of altering DYRK1A expression on cognitive function in aged 5xFAD mice. For this experiment, 6-month-old 5xFAD mice were injected with AAV-control shRNA or AAV-DYRK1A shRNA in the hippocampus. Twenty-one days after injection, Y-maze and NOR tests were performed, and hippocampal DYRK1A mRNA levels were measured. We found that DYRK1A mRNA levels were significantly reduced in AAV-DYRK1A shRNA-injected 6-month-old 5xFAD mice compared to AAV-control shRNA-injected 5xFAD mice, confirming effective gene knockdown (**Figure 2G**). In addition, DYRK1A knockdown significantly enhanced recognition memory (NOR test) but not short-term memory (Y-maze test) in 6-month-old 5xFAD mice (**Figures 2H, I**). These results indicate that DYRK1A knockdown selectively improves cognitive function in aged 5xFAD mice.

### DYRK1A knockdown significantly reduces proinflammatory cytokine levels and AD-associated reactive astrocytes and microglia in 3.5-month-old 5xFAD mice

**Figure 3:**
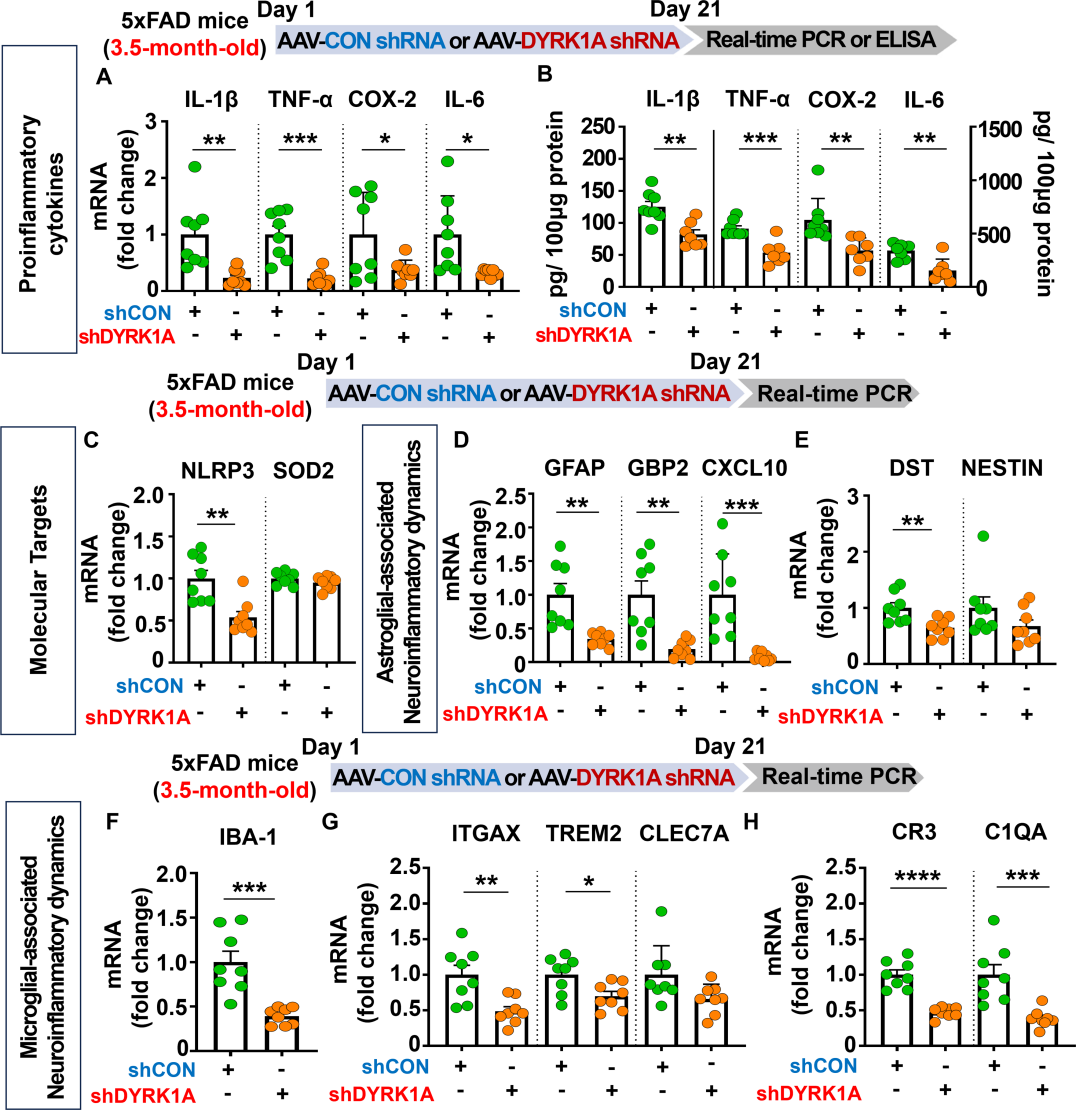
DYRK1A knockdown suppresses the expression of proinflammatory cytokines and neuroinflammatory dynamics-associated genes in 3.5-month-old 5xFAD mice. **(A, B)** 3.5-month-old 5xFAD mice were injected with AAV-Control shRNA or AAV-DYRK1A shRNA, and real-time PCR and ELISA were conducted to measure proinflammatory cytokine levels in hippocampal lysates (n = 7–8 mice/group). **(C)** Real-time PCR analysis of hippocampal NLRP3 and SOD2 mRNA levels in 3.5-month-old 5xFAD mice treated as described above (n = 8 mice/group). **(D–H)** Real-time PCR was performed in in hippocampal lysates of 3.5-month-old 5xFAD mice treated as described above to analyze mRNA levels of markers for AD-associated reactive astrocytes and microglial neuroinflammatory dynamics (n = 8 mice/group). *p < 0.05, **p < 0.01, ***p < 0.001, ****p < 0.0001.

Given that direct DYRK1A knockdown (5xFAD mice) and overexpression (WT mice) in the brain modulates cognitive function, we further examined whether AAV-DYRK1A shRNA injection alters neuroinflammatory responses/dynamics which are closely associated with memory in 5xFAD mice. To test this, 3.5-month-old 5xFAD mice were injected with AAV-control shRNA or AAV-DYRK1A shRNA in the hippocampus. Three weeks after the injection, the hippocampal tissue was dissected, and real-time PCR or ELISA was performed. We found that proinflammatory cytokine mRNA and protein levels were significantly downregulated in AAV-DYRK1A shRNA-injected 3.5-month-old 5xFAD mice compared to AAV-control shRNA-injected 5xFAD mice (**Figures 3A, B**). In addition, AAV-DYRK1A shRNA injection markedly suppressed the mRNA levels of the neuroinflammation-associated molecular target NLRP3 without altering SOD2 mRNA levels (**Figure 3C**).

Next, we examined the effects of AAV-DYRK1A shRNA injection on AD-associated glial dynamics in 3.5-month-old 5xFAD mice and found that DYRK1A knockdown significantly reduced the mRNA levels of the RA markers GFAP, GBP2, CXCL10, and DST but not NESTIN (**Figures 3D, E**). Moreover, DYRK1A knockdown significantly diminished the mRNA expression of markers for AD-related microglial dynamics (IBA-1, ITGAX, and TREM2) and RA–disease-associated microglia (DAM) interactions (CR3 and C1QA) but not CLEC7A (**Figures 3F-H**). These data suggest that direct genetic DYRK1A knockdown in the brain alleviates proinflammatory responses and AD-associated RA/microglial dynamics markers in 3.5-month-old 5xFAD mice.

### DYRK1A knockdown selectively decreases proinflammatory cytokine levels and AD-associated neuroinflammatory dynamics in 6-month-old 5xFAD mice

**Figure 4:**
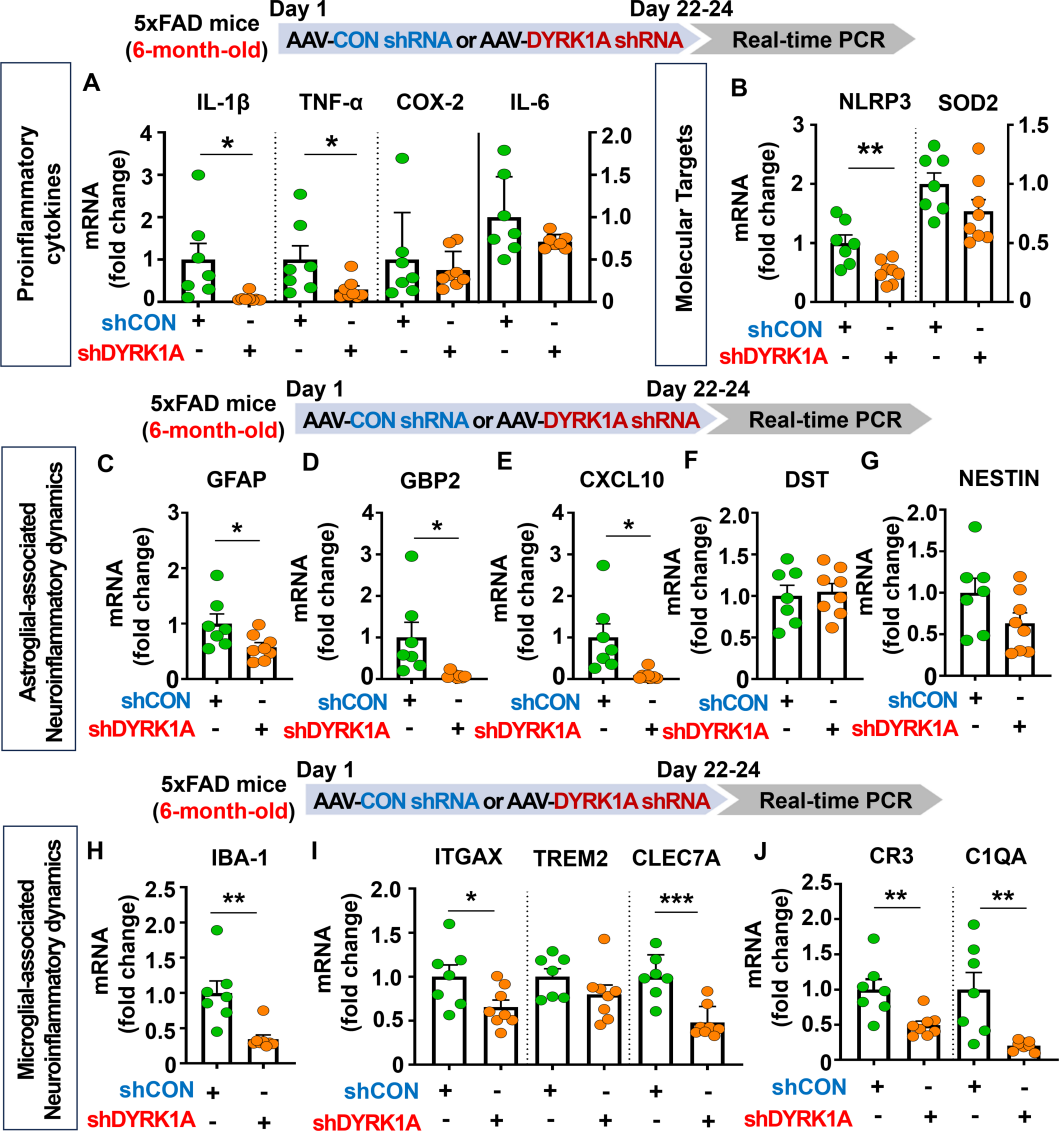
DYRK1A knockdown selectively reduces proinflammatory responses and AD-related neuroinflammatory dynamics in 6-month-old 5xFAD mice. **(A, B)** 6-month-old 5xFAD mice were injected with AAV-Control shRNA or AAV-DYRK1A shRNA, and real-time PCR was conducted to measure hippocampal proinflammatory cytokine, NLRP3 and SOD2 mRNA levels (n = 7~8 mice/group). **(C–J)** Real-time PCR analysis of the expression of markers of AD-associated reactive astrocytes and microglial neuroinflammatory dynamics in hippocampal lysates of 6-month-old 5xFAD mice treated as describe above (n =7~8 mice/group). *p < 0.05, **p < 0.01, ***p < 0.001.

Since genetic knockdown of DYRK1A downregulated neuroinflammation in 3.5-month-old 5xFAD mice, we investigated the effects of direct inhibition of DYRK1A in the brain on AD-related neuroinflammatory dynamics in aged AD mice. For this experiment, six-month-old 5xFAD mice were injected with AAV-control shRNA or AAV-DYRK1A shRNA in the hippocampus. Three weeks after the injection, the hippocampal regions were dissected, and real-time PCR was performed. We found that AAV-DYRK1A shRNA injection significantly suppressed mRNA levels of the proinflammatory cytokines IL-1β and TNF-α but not COX-2 and IL-6 in 6-month-old 5xFAD mice (**Figure 4A**). In addition, AAV-DYRK1A shRNA-treated 6-month-old 5xFAD mice significantly reduced NLRP3 mRNA levels but not SOD2 mRNA levels (**Figure 4B**). Among markers of AD-associated glial dynamics, DYRK1A knockdown significantly reduced the mRNA levels of the RA markers GFAP, GBP2, and CXCL10 in aged 5xFAD mice, but not DST and NESTIN (**Figures 4C–G**). Moreover, AAV-DYRK1A shRNA injection significantly diminished the mRNA levels of the AD-related microglial markers IBA-1, ITGAX, and CLEC7A and the RA–DAM interaction markers CR3 and C1QA in aged 5xFAD mice compared to AAV-control shRNA injection, whereas TREM2 mRNA expression was not altered (**Figures 4H–J**). These data indicate that direct genetic knockdown of DYRK1A in the brain suppresses proinflammatory cytokine levels and AD-associated neuroinflammatory dynamics markers in aged 5xFAD mice.

### DYRK1A overexpression significantly increases proinflammatory cytokine levels and neuroinflammation-associated dynamics in 3.5-month-old 5xFAD mice

**Figure 5:**
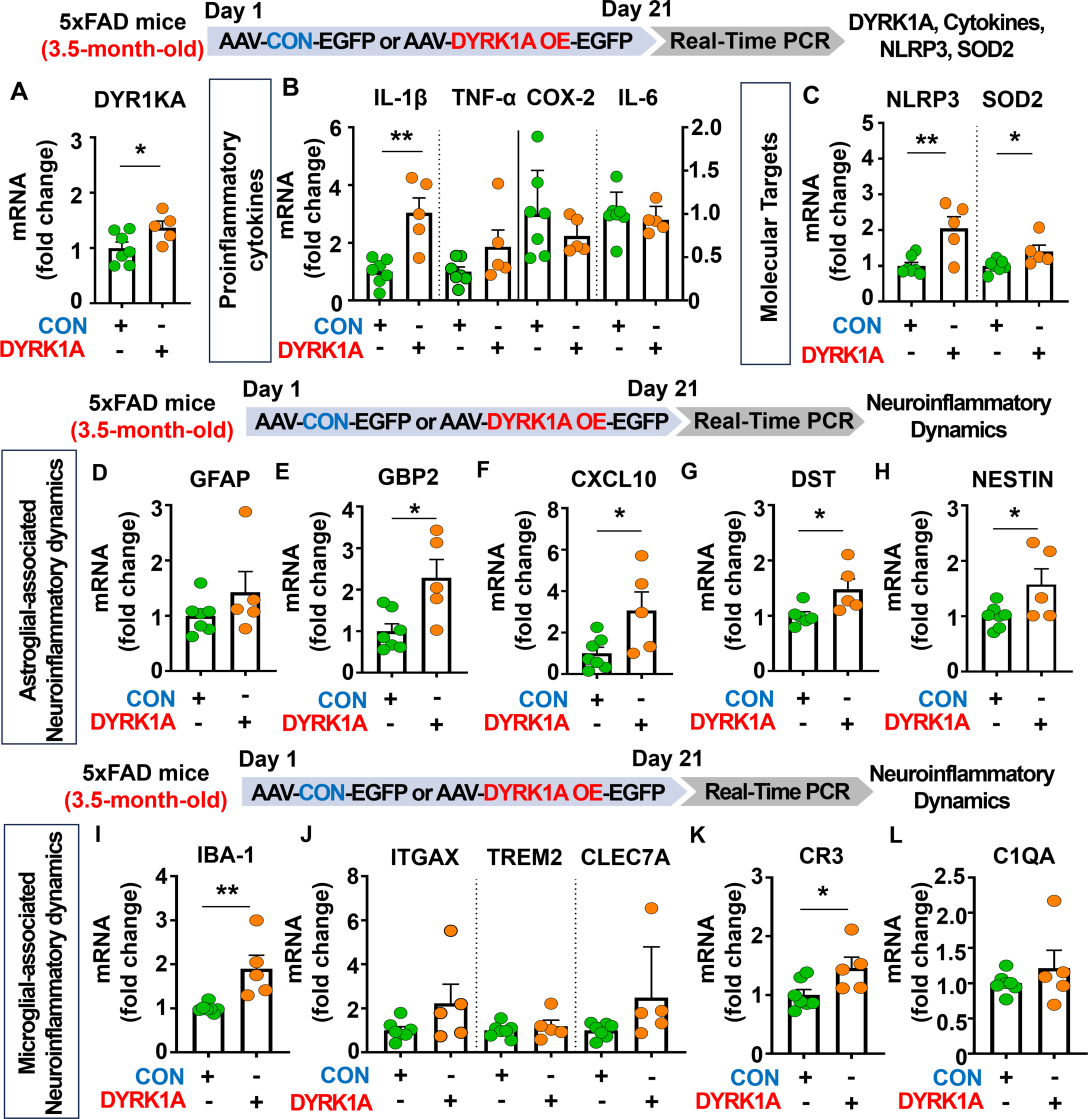
DYRK1A overexpression exacerbates the expression of proinflammatory cytokines and AD-mediated glial dynamics in 3.5-month-old 5xFAD mice. **(A)** Real-time PCR analysis of hippocampal DYRK1A mRNA levels in 3.5-month-old 5xFAD mice injected with AAV-Control or AAV-DYRK1A (n = 5–7 mice/group). **(B, C)** 3.5-month-old 5xFAD mice were treated as described above, and real-time PCR analysis of hippocampal proinflammatory cytokine, NLRP3, and SOD2 mRNA levels was performed (n = 5–7 mice/group). **(D–L)** Real-time PCR analysis of the hippocampal expression of markers of AD-associated reactive astrocytes and microglial dynamics in 3.5-month-old 5xFAD mice treated as describe above (n = 5–7 mice/group). *p < 0.05, **p < 0.01.

Since DYRK1A knockdown decreased neuroinflammation in 5xFAD mice, we determined whether direct DYRK1A overexpression in the brain modulates proinflammatory responses in this AD mouse model. For this experiment, 3.5-month-old 5xFAD mice were injected with AAV-control or AAV-DYRK1A in the hippocampus. Three weeks after the injection, the hippocampal tissue was dissected, and real-time PCR was performed. DYRK1A mRNA levels were significantly elevated in AAV-DYRK1A-injected 5xFAD mice compared with AAV-control-injected 5xFAD mice, confirming successful overexpression (**Figure 5A**). We also found that mRNA levels of the proinflammatory cytokine IL-1β, inflammation-associated molecular targets NLRP3 and SOD2 were significantly increased in AAV-DYRK1A-injected 5xFAD mice compared with AAV-control-injected 5xFAD mice but not TNF-α, COX-2 and IL-6 mRNA levels (**Figures 5B, C**).

We then investigated the effects of DYRK1A overexpression in the brain on AD-related neuroinflammatory dynamics and found that mRNA levels of AD-associated RA markers GBP2, CXCL10, DST, and NESTIN were significantly upregulated in AAV-DYRK1A-injected 3.5-month-old 5xFAD mice compared with AAV-control-injected 5xFAD mice, whereas GFAP mRNA levels were not changed (**Figures 5D–H**). Finally, DYRK1A overexpression significantly increased mRNA levels of the microglial marker IBA-1 and the RA–DAM interaction marker CR3 but not the DAM markers ITGAX, TREM2, and CLEC7A and RA–DAM interaction marker C1QA (**Figures 5I–L**), indicating that directly overexpressing DYRK1A in the brain using the AAV system selectively modulates proinflammatory cytokine levels and neuroinflammation dynamics in 3.5-month-old 5xFAD mice.

### Direct genetic modulation of DYRK1A in the brain modulates HO-1 levels and STAT3/NF-κB signaling in 3.5-month-old 5xFAD mice

**Figure 6:**
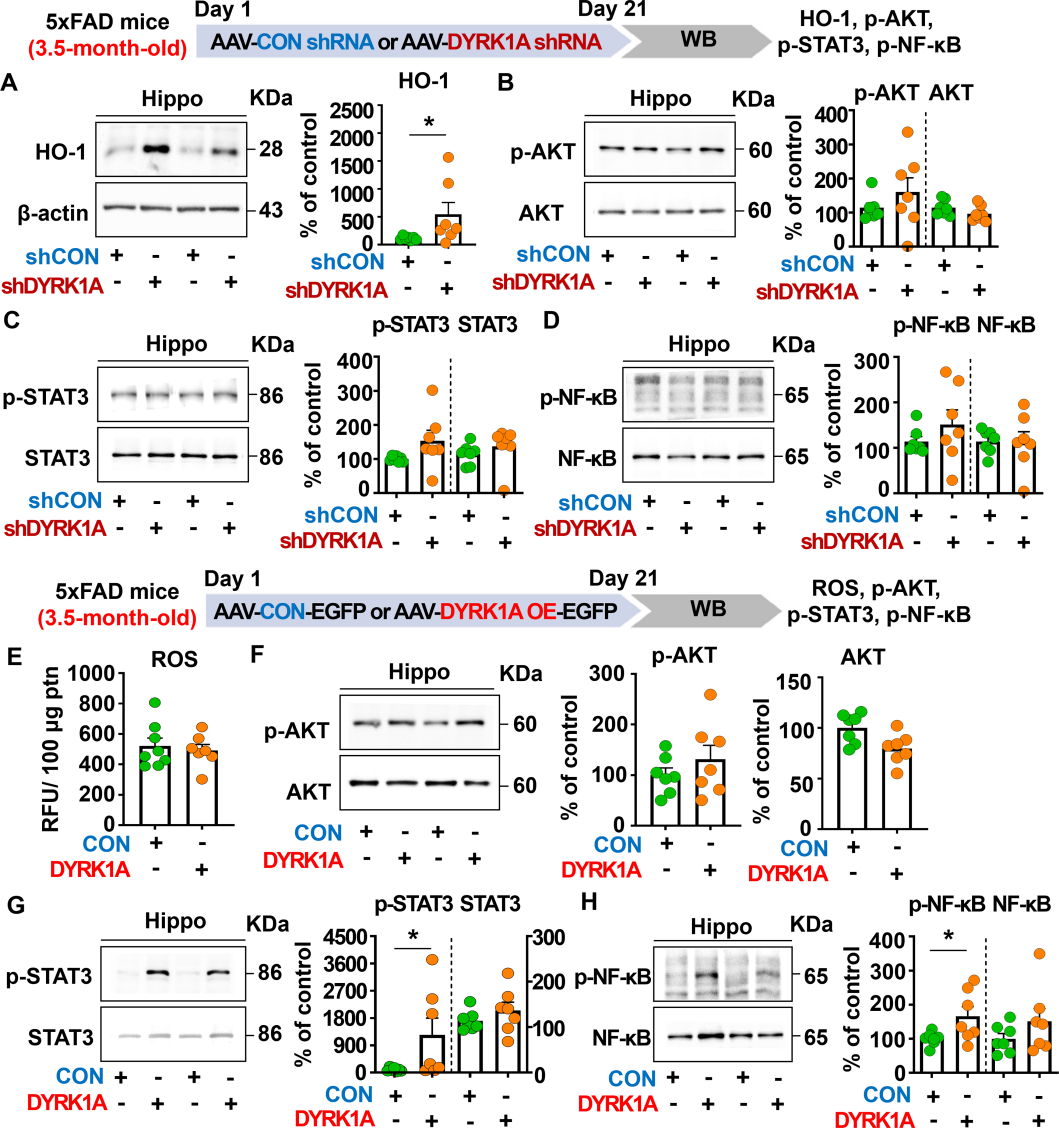
Manipulation of DYRK1A expression alters HO-1 levels and STAT3/NF-kB signaling in 3.5-month-old 5xFAD mice. **(A–D)** 3.5-month-old 5xFAD mice were injected with AAV-Control shRNA or AAV-DYRK1A shRNA (knockdown), and western blotting of hippocampal lysates was performed with anti-HO-1, anti-p-AKT, anti-AKT, anti-p-STAT3, anti-STAT3, anti-p-NF-kB, and anti-NF-kB antibodies (n = 7 mice/group). **(E)** Hippocampal ROS levels were assessed in 3.5-month-old 5xFAD mice injected with AAV-Control or AAV-DYRK1A (overexpression) (n = 7–8 mice/group). **(F-H)** Western blotting analysis of p-AKT, AKT, p-STAT3, STAT3, p-NF-kB, and NF-kB levels in hippocampal lysates from 3.5-month-old 5xFAD mice treated as described above (n=7 mice/group). *p < 0.05.

To further elucidate the molecular mechanisms by which direct genetic DYRK1A knockdown in the brain modulates neuroinflammatory responses in 5xFAD mice, 3.5-month-old 5xFAD mice were injected with AAV-control shRNA or AAV-DYRK1A shRNA in the hippocampus. Three weeks after the injection, the hippocampal tissue was dissected, and western blotting was performed with anti-HO-1, anti-p-AKT/AKT, anti-p-STAT3/STAT3, and anti-p-NF-kB/NF-kB antibodies. We found that genetic DYRK1A knockdown significantly increased protein levels of the anti-oxidative/inflammatory molecule HO-1 in 5xFAD mice compared to AAV-control shRNA injection (**Figure 6A**). However, AAV-DYRK1A shRNA-treated 5xFAD mice did not alter p-AKT, p-STAT3, and p-NF-κB levels compared to AAV-control shRNA treatment (**Figures 6B–D**).

We then investigated the effect of direct DYRK1A overexpression in the brain on oxidative stress and neuroinflammation-related downstream signaling in 3.5-month-old 5xFAD mice. The mice were injected with AAV-control-EGFP or AAV-DYRK1A-EGFP in the hippocampus, and three weeks after the injection, the hippocampal regions were dissected. Then, ROS levels were analyzed, and western blotting was performed with anti-p-AKT/AKT, anti-p-STAT3/STAT3, and anti-p-NF-κB/NF-κB antibodies. We found that DYRK1A overexpression did not affect ROS levels and AKT phosphorylation (**Figures 6E, F**). Interestingly, direct DYRK1A overexpression in the brain significantly increased p-STAT3 and p-NF-κB levels in 3.5-month-old 5xFAD mice (**Figures 6G, H**). Taken together, these results indicate that genetic modulation of DYRK1A expression in the brain differentially regulates neuroinflammation-associated downstream HO-1 and STAT3/NF-κB signaling in 5xFAD mice.

### DYRK1A knockdown reduces Aβ plaque number and soluble/insoluble Aβ levels through inhibition of BACE1 activity in 5xFAD mice

**Figure 7:**
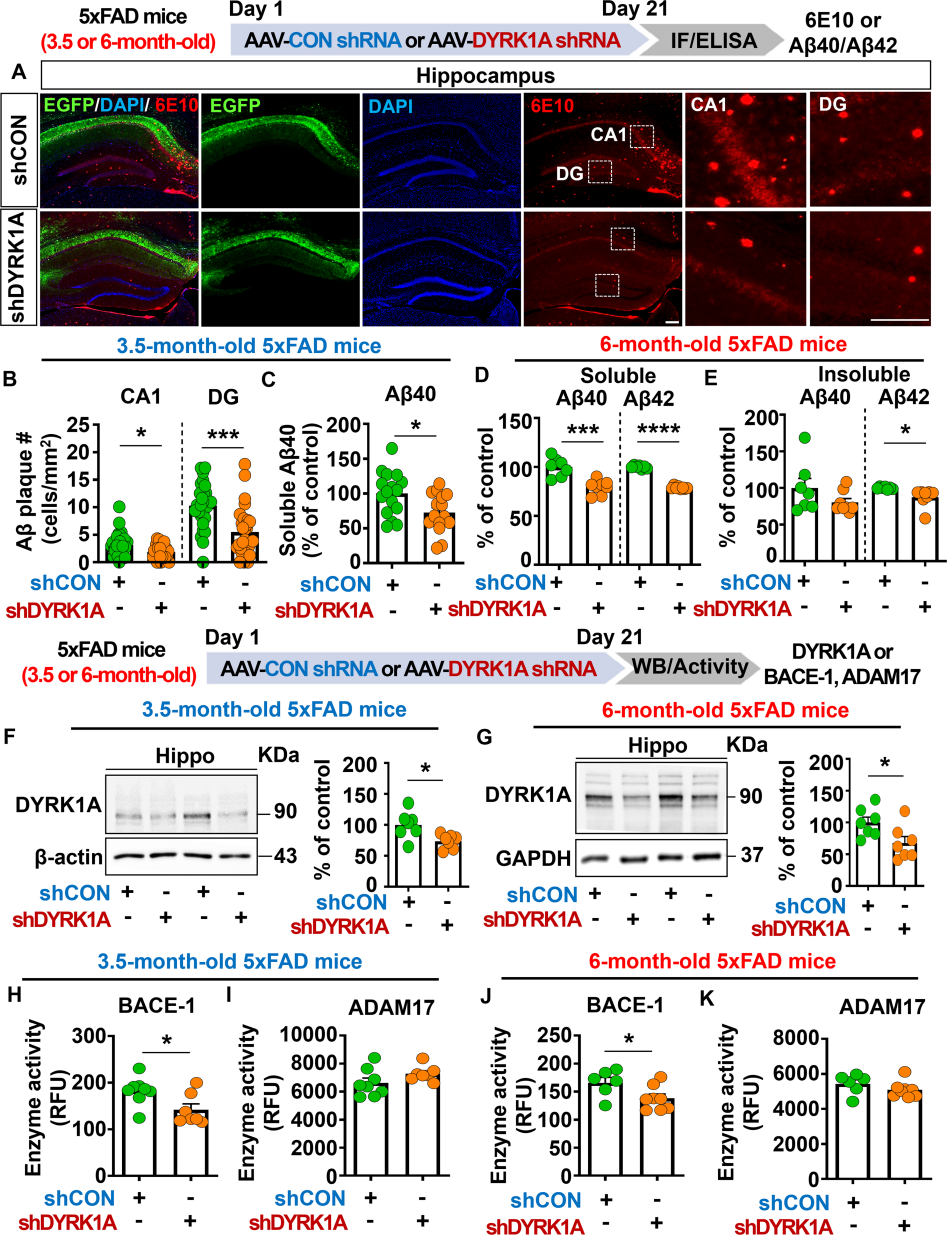
DYRK1A knockdown decreases Aβ plaque accumulation and soluble/insoluble Aβ levels by suppressing BACE activity in 5xFAD mice. **(A)** Immunofluorescence staining of 6E10-positive Aβ plaques in hippocampal slices from 3.5-month-old 5xFAD mice injected with AAV-Control shRNA or AAV-DYRK1A shRNA. **(B)** Quantification of data from **(A)** (n = 27–28 brain slices from 7 mice/group). **(C)** Results of soluble Aβ40 ELISA analysis of hippocampal lysates from 3.5-month-old 5xFAD mice treated as described above (n = 15–16 mice/group). **(D, E)** 6-month-old 5xFAD mice were injected with AAV-Control shRNA or AAV-DYRK1A shRNA, and soluble Aβ40/Aβ42 levels **(D)** and insoluble Aβ40/Aβ42 levels **(E)** in hippocampal lysates were assessed by ELISA (n=7–8 mice/group). **(F)** Western blotting analysis of hippocampal DYRK1A expression in 3.5-month-old 5xFAD mice treated as describe above (n = 7 mice/group). **(G)** Western blotting analysis of hippocampal DYRK1A expression in 6-month-old 5xFAD mice treated as described above (n=7 mice/group). **(H–K)** Hippocampal activity of BACE-1 and ADAM17 in 3.5- or 6-month-old 5xFAD mice injected with AAV-Control shRNA or AAV-DYRK1A shRNA (n = 6–8 mice/group). *p < 0.05, ***p < 0.001, ****p < 0.0001. Scale bar = 200 µm.

To investigate the effects of direct DYRK1A knockdown in the brain on Aβ pathology in a mouse model of early phase AD, 3.5-month-old 5xFAD mice were injected with AAV-control shRNA or AAV-DYRK1A shRNA in the hippocampus. Three weeks after the injection, the mice were perfused and fixed, and immunofluorescence staining of hippocampal slices was conducted with an anti-6E10 antibody. We found that DYRK1A knockdown significantly reduced Aβ plaque number in the hippocampus (**Figures 7A, B**) as well as soluble Aβ40 levels compared with AAV-control-shRNA-injected 5xFAD mice (**Figure 7C**).

We then examined whether DYRK1A knockdown alters soluble and insoluble Aβ levels in aged 5xFAD mice. To test this, six-month-old 5xFAD mice were injected with AAV-control- shRNA or AAV-DYRK1A shRNA in the hippocampus. Three weeks after the injection, soluble and insoluble fractionation and Aβ ELISA were performed. We found that soluble Aβ40 and Aβ42 levels and insoluble Aβ42 levels were significantly lowered in AAV-DYRK1A shRNA-injected 6-month-old 5xFAD mice than in AAV-control shRNA-injected mice (**Figures 7D, E**), indicating that direct genetic knockdown of DYRK1A in the brain reduces soluble and insoluble Aβ levels in aged 5xFAD mice.

To determine the molecular mechanisms by which direct DYRK1A knockdown in the brain alters Aβ pathology, we first measured protein levels of DYRK1A, which is a key player in Aβ pathology. We found that DYRK1A protein expression was significantly downregulated in AAV-DYRK1A shRNA-injected 3.5- or 6-month-old 5xFAD mice compared with AAV-control shRNA-injected 3.5- or 6-month-old 5xFAD mice (**Figures 7F, G**). Next, we measured the activities of α- and β-secretases that proteolytically process APP (ADAM17 and BACE1, respectively) and Aβ-degrading enzymes (NEP and IDE). Importantly, direct genetic knockdown of DYRK1A in the brain significantly decreased BACE1 (β-secretase) activity but not ADAM17 (α-secretase) activity in 3.5- and 6-month-old 5xFAD mice (**Figures 7H–K**). In addition, DYRK1A knockdown did not alter NEP and IDE activities in 3.5-month-old 5xFAD mice, nor did it affect protein levels of the γ-secretase PS-1-CTF (**Supplementary Figures 4A–D**). Finally, genetic DYRK1A knockdown did not affect the phosphorylation of APP at residue Thr668, which is involved in nuclear translocation of APP and further neuronal degeneration, in 3.5- and 6-month-old 5xFAD mice (**Supplementary Figures 4E–H**). These results indicate that direct genetic knockdown of DYRK1A inhibits BACE1 activity to alleviate Aβ pathology in 5xFAD mice.

### DYRK1A knockdown selectively reduces insoluble tau hyperphosphorylation in 4-month-old PS19 mice

Since DYRK1A is one of major tau kinase (20, 28), we further examined whether DYRK1A knockdown modulates tau hyperphosphorylation in 5xFAD mice. For this experiment, 3.5-month-old 5xFAD mice were injected with AAV-control shRNA or AAV-DYRK1A shRNA in the hippocampus. Three weeks later, the hippocampus was dissected, and western blotting was conducted with anti-p-Tau^Ser202/Thr205^ (AT8) and anti-p-Tau^Thr231^ (AT180) antibodies. Surprisingly, we found that DYRK1A knockdown did not affect tau hyperphosphorylation at Ser^202^/Thr^205^ (AT8) and Thr^231^ (AT180) compared to AAV-control shRNA-injected 5xFAD mice (**Supplementary Figure 5**).

**Figure 8:**
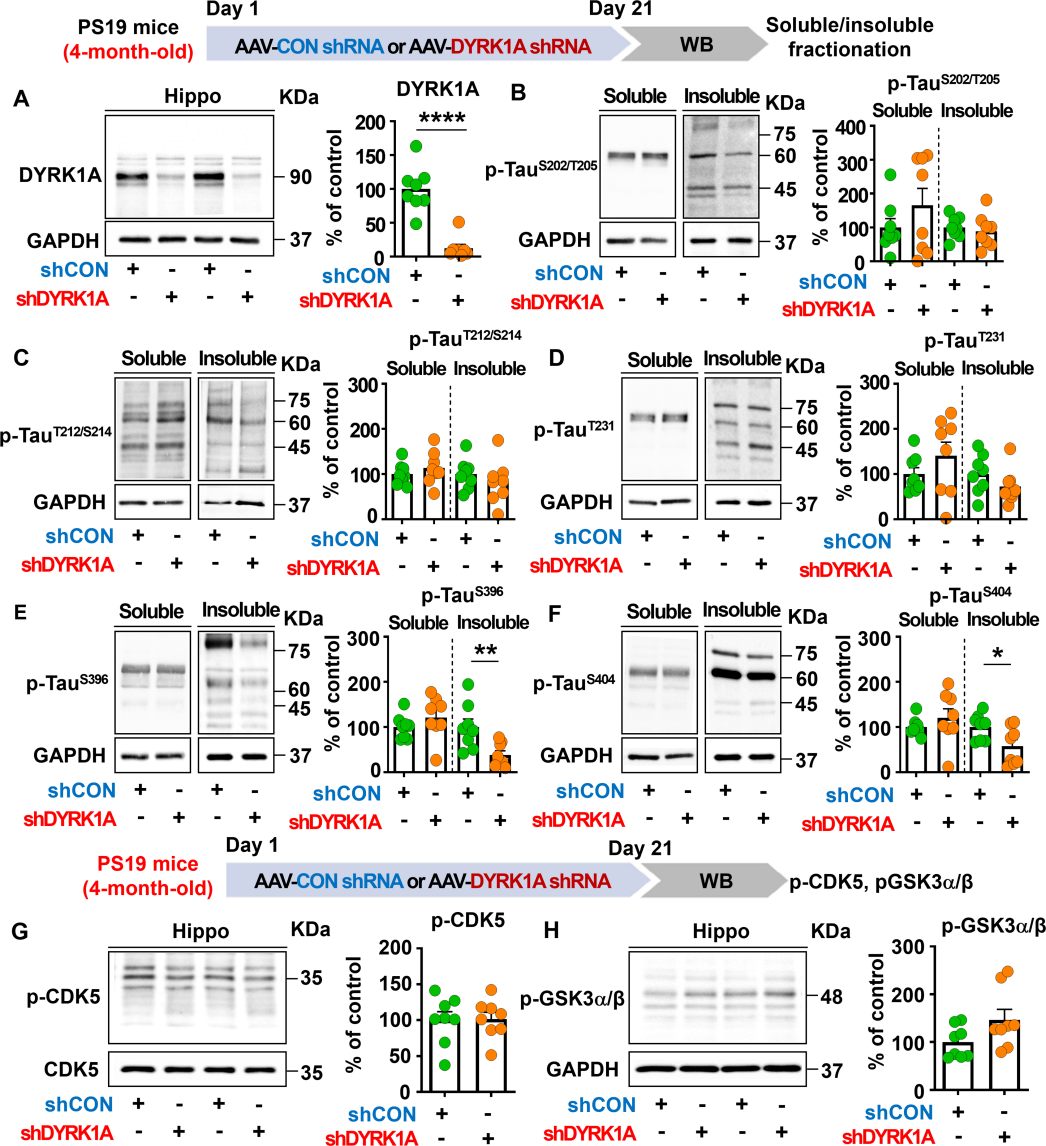
DYRK1A knockdown selectively suppresses insoluble tau hyperphosphorylation in PS19 mice. **(A)** Tau-overexpressing PS19 mice were injected with AAV-Control shRNA or AAV-DYRK1A shRNA, and western blotting of hippocampal lysates was performed with an anti-DYRK1A antibody (n = 8 mice/group). **(B–F)** PS19 mice were treated as describe above, and western blotting of hippocampal lysates was performed with anti-p-Tau^Ser202/Thr205^ (AT8), anti-p-Tau^Thr212/Ser214^ (AT100), anti-p-Tau^Thr231^ (AT180), anti-p-Tau^Ser396^, anti-p-Tau^Ser404^ and anti-GAPDH antibodies (n = 8 mice/group). **(G, H)** PS19 mice were injected as described above, and western blot analysis of hippocampal lysates was performed with anti-p-CDK5, anti-CDK5, anti-p-GSK3α/β and anti-GAPDH antibodies (n = 8 mice/group). *p < 0.05, **p < 0.01, ****p < 0.0001.

We then examined whether DYRK1A knockdown modulates tau pathology in PS19 mice, which overexpress human mutant tau. To test this, four-month-old PS19 mice were injected with AAV-control shRNA or AAV-DYRK1A shRNA in the hippocampus. Three weeks post-injection, the hippocampus was dissected, and western blotting was conducted with anti-DYRK1A, anti-p-Tau^Ser202/Thr205^ (AT8), anti-p-Tau^Thr212/Ser214^ (AT100), anti-p-Tau^Thr231^ (AT180), anti-p-Tau^Ser396^, and anti-p-Tau^Ser404^ antibodies. We found that DYRK1A knockdown significantly reduced hippocampal DYRK1A protein levels in PS19 mice compared to AAV-control shRNA-injected PS19 mice confirming successful knockdown (**Figure 8A**). In addition, DYRK1A knockdown did not alter soluble/insoluble tau hyperphosphorylation at Ser^202^/Thr^205^ (AT8), Thr^212^/Ser^214^ (AT100), or Thr^231^ (AT180) in PS19 mice (**Figures 8B–D**). Interestingly, we found that AAV-DYRK1A shRNA-injected PS19 mice but significantly downregulated insoluble tau hyperphosphorylation at Ser396 and Ser404 but not soluble p-Ser^396^ and p-Ser^404^ levels (**Figures 8E, F**).

To determine the effects of DYRK1A knockdown on p-CDK5 and p-GSK3α/β tau kinase levels, 4-month-old PS19 mice were injected as described above, and western blotting was conducted with anti-p-CDK5 and anti-p-GSK3α/β antibodies. p-CDK5 and p-GSK3α/β levels in the hippocampus did not differ between AAV-DYRK1A shRNA-injected and AAV-control shRNA-injected PS19 mice (**Figures 8G, H**), suggesting that DYRK1A knockdown directly in the brain in human tau mutant PS19 mice selectively suppresses tauopathy-associated phosphorylation without altering levels of the tau kinases CDK5 and GSK3α/β.

### DYRK1A knockdown diminishes neuroinflammatory-associated dynamics in 4-month-old PS19 mice

**Figure 9:**
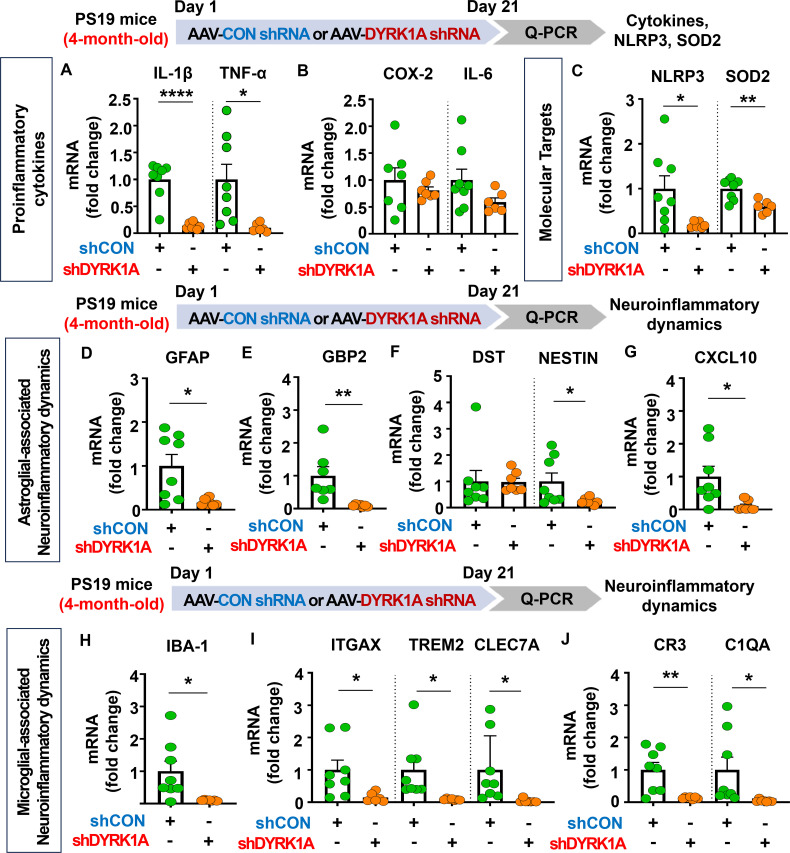
DYRK1A knockdown suppresses proinflammatory cytokine levels and AD-associated neuroinflammatory dynamics in 4-month-old PS19 mice. **(A–C)** 4-month-old PS19 mice were injected with AAV-Control shRNA or AAV-DYRK1A shRNA, and real-time PCR was conducted to measure hippocampal proinflammatory cytokine, NLRP3, and SOD2 mRNA levels (n = 6~8 mice/group). **(D–J)** Real-time PCR analysis of markers of microglial and astroglial-associated neuroinflammatory dynamics in hippocampal lysates of 4-month-old PS19 mice treated as described above (n=6-8 mice/group). *p < 0.05, **p < 0.01, ****p < 0.0001.

Since genetic knockdown of DYRK1A inhibited neuroinflammatory responses in 5xFAD mice, we investigated whether DYRK1A gene knockdown affects proinflammatory responses in PS19 mice. For this experiment, four-month-old PS19 mice were injected with AAV-control shRNA or AAV-DYRK1A shRNA in the hippocampus. Three weeks after the injection, the hippocampus regions were dissected, and real-time PCR was conducted. We found that DYRK1A knockdown significantly reduced mRNA levels of the proinflammatory cytokines IL-1β and TNF-α in PS-19 mice but not COX-2 and IL-6 (**Figures 9A, B**). In addition, AAV-DYRK1A shRNA-injected PS19 mice significantly suppressed NLRP3 and SOD2 mRNA levels (**Figure 9C**).

We then examined the effects of DYRK1A knockdown on microglial- and astroglial-associated neuroinflammatory dynamics in tau-overexpressing PS19 mice and found that markers of astrocyte-related neuroinflammatory dynamics, including GFAP, GBP2, NESTIN, and CXCL10, were reduced in AAV-DYRK1A shRNA-injected PS19 mice, whereas DST mRNA levels were unaffected (**Figures 9D–G**). Moreover, DYRK1A knockdown significantly downregulated mRNA levels of markers of microglial-associated neuroinflammatory dynamics (IBA-1), DAMs (ITGAX, TREM2, and CLEC7A), and RA–DAM interactions (CR3 and C1QA) (**Figures 9H-J**). These data indicate that direct genetic modulation of DYRK1A in the brain of tau-overexpressing PS19 mice alleviates proinflammatory responses, the expression of the neuroinflammation-related molecular targets NLRP3 and SOD2, and neuroinflammatory dynamics.

## Discussion

The role of DYRK1A as a tau kinase in AD pathogenesis is well established; however, the effects of direct genetic DYRK1A manipulation in the brain and the underlying molecular mechanisms have not been fully demonstrated. To address this gap, the present study investigated whether direct alterations in DYRK1A gene expression in the brain alter cognitive function, neuroinflammation, and Aβ/tau pathology and elucidated the underlying mechanisms of action in WT mice and/or mouse models of AD.

DYRK1A overexpression in WT mice impaired short-term spatial/recognition memory, decreased SynGAP expression, and increased P38 phosphorylation. In addition, DYRK1A knockdown in 5xFAD mice improved cognitive function, upregulated CaMKIIα/CREB signaling, and suppressed mRNA levels of markers of neuroinflammatory-associated dynamics and enhanced anti-oxidative/inflammatory molecule HO-1 levels. By contrast, DYRK1A overexpression in 5xFAD mice increased mRNA levels of markers for neuroinflammatory-associated dynamics and upregulated STAT3/NF-κB phosphorylation. Importantly, DYRK1A knockdown in 5xFAD mice reduced Aβ plaque deposition, soluble Aβ40/Aβ42 levels and insoluble Aβ42 levels by inhibiting BACE-1 activity but did not affect tau hyperphosphorylation. Furthermore, in tau-overexpressing PS19 mice, knocking down DYRK1A directly in the brain selectively suppressed insoluble tau hyperphosphorylation at Ser396 and Ser404 and neuroinflammatory responses. Collectively, the present results indicate that DYRK1A plays an important role in cognitive function, Aβ/tauopathy and neuroinflammation in WT mice and mouse models of AD, implicating DYRK1A as a potential therapeutic target for AD.

Cognitive impairments and memory loss are critical factors in AD diagnosis and progression (29). Recent studies have implicated DYRK1A is closely associated with pathoprogression of neurocognitive disorders, including AD and Down syndrome (10, 30, 31). Specifically, DYRK1A expression is increased in the brains of patients with AD or Down syndrome or in DYRK1A-overexpressing transgenic mice (DYRK1A Tg mice) compared to healthy/WT controls (32, 33). In addition, we and others have reported that pharmacological DYRK1A inhibition (e.g., with KVN93) ameliorates cognitive dysfunction and AD pathology in 3xTg AD mice and Aβ-overexpressing 5xFAD mice (22, 23). Here, we systematically investigated the direct effects of DYRK1A in the brain on cognitive function by injecting an AAV enabling DYRK1A overexpression or knockdown. In WT mice, DYRK1A overexpression significantly reduced spatial/recognition memory accompanied by decreased SynGAP (a Ras/Rap inactivator) expression and increased p-P38 levels (**Figure 1**). However, DYRK1A knockdown significantly increased short-term and long-term memory as assessed by the Y-maze and NOR tests, respectively in 3.5-month-old 5xFAD mice (**Figure 2**). Furthermore, DYRK1A knockdowned 6-month-old 5xFAD mice also significantly enhanced long-term memory but not short-term memory (**Figure 2**). These stage-dependent differences may reflect variations in AD severity, as 3.5- and 6-month-old 5xFAD mice represent the early and intermediate stages of AD, respectively. More importantly, DYRK1A knockdown rescued cognitive function and increased CaMKIIα/CREB signaling in 5xFAD mice compared with AAV-control shRNA-injected 5xFAD mice (**Figure 2**). Our findings raise an interesting question: why do genetic overexpression and knockdown of DYRK1A engage distinct memory-regulating pathways? These differences may be the result of distinct neuropathological states in WT and 5xFAD mice. Specifically, under pathological AD conditions (Aβ overexpression in 5xFAD mice), Aβ oligomers suppress LTP-promoting Ras signaling, including CaMKIIα activation and CREB-dependent transcription, thereby contributing to memory impairment (34, 35). Consistent with previous findings, we found that DYRK1A knockdown reduced Aβ levels in 5xFAD mice (**Figure 7**), which may have attenuated Aβ-mediated inhibition of the CaMKIIα–CREB pathway, thereby restoring pathway activity and improving memory performance (**Figure 2**). However, under non-pathological conditions (WT mice), CaMKIIα–CREB signaling is already within a normal functional range. Thus, it is possible that DYRK1A overexpression does not further alter CaMKIIα or CREB phosphorylation in WT mice. Instead, DYRK1A overexpression reduced SynGAP expression and increased p-P38 levels in WT mice (**Figure 1**). Because SynGAP inactivates Ras/Rap signaling, SynGAP deficiency is closely associated with cognitive impairment. Indeed, SynGAP1^+/−^ mice exhibit attenuated hippocampal LTP induction and reduced learning and memory (36). In addition, excessive activation of P38 disrupts synaptic plasticity and memory; Dai et al. demonstrated that neuron-specific knockdown of P38 restored hippocampal LTP and improved spatial memory performances in 5xFAD mice (37). Moreover, pharmacological inhibition of P38 (e.g., by NJK14047 or MW150) elicits neuroprotective effects and/or enhanced cognitive function in 5xFAD mice (38, 39). Taken together, our findings suggest that genetic DYRK1A manipulation modulates cognitive function through disease state–dependent mechanisms: restoring Aβ-suppressed CaMKIIα–CREB signaling under AD pathological conditions (5xFAD mice) or modulating SynGAP–P38 pathways under normal conditions (WT mice).

Homeostatic astrocytes and surveilling microglia play critical roles in the formation and remodeling of synapses, thereby contributing to normal cognitive function (40, 41). However, under pathological conditions, including sustained exposure to Aβ plaques and/or NFTs, these neuroprotective glial cells shift to disease-associated reactive glia, which exacerbate neuroinflammation and contribute to neuronal degeneration followed by cognitive decline (42–44). Importantly, several studies have demonstrated that DYRK1A plays an essential role in neuroinflammatory responses *in vivo* (22, 45). For instance, DYRK1A-overexpressing Tg mice and DYRK1A shRNA plasmid-injected WT mice exhibit significant increases or decreases, respectively, in the mRNA levels of the astrocyte markers GFAP and S100β (45). The same study also found that the expression of MAC-2, a marker of AD-associated reactive microglia, is not altered in DYRK1A Tg mice (45, 46). In addition, we previously reported that pharmacological inhibition of DYRK1A with the small molecule KVN93 significantly downregulates microglial and astrocyte activation in 5xFAD mice (22). However, the effects of altering DYRK1A gene expression directly in the brain on neuroinflammatory dynamics are not well studied. We therefore investigated the effect of genetic DYRK1A manipulation directly in the brain on microglial/astroglial neuroinflammatory dynamics and the underlying mechanisms of action in 5xFAD mice. We found that AAV-DYRK1A shRNA injection (knockdown) significantly decreased neuroinflammatory responses and significantly increased HO-1 expression in 5xFAD mice without altering STAT3/NF-κB phosphorylation levels (**Figures 3**, **6**). By contrast, AAV-DYRK1A injection (overexpression) notably increased proinflammatory responses and elevated STAT3 and NF-κB phosphorylation in 5xFAD mice but did not affect ROS levels (**Figures 5**, **6**). The distinct mechanisms underlying these differential effects of DYRK1A knockdown and overexpression on oxidative stress and neuroinflammatory downstream signaling in 5xFAD mice will be systematically analyzed in a future study.

The NLRP3 inflammasome plays a key role in AD progression by increasing the release of the proinflammatory cytokine IL-1β and reducing Aβ phagocytosis, which accelerates Aβ aggregation and senile plaque deposition (47). Interestingly, pharmacological inhibition of NLRP3 by OLT1177 ameliorates Aβ accumulation and cognitive impairment in an AD mouse model (48, 49). Although both NLRP3 and DYRK1A have been implicated in AD pathology, the mechanistic relationship has not been fully elucidated. We therefore examined whether direct modulation of DYRK1A in the brain affects NLRP3 expression in 5xFAD mice. AAV-DYRK1A shRNA injection (knockdown) significantly decreased NLRP3 mRNA levels in the hippocampus in 5xFAD mice, whereas AAV-DYRK1A-injection (overexpression) markedly increased NLRP3 mRNA expression (**Figures 3**–**5**). These results suggest that DYRK1A regulates NLRP3 to influence neuroinflammatory responses in this mouse model of AD. Consistent with this possibility, DYRK1A knockdown increased levels of the anti-oxidative/neuroinflammatory molecule HO-1, while DYRK1A overexpression upregulated STAT3/NF-κB signaling, which is associated with NLRP3 downstream signaling in 5xFAD mice (**Figure 6**). Collectively, these findings raise the possibility that DYRK1A may function upstream of NLRP3 to modulate neuroinflammatory responses in AD pathology. To further validate this hypothesis, it is necessary to determine whether directly altering DYRK1A expression (knockdown or overexpression) in the brain modulates key upstream modulators of NLRP3 [e.g., thioredoxin-interacting protein (TXNIP) and NIMA-related kinase 7 (NEK7)]. Changes in TXNIP and NEK expression would support the notion that DYRK1A acts upstream of NLRP3. Future studies will clarify this regulatory relationship. Alternatively, DYRK1A may act downstream of NLRP3 to diminish AD-associated neuroinflammatory signaling. To test this possibility, future research will examine whether modulation of NLRP3 expression via genetic knockdown using an AAV vector system or pharmacological inhibition alters DYRK1A levels or activity in mouse models of AD. A third plausible explanation is that DYRK1A directly binds to NLRP3 or its adaptor proteins (e.g., ASC), thereby influencing inflammasome assembly and subsequent proinflammatory cytokine release. Taken together, our findings suggest that DYRK1A and NLRP3 reciprocally regulate each other through a bidirectional signaling network to modulate neuroinflammatory responses in mouse models of AD.

Neurotoxic Aβ plaques are formed through the amyloidogenic proteolytic processing of APP by β-secretase (BACE-1) and γ-secretase (presenilin), which contributes to neuronal degeneration and further synaptic and cognitive dysfunction (4, 13). In contrast, Aβ production is inhibited when APP is processed via non-amyloidogenic proteolysis by α-secretases such as ADAM10 and ADAM17 (50). In addition, Aβ-degrading enzymes like IDE and NEP hydrolyze Aβ_40_ into smaller and less toxic fragments (51). Interestingly, several studies have reported that DYRK1A participates in the regulation of APP trafficking and processing, thereby contributing to Aβ pathology *in vitro* and *in vivo*. For example, DYRK1A modulates bilateral APP axonal transportation, a critical process for Aβ pathogenesis, in neurons derived from human induced pluripotent stem cells (52). Moreover, DYRK1A Tg mice that overexpress DYRK1A exhibit increased phosphorylation of APP at Thr688, a crucial site for amyloidogenic processing and Aβ levels in the brain (53). Furthermore, pharmacological inhibition of DYRK1A, e.g., by KVN93, significantly reduces Aβ plaque accumulation and insoluble Aβ40/Aβ42 levels in 5xFAD mice and 3xTg mice (22, 23). In the present study, we demonstrated that AAV-DYRK1A shRNA-injected 5xFAD mice significantly reduced Aβ deposition and soluble/insoluble Aβ levels through selectively reducing the activity of the β-secretase BACE-1 without affecting other Aβ-regulating enzymes (i.e., ADAM17, NEP, and IDE) or PS-1 expression levels (**Figure 7**, **Supplementary Figure 4**). We then examined whether directly altering DYRK1A expression in the brain modulates APP phosphorylation at Thr688 to affect Aβ pathology and found that direct DYRK1A knockdown in the brain did not alter p-APP^Thr688^ levels in either 3.5- or 6-month-old 5xFAD mice (**Supplementary Figure 4**). Together, the previous literature and the present findings suggest that DYRK1A manipulation directly in the brain diminishes Aβ pathology by suppressing BACE-1 activity and/or direct inhibition of DYRK1A itself in 5xFAD mice. Although our current findings demonstrate the underlying mechanisms by which direct genetic DYRK1A knockdown or overexpression in the brain modulates Aβ pathology in WT and 5xFAD mice, we did not use pharmacological inhibitors to further validate whether DYRK1A manipulation modulates Aβ pathologies by targeting other molecules, which will be addressed in a future study.

Tau participates in microtubule stabilization in neurons under normal physiological conditions. However, under pathological conditions, multiple tau kinases (e.g., DYRK1A and GSK3α/β) hyperphosphorylate tau, leading to its aggregation, NFT formation, and cognitive dysfunction (54, 55). Therefore, tau kinase dysfunction contributes to the pathogenesis of neurodegenerative diseases, and *in vivo* and clinical studies have shown that modulating tau kinase is a critical therapeutic approach. For example, genetic DYRK1A overexpression (e.g., in DYRK1A transgenic mice or Down syndrome patients) increases total tau levels, tau hyperphosphorylation, and NFT formation (56, 57). However, pharmacological DYRK1A inhibition significantly reduces tauopathy in 3xTg mice, a mouse model of AD exhibiting both Aβ pathology and tauopathy (23). In addition, CDK5 is highly expressed in the brains of patients with AD, and genetic overexpression of CDK5 or increased CDK5 activity induces NFT formation, synaptic damage, and neuronal death *in vitro* and *in vivo* (58, 59). Another tau kinase, GSK3, is associated with memory decline, tau hyperphosphorylation, and the formation of paired helical filaments (60, 61). However, beyond these findings, the effect of direct genetic inhibition of DYRK1A in the brain on tauopathy has not been fully investigated in mouse models of AD. We found that AAV-DYRK1A shRNA injection in the brain in 5xFAD did not reduce tau hyperphosphorylation at Ser^202^/Thr^205^ and Thr^231^ (**Supplementary Figure 5**). To understand why direct DYRK1A knockdown in the brain does not affect tau hyperphosphorylation in 3.5-month-old 5xFAD mice, it is important to remember that tau hyperphosphorylation increases in an age-dependent manner in 5xFAD mice. While robust p-Tau (Ser202/Thr205) expression is observed in 7- to 8-month-old 5xFAD mice (late-stage AD), it is possible that 3.5-month-old 5xFAD mice (early-stage AD) are not suitable for assessing the effect of DYRK1A knockdown on tau hyperphosphorylation (62, 63). To further clarify the effects of DYRK1A on tau pathology, we examined whether direct DYRK1A knockdown in the brain differentially regulates tau hyperphosphorylation in tau-overexpressing PS19 mice. We found that tau phosphorylation at Ser396 and Ser404 was significantly reduced in RIPA-insoluble fractions of hippocampal tissue from AAV-DYRK1A shRNA-injected PS19 mice (**Figure 8**). These results indicate that direct DYRK1A knockdown in the brain modulates tau hyperphosphorylation under tauopathy-predominant conditions.

There are several limitations of the present study. First, wexdemonstrated that genetic DYRK1A knockdown did not alter tau phosphorylation in 3.5-month-old 5xFAD mice (**Supplementary Figure 5**) and selectively reduced insoluble tau hyperphosphorylation at Ser396 and Ser404 in 4-month-old PS19 mice (**Figure 8**). Therefore, combined approaches might provide a broader blockade of tau phosphorylation epitopes, thereby achieving more efficient suppression of tauopathy and directly and/or indirectly regulating Aβ pathology. CDK5 and GSK3β are involved in inflammation/Aβ signaling as well as synaptic plasticity/cognitive function (64–67). Therefore, combining genetic knockdown of DYRK1A with a tau inhibitor might have synergistic effects on multiple aspects of AD pathology, including cognitive impairment, neuroinflammation, and Aβ/tau pathology. Second, the present study specifically focused on the effect of genetic manipulation of DYRK1A in the hippocampus rather than multiple brain regions. The hippocampus was chosen because it plays a pivotal role in early memory formation and is particularly vulnerable to AD-related pathology (68). Given its central involvement in memory consolidation and synaptic plasticity, we examined how DYRK1A knockdown or overexpression in this region influences cognitive function and other AD pathologies. However, we are aware that other brain regions, such as the cortex, are also crucial for regulating learning and memory. Future studies will therefore investigate the effects of DYRK1A modulation in the cortex using AAV-based gene delivery to determine its impact on cognitive function, AD pathology, and neuroinflammation in mouse models of AD as well as explore potential combinational therapeutic synergistic effects (e.g., DYRK1A gene therapy and Aβ/tau inhibitor) on AD pathology.

## Conclusion

The present study demonstrated that DYRK1A gene overexpression directly in the hippocampus in WT mice significantly impaired short-term spatial/recognition memory by modulating SynGAP and P38 signaling. In addition, DYRK1A knockdown directly in the hippocampus in Aβ-overexpressing 5xFAD mice significantly attenuated cognitive impairment and neuroinflammatory responses, increased anti-oxidative/anti-inflammatory HO-1 levels, and reduced Aβ pathology by suppressing BACE-1 activity. Moreover, DYRK1A overexpression directly in the hippocampus in 5xFAD mice exacerbated neuroinflammation and enhanced STAT3/NF-kB signaling. Furthermore, DYRK1A knockdown directly in the hippocampus in tau-overexpressing PS19 mice selectively reduced insoluble tau phosphorylation and proinflammatory responses/glial dynamics. These results indicate that modulation of DYRK1A expression in the brain is a promising therapeutic strategy for ameliorating cognitive dysfunction and mitigating AD-related pathologies.

## Data Availability

The original contributions presented in the study are included in the article/**Supplementary Material**. Further inquiries can be directed to the corresponding author.
