# Supplementary material for: Genetic knockdown of DYRK1A attenuates cognitive impairment, Aβ pathology, tauopathy and neuroinflammatory responses in mouse models of AD
Source: Front Immunol. 2025 Nov 11;16:1661791. doi: 10.3389/fimmu.2025.1661791 (PMC12643867; doi:10.3389/fimmu.2025.1661791)
Supplement: Supplementary file 1 [file DataSheet1.docx]

**Genetic knockdown of DYRK1A attenuates cognitive impairment, Aβ pathology, tauopathy and neuroinflammatory responses in mouse models of AD**

**Hyun-ju Lee^a,b,^**^e^**| Sora Kang^a,b,e^ | Yoo Jin Lee^a,e^| Seokjun Oh^a,b,e^| Bitna Joo^a,c^| Jeong-Woo Hwang^a,b^| Jeongseop Kim^a,d^| Tae-Eun Kim^a^|** **Tae-Mi Jung^b^| Yu-Jin Kim^b^| Ji-Yeong Jang^a,b,c^| Jeong-Heon Song^b^| Ja Wook Koo^a,c,^*| Hyang-Sook Hoe^a,b,c,^***

^a^ Department of Neural Development and Disease, Korea Brain Research Institute (KBRI), 61, Cheomdan-ro, Dong-gu, Daegu, Republic of Korea, 41068; ^b^ AI-based Neurodevelopmental Diseases Digital Therapeutics Group, Korea Brain Research Institute (KBRI), 61, Cheomdan-ro, Dong-gu, Daegu, Republic of Korea, 41068;^c^ Department of Brain Sciences, Daegu Gyeongbuk Institute of Science & Technology, Daegu, Republic of Korea 42988; ^d^ Department of Pharmacology, School of Medicine, Daegu Catholic University, Daegu 42472, Republic of Korea. ^e^ These authors contributed equally to this work.

*** Running title**: The effect of genetic knockdown of DYRK1A in AD

***Corresponding authors**

**Hyang-Sook Hoe**, Ph.D.: Department of Neural Development and Disease, Korea Brain Research Institute (KBRI), 61 Cheomdan-ro, Dong-gu, Daegu, Korea, 41068; AI-based Neurodevelopmental Diseases Digital Therapeutics Group, Korea Brain Research Institute (KBRI), 61, Cheomdan-ro, Dong-gu, Daegu, Republic of Korea, 4106; E-mail: [sookhoe72@kbri.re.kr](mailto:sookhoe72@kbri.re.kr)

**Ja Wook Koo**, Ph.D.: Department of Neural Development and Disease, Korea Brain Research Institute (KBRI), 61, Cheomdan-ro, Dong-gu, Daegu 41062; E-mail: [Jawook.koo@kbri.re.kr](mailto:Jawook.koo@kbri.re.kr)


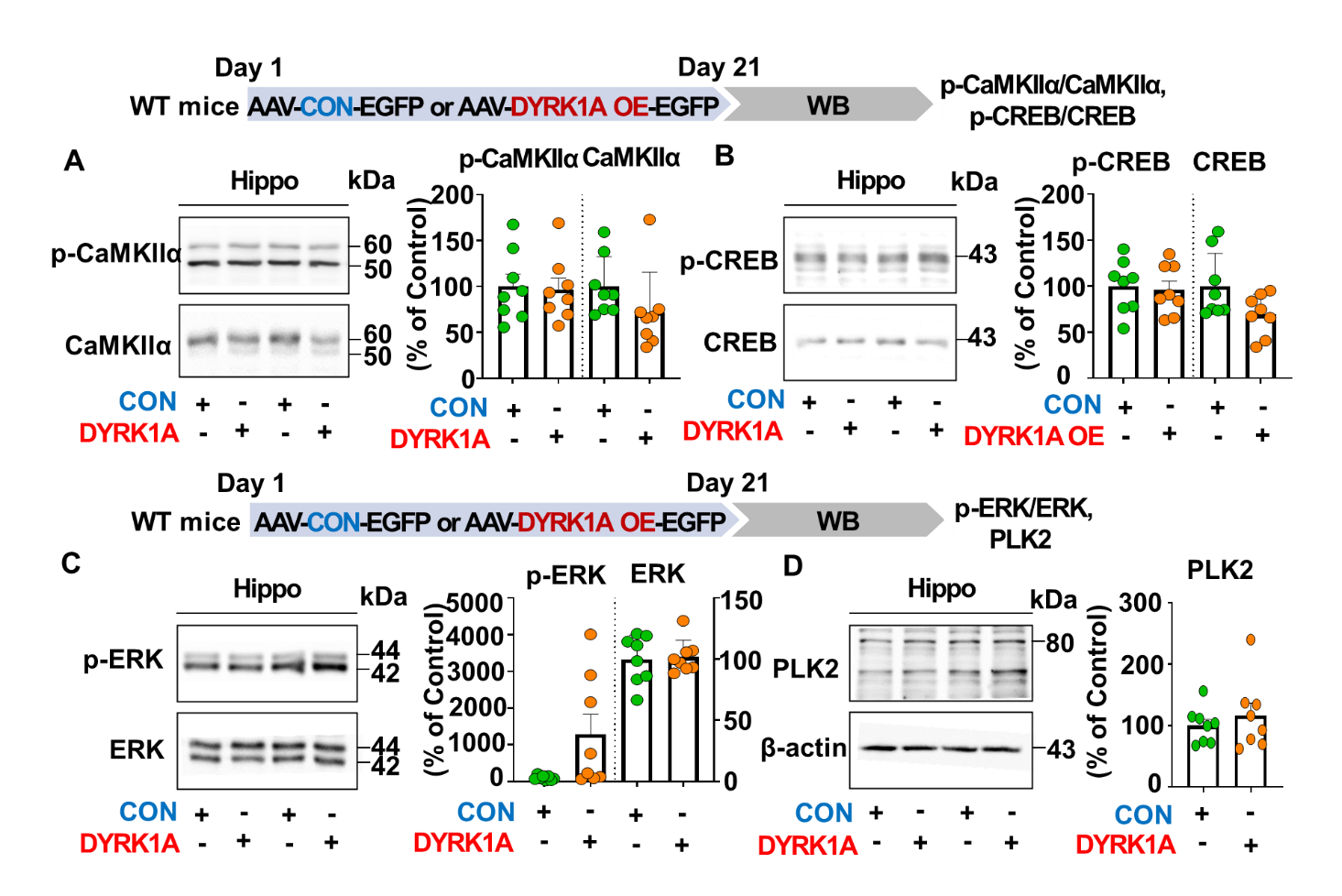
**Supplementary Fig. 1. AAV-DYRK1A-treated WT mice does not alter CaMKIIα/CREB/ERK signaling and PLK2.** (**A-D**) WT mice were injected with AAV-Control-EGFP or AAV-DYRK1A-EGFP, and western blotting of hippocampal lysates was conducted with anti-p-CaMKIIα and anti-CaMKIIα antibodies (**A**, n = 8 mice/group); anti-p-CREB and anti-CREB antibodies (**B**, n = 8 mice/group); anti-p-ERK and anti-ERK antibodies (**C**, n = 8 mice/group); and anti-PLK2 and anti-β-actin antibodies (**D**, n = 8 mice/group).

**
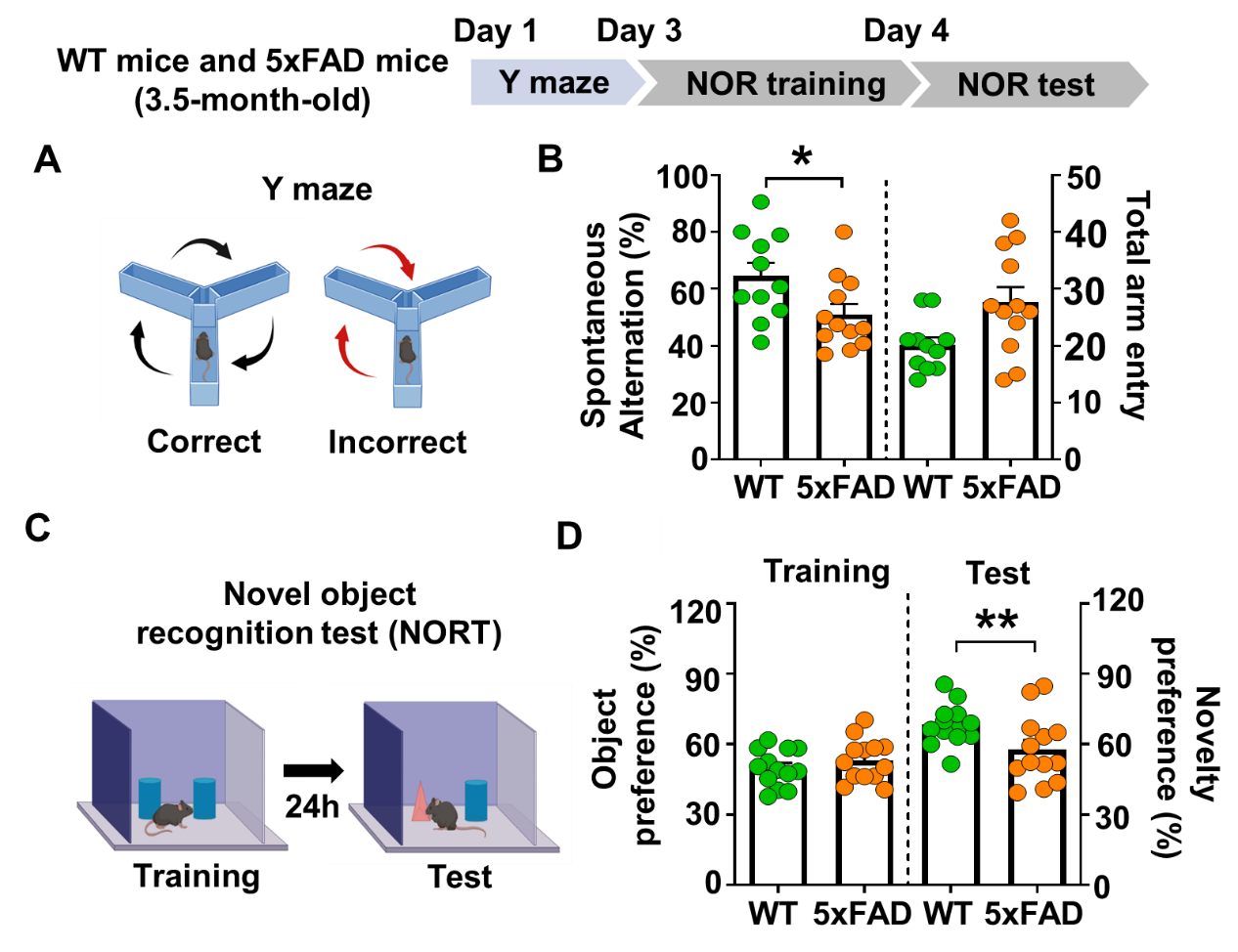
Supplementary Fig. 2. Short-term and recognition memory are impaired in 5xFAD mice compared with WT mice.** (**A-D)** The results of Y-maze and NOR tests from 3.5-month-old WT mice and 5xFAD mice (Y maze: WT mice, n = 11 mice, 5xFAD mice, n = 12 mice; NOR test: n = 13 mice/group). *p<0.05, **p<0.01


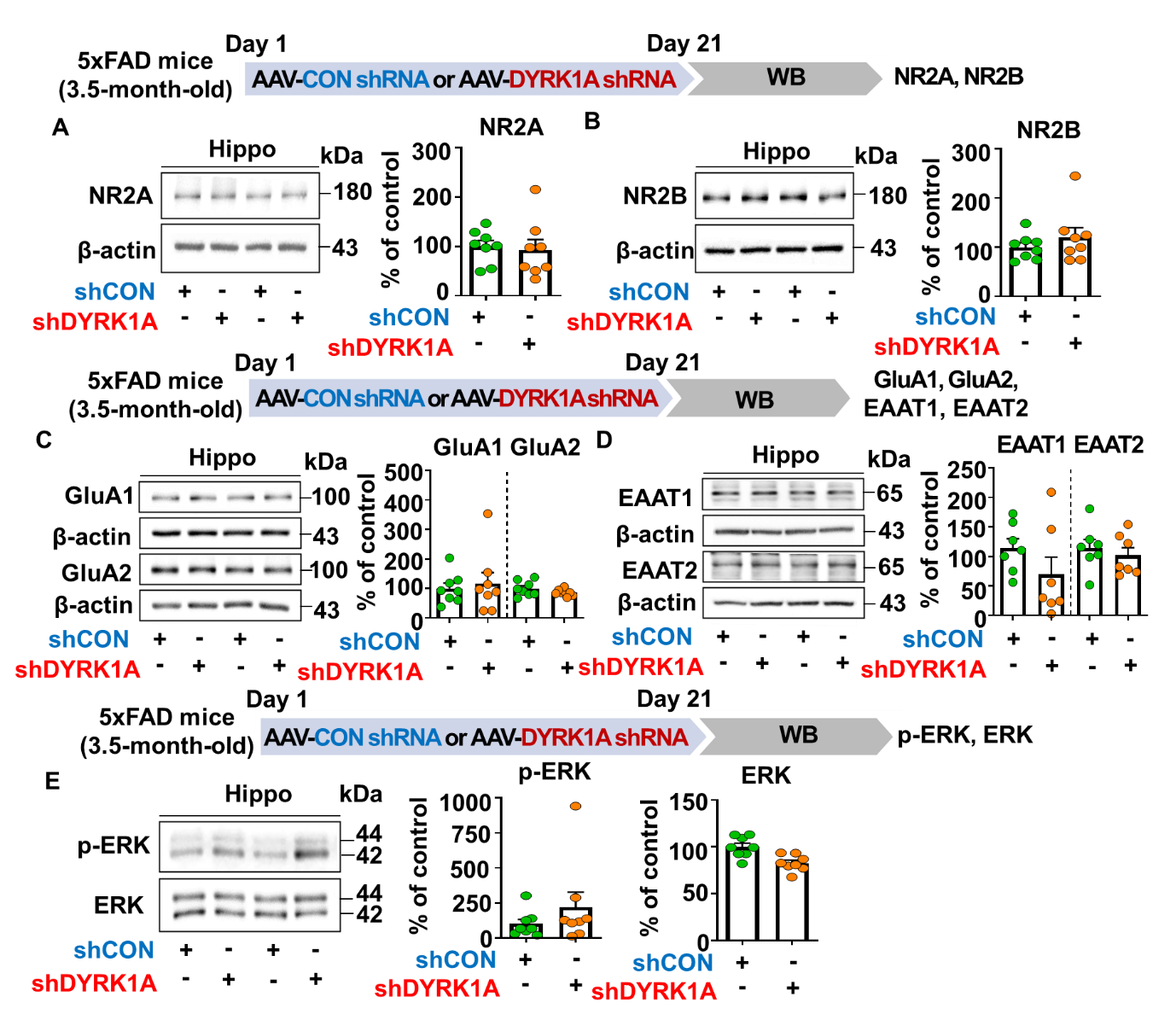
**Supplementary Fig. 3. AAV-DYRK1A shRNA-treated 5xFAD mice does not alter NMDA/AMPA receptor, EAAT1/EAAT2, or ERK phosphorylation levels.** (**A**) 5xFAD mice were injected with AAV-Control shRNA or AAV-DYRK1A shRNA, and western blotting of hippocampal lysates was performed with anti-NR2A and anti-β-actin antibodies (**A**, n = 8 mice/group); anti-NR2B and anti-β-actin antibodies (**B**, n = 8 mice/group); anti-GluA1, anti-GluA2 and anti-β-actin antibodies (**C**, n = 8 mice/group); anti-EAAT1, anti-EAAT2 and anti-β-actin antibodies (**D**, n = 7 mice/group); and anti-p-ERK and anti-ERK antibodies (**E**, n = 8 mice/group).


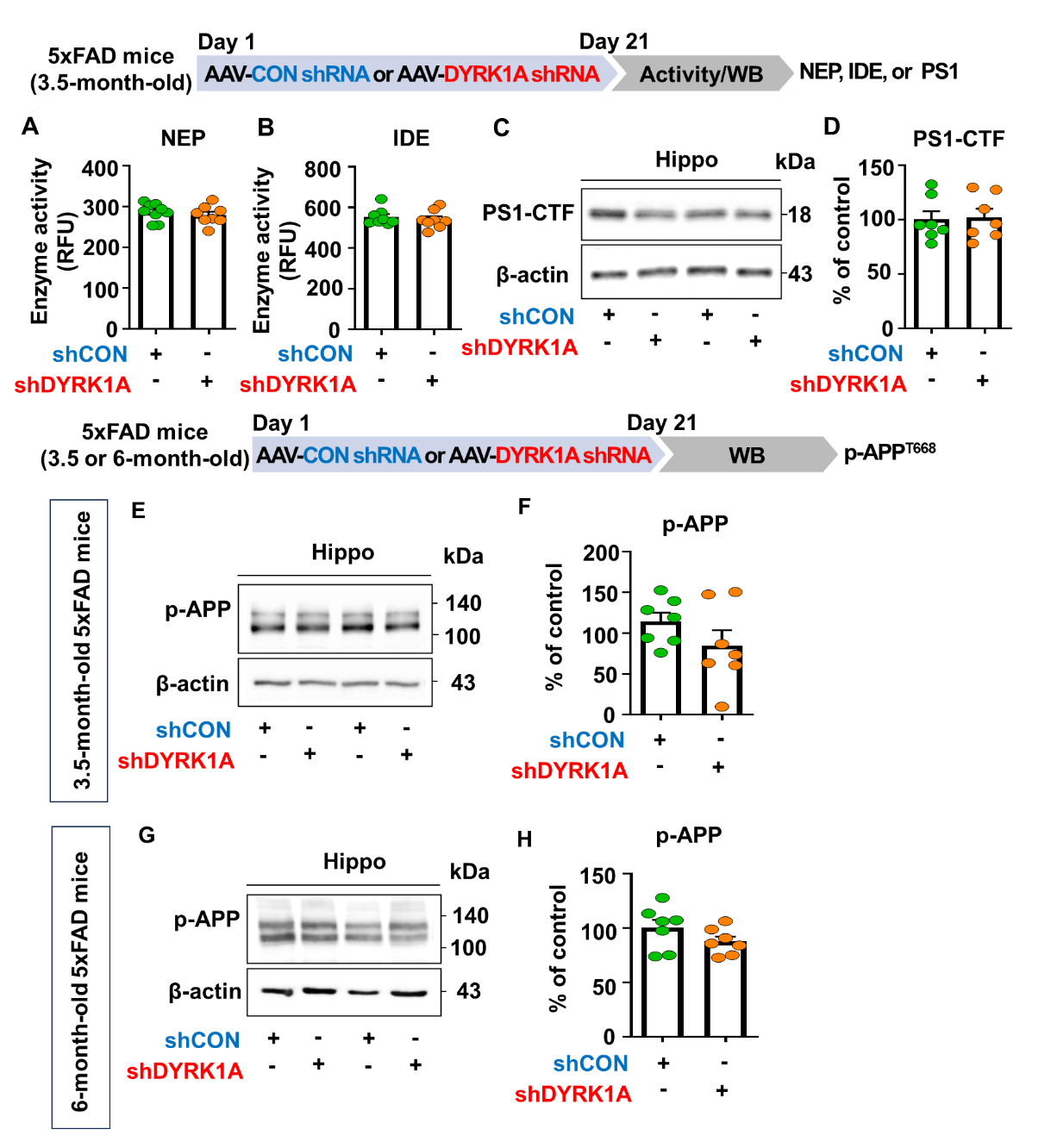
**Supplementary Fig. 4. AAV-DYRK1A shRNA-treated 5xFAD mice does not modulate NEP and IDE activity, PS1-CTF protein levels, or APP phosphorylation.** (**A-B**) 3.5-month-old 5xFAD mice were injected with AAV-Control shRNA or AAV-DYRK1A shRNA, and hippocampal NEP and IDE activities were measured (n = 8-9 mice/group). (**C-D**) 3.5-month-old 5xFAD mice were treated as described above, and western blotting of hippocampal lysates was conducted with anti-PS-1-CTF and anti-β-actin antibodies (n = 7 mice/group). (**E-H**) 3.5- and 6-month-old 5xFAD mice were injected with AAV-Control shRNA or AAV-DYRK1A shRNA, and western blotting of hippocampal lysates was conducted with anti-p-APP and anti-β-actin antibodies (n = 7 mice/group).

**
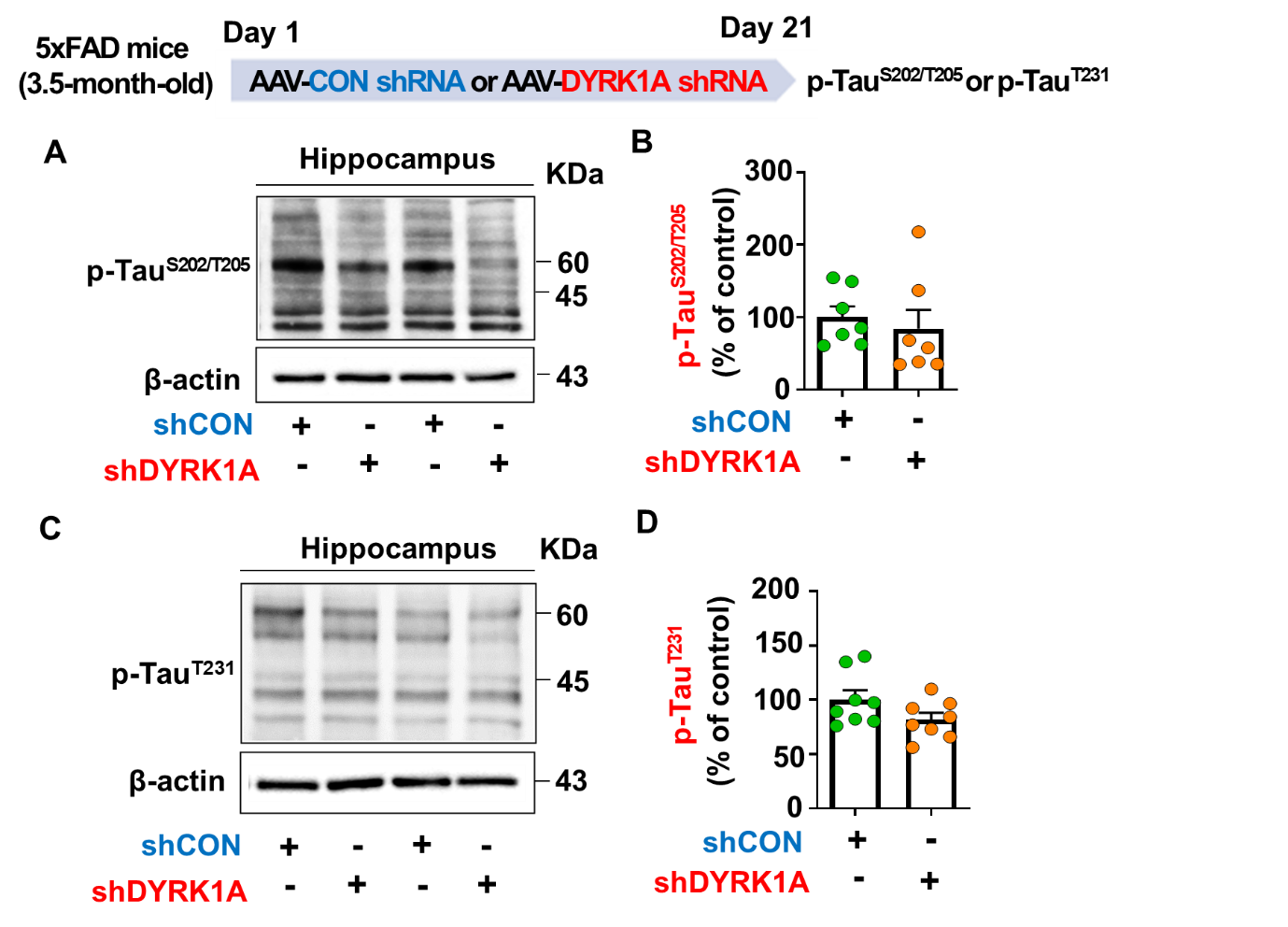
Supplementary Fig. 5. AAV-DYRK1A-shRNA-treated 5xFAD mice does not alter tau hyperphosphorylation at Ser202/Thr205 and Thr231 residues.** (**A-D**) 5xFAD mice were injected with AAV-Control shRNA or AAV-DYRK1A shRNA, and western blotting of hippocampal lysates was conducted with anti-Tau^Ser202/Thr205^ (detected by AT8), anti-Tau^Thr231^ (detected by AT180) and anti-β-actin antibodies (n = 7 mice /group).

**Supplementary Table 1.** Sequences of primers used for real-time PCR.

| Gene |  | Sequence |
| --- | --- | --- |
| DYRK1A | Forward | 5’-CTG AGA ACA TCC TTC TGT GTA ACC-3’ |
|  | Reverse | 5’- GTA GCA CCT CTG GAG ACC GAT A -3’ |
| IL-1β | Forward | 5’-TTG ACG GAC CCC AAA AGA TG-3’ |
|  | Reverse | 5’-AGG ACA GCC CAG GTC AAA G -3’ |
| TNF-α | Forward | 5’- TCC AGG CGG TGC CTA TGT-3’ |
|  | Reverse | 5’- GCC CCT GCC ACA AGC A-3’ |
| COX-2 | Forward | 5’- CCA CTT CAA GGG AGT CTG G-3’ |
|  | Reverse | 5’- AGT CAT CTG CTA CGG GAG GA-3’ |
| IL-6 | Forward | 5’-CCA CGG CCT TCC CTA CTT C -3’ |
|  | Reverse | 5’-TTG GGA GTG GTA TCC TCT GTG A -3’ |
| NLRP3 | Forward | 5’-TCC ACA ATT CTG ACC CAC AA-3’ |
|  | Reverse | 5’-ACC TCA CAG AGG GTC ACC AC-3’ |
| SOD2 | Forward | 5’- GGC CAA GGG AGA TGT TAC AA-3’ |
|  | Reverse | 5’- GAA CCT TGG ACT CCC ACA-3’ |
| GFAP | Forward | 5’-GTT TCA TCT TGG AGC TTC TGC-3’ |
|  | Reverse | 5’-GGA GGT GGA GAG GGA CAA C-3’ |
| GBP2 | Forward | 5’-GGGGTCACTGTCTGACCACT |
|  | Reverse | 5’-GGGAAACCTGGGATGAGATT |
| CXCL10 | Forward | 5’- GCC GTC ATT TTC TGC CTC A-3’ |
|  | Reverse | 5’- GCT TCC CTA TGG CCC TCA TT-3’ |
| NESTIN | Forward | 5’-AGG AGA AGC AGG GTC TAC AGA G-3’ |
|  | Reverse | 5’-AGT TCT CAG CCT CCA GCA GAG T-3’ |
| DST | Forward | 5’-GCT CCC TGC TCA GAA AAC AC-3’ |
|  | Reverse | 5’-CTG TGA ACG TGT GGA TGG TC-3’ |
| IBA-1 | Forward | 5’-GGA CAG ACT GCC AGC CTA AG-3’ |
|  | Reverse | 5’-GAC GGC AGA TCC TCA TCA TT-3’ |
| ITGAX | Forward | 5’- CCA AGA CAT CGT GTT CCT GAT T-3’ |
|  | Reverse | 5’- ACA GCT TTA ACA AAG TCC AGC A-3’ |
| CLEC7A | Forward | 5’-AGA CAC AGG GAG AAG GCA AA-3’ |
|  | Reverse | 5’-CAG AAA AGG TGG GCA GAC TC-3’ |
| TREM2 | Forward | 5’- CAG TTT CTC CTG CTG CTG AT-3’ |
|  | Reverse | 5’- CAG TGC TTC AAG GCG TCA TA-3’ |
| CR3 | Forward | 5’- GCA GGA GTC GTA TGT GAG G-3’ |
|  | Reverse | 5’- TTA CTG AGG TGG GGC GTC T-3’ |
| C1QA | Forward | 5’- AAA GGC AAT CCA GGC AAT ATC A -3’ |
|  | Reverse | 5’- TGG​ TTC ​TGG ​TAT ​GGA ​CTC ​TCC-3’ |
| GAPDH | Forward | 5’- TGT GTC CGT CGT GGA TCT GA-3’ |
|  | Reverse | 5’- CCT GCTTCA CCA CCT TCT TGA -3’ |

**Supplementary Table 2**. Primary and secondary antibodies used for immunofluorescence staining.

| **Primary antibodies** | | | | | | | |  |
| --- | --- | --- | --- | --- | --- | --- | --- | --- |
| **Immunogen** | **Host Species** | | **Manufacturer** | | **Catalog No.** | | **Dilution** |  |
| DYRK1A | Rabbit | | Abcam | | Ab180910 | | 1:200 |  |
| 6E10 | Mouse | | BioLegend | | 803002 | | 1:500 |  |
| **Secondary antibodies** | | | | | | | |  |
| **Antibody** | | **Manufacturer** | | **Catalog No.** | | **Dilution** | |  |
| Goat anti-rabbit IgG, 555 | | Invitrogen | | A21428 | | 1:200 | |  |
| Goat anti-mouse IgG, 555 | | Invitrogen | | A21422 | | 1:200 | |  |

**Supplementary Table 3**. Primary and secondary antibodies used for western blotting.

| **Primary antibodies** | | | | | | | |  |
| --- | --- | --- | --- | --- | --- | --- | --- | --- |
| **Immunogen** | **Host Species** | | **Manufacturer** | | **Catalog No.** | | **Dilution** |  |
| DYRK1A | Rabbit | | Abcam | | Ab180910 | | 1:1000 |  |
| SynGAP | Rabbit | | Cell Signaling | | 3200 | | 1:1000 |  |
| p-P38 | Rabbit | | Cell Signaling | | 9211 | | 1:1000 |  |
| P38 | Rabbit | | Cell Signaling | | 9212 | | 1:1000 |  |
| p-CaMKIIα | Rabbit | | Cell Signaling | | 12716 | | 1:1000 |  |
| CaMKIIα | Rabbit | | Abcam | | Ab92332 | | 1:1000 |  |
| p-CREB | Rabbit | | Cell Signaling | | 9198 | | 1:1000 |  |
| CREB | Rabbit | | Cell Signaling | | 9197 | | 1:1000 |  |
| PLK2 | Rabbit | | Novus | | NBP2-15078 | | 1:20000 |  |
| HO-1 | Rabbit | | Cell Signaling | | 70081 | | 1:1000 |  |
| p-AKT | Rabbit | | Cell Signaling | | 9271 | | 1:1000 |  |
| AKT | Rabbit | | Cell Signaling | | 9272 | | 1:1000 |  |
| p-STAT3 | Rabbit | | Cell Signaling | | 9145 | | 1:1000 |  |
| STAT3 | Rabbit | | Cell Signaling | | 9139 | | 1:1000 |  |
| p-NF-κB | Rabbit | | Cell Signaling | | 3033 | | 1:1000 |  |
| NF-κB | Rabbit | | Cell Signaling | | 8242 | | 1:1000 |  |
| NR2A | Rabbit | | Cell Signaling | | 4205 | | 1:1000 |  |
| NR2B | Rabbit | | Abcam | | Ab65783 | | 1:1000 |  |
| GluA1 | Rabbit | | Cell Signaling | | 13185 | | 1:1000 |  |
| GluA2 | Rabbit | | Cell Signaling | | 13607 | | 1:1000 |  |
| EAAT1 | Rabbit | | Cell Signaling | | 4166 | | 1:1000 |  |
| EAAT2 | Rabbit | | Cell Signaling | | 3838 | | 1:1000 |  |
| p-ERK | Rabbit | | Cell Signaling | | 9101 | | 1:4000 |  |
| ERK | Rabbit | | Cell Signaling | | 9102 | | 1:4000 |  |
| PS-1-CTF | Rabbit | | Cell Signaling | | 5643 | | 1:1000 |  |
| p-APP^Thr668^ | Rabbit | | Cell Signaling | | 3823 | | 1:1000 |  |
| p-Tau^S202/T205^(AT8) | Mouse | | Invitrogen | | MN1020 | | 1:500 |  |
| p-Tau^T212/S214^(AT100) | Mouse | | Invitrogen | | MN1060 | | 1:500 |  |
| p-Tau^T231^(AT180) | Mouse | | Invitrogen | | MN1040 | | 1:1000 |  |
| p-Tau^Ser396^ | Rabbit | | Invitrogen | | 44-752G | | 1:1000 |  |
| p-Tau^Ser404^ | Rabbit | | Invitrogen | | 44-758G | | 1:1000 |  |
| p-GSK3α/β | Rabbit | | Abcam | | ab75745 | | 1:1000 |  |
| p-CDK5 | Rabbit | | MybioSource | | MBS2534755 | | 1:1000 |  |
| GAPDH | Rabbit | | Proteintech | | 10494-1-AP | | 1:5000 |  |
| β-actin | Mouse | | Santa Cruz | | SC-47778 | | 1:5000 |  |
| **Secondary antibodies** | | | | | | | |  |
| **Antibody** | | **Manufacturer** | | **Catalog No.** | | **Dilution** | |  |
| Goat anti-rabbit, HRP | | Enzo | | ADI-SAB-300-J | | 1:5000 | |  |
| Goat anti-mouse, HRP | | Enzo | | ADI-SAB-100-J | | 1:5000, 1:10000 | |  |

**Supplementary Table 4**. Statistical analysis results for the *in vivo* experiments in this study.

| **Figure 1B. DYRK1A intensity in AAV-Con or AAV-DYRK1A-treated WT mice** |
| --- |
| \| Table Analyzed \| Figure 1B \| \| --- \| --- \| \|  \|  \| \| Column B \| DYRK1A OE \| \| vs. \| vs. \| \| Column A \| Con \| \|  \|  \| \| Unpaired t test \|  \| \| P value \| <0.0001 \| \| P value summary \| **** \| \| Significantly different (P < 0.05)? \| Yes \| \| One- or two-tailed P value? \| Two-tailed \| \| t, df \| t=6.367, df=30 \| |
| **Figure 1C. DYRK1A mRNA level in AAV-Con or AAV-DYRK1A-treated WT mice** |
| \| Table Analyzed \| Figure 1B \| \| --- \| --- \| \|  \|  \| \| Column A \| Con \| \| vs. \| vs. \| \| Column B \| DYRK1A OE \| \|  \|  \| \| Unpaired t test \|  \| \| P value \| 0.0008 \| \| P value summary \| *** \| \| Significantly different (P < 0.05)? \| Yes \| \| One- or two-tailed P value? \| Two-tailed \| \| t, df \| t=5.241, df=8 \| |
| **Figure 1E. DYRK1A level in AAV-Con or AAV-DYRK1A-treated WT mice** |
| \| Table Analyzed \| Figure 1B \| \| --- \| --- \| \|  \|  \| \| Column B \| DYRK1A OE \| \| vs. \| vs. \| \| Column A \| Con \| \|  \|  \| \| Unpaired t test \|  \| \| P value \| 0.0452 \| \| P value summary \| * \| \| Significantly different (P < 0.05)? \| Yes \| \| One- or two-tailed P value? \| Two-tailed \| \| t, df \| t=2.198, df=14 \| |
| **Figure 1F. Y-maze, Spontaneous alternation in AAV-Con or AAV-DYRK1A-treated WT mice** |
| \| Table Analyzed \| Figure 1C \| \| --- \| --- \| \|  \|  \| \| Column A \| Con \| \| vs. \| vs. \| \| Column B \| DYRK1A OE \| \|  \|  \| \| Unpaired t test \|  \| \| P value \| <0.0001 \| \| P value summary \| **** \| \| Significantly different (P < 0.05)? \| Yes \| \| One- or two-tailed P value? \| Two-tailed \| \| t, df \| t=7.357, df=43 \| |
| **Figure 1F. Y-maze, Total arm entry in AAV-Con or AAV-DYRK1A-treated WT mice** |
| \| Table Analyzed \| Figure 1C \| \| --- \| --- \| \|  \|  \| \| Column C \| Con \| \| vs. \| vs. \| \| Column D \| DYRK1A OE \| \|  \|  \| \| Unpaired t test \|  \| \| P value \| 0.0736 \| \| P value summary \| ns \| \| Significantly different (P < 0.05)? \| No \| \| One- or two-tailed P value? \| Two-tailed \| \| t, df \| t=1.834, df=43 \| |
| **Figure 1G. NOR Training in AAV-Con or AAV-DYRK1A-treated WT mice** |
| \| Table Analyzed \| Figure 1D \| \| --- \| --- \| \|  \|  \| \| Column A \| Con \| \| vs. \| vs. \| \| Column B \| DYRK1A OE \| \|  \|  \| \| Unpaired t test \|  \| \| P value \| 0.6678 \| \| P value summary \| ns \| \| Significantly different (P < 0.05)? \| No \| \| One- or two-tailed P value? \| Two-tailed \| \| t, df \| t=0.4321, df=43 \| |
| **Figure 1G. NOR Test in AAV-Con or AAV-DYRK1A-treated WT mice** |
| \| Table Analyzed \| Figure 1D \| \| --- \| --- \| \|  \|  \| \| Column C \| Con \| \| vs. \| vs. \| \| Column D \| DYRK1A OE \| \|  \|  \| \| Unpaired t test \|  \| \| P value \| <0.0001 \| \| P value summary \| **** \| \| Significantly different (P < 0.05)? \| Yes \| \| One- or two-tailed P value? \| Two-tailed \| \| t, df \| t=6.244, df=43 \| |
| **Figure 1H. SynGAP level in AAV-Con or AAV-DYRK1A-treated WT mice** |
| \| Table Analyzed \| Data 1 \| \| --- \| --- \| \|  \|  \| \| Column B \| Data Set-B \| \| vs. \| vs. \| \| Column A \| Data Set-A \| \|  \|  \| \| Unpaired t test \|  \| \| P value \| 0.0099 \| \| P value summary \| ** \| \| Significantly different (P < 0.05)? \| Yes \| \| One- or two-tailed P value? \| One-tailed \| \| t, df \| t=2.628, df=14 \| |
| **Figure 1I. p-P38 level in AAV-Con or AAV-DYRK1A-treated WT mice** |
| \| Table Analyzed \| Data 1 \| \| --- \| --- \| \|  \|  \| \| Column B \| Data Set-B \| \| vs. \| vs. \| \| Column A \| Data Set-A \| \|  \|  \| \| Unpaired t test \|  \| \| P value \| 0.0196 \| \| P value summary \| * \| \| Significantly different (P < 0.05)? \| Yes \| \| One- or two-tailed P value? \| One-tailed \| \| t, df \| t=2.275, df=14 \| |
| **Figure 1I. p-P38 level in AAV-Con or AAV-DYRK1A-treated WT mice** |
| \| \| Table Analyzed \| Data 1 \| \| --- \| --- \| \|  \|  \| \| Column D \| Data Set-D \| \| vs. \| vs. \| \| Column C \| Data Set-C \| \|  \|  \| \| Unpaired t test \|  \| \| P value \| 0.1402 \| \| P value summary \| ns \| \| Significantly different (P < 0.05)? \| No \| \| One- or two-tailed P value? \| Two-tailed \| \| t, df \| t=1.564, df=14 \| \|  \| \| --- \| --- \| --- \| --- \| --- \| --- \| --- \| --- \| --- \| --- \| --- \| --- \| --- \| --- \| --- \| --- \| --- \| --- \| --- \| --- \| --- \| --- \| --- \| --- \| --- \| --- \| |
| **Figure 2A. DYRK1A mRNA level in AAV-shCon or AAV-shDYRK1A-treated 5xFAD mice** |
| \| Table Analyzed \| Figure 2A \| \| --- \| --- \| \|  \|  \| \| Column A \| Con \| \| vs. \| vs. \| \| Column B \| DYRK1A KD \| \|  \|  \| \| Unpaired t test \|  \| \| P value \| 0.0054 \| \| P value summary \| ** \| \| Significantly different (P < 0.05)? \| Yes \| \| One- or two-tailed P value? \| Two-tailed \| \| t, df \| t=3.334, df=13 \| |
| **Figure 2B. DYRK1A intensity in hippocampal CA1 of AAV-shCon or AAV-shDYRK1A-treated 5xFAD mice** |
| \| Table Analyzed \| CA1+DG \| \| --- \| --- \| \|  \|  \| \| Column A \| Con \| \| vs. \| vs. \| \| Column B \| DYRK1A KD \| \|  \|  \| \| Unpaired t test \|  \| \| P value \| <0.0001 \| \| P value summary \| **** \| \| Significantly different (P < 0.05)? \| Yes \| \| One- or two-tailed P value? \| Two-tailed \| \| t, df \| t=4.718, df=54 \| |
| **Figure 2B. DYRK1A intensity in hippocampal DG of AAV-shCon or AAV-shDYRK1A-treated 5xFAD mice** |
| \| Table Analyzed \| CA1+DG \| \| --- \| --- \| \|  \|  \| \| Column C \| CON \| \| vs. \| vs. \| \| Column D \| DYRK1A KD \| \|  \|  \| \| Unpaired t test \|  \| \| P value \| 0.0012 \| \| P value summary \| ** \| \| Significantly different (P < 0.05)? \| Yes \| \| One- or two-tailed P value? \| Two-tailed \| \| t, df \| t=3.421, df=53 \| |
| **Figure 2C. Y-maze, Spontaneous alternation in AAV-shCon or AAV-shDYRK1A-treated 5xFAD mice** |
| \| Table Analyzed \| Figure 2C \| \| --- \| --- \| \|  \|  \| \| Column A \| Con \| \| vs. \| vs. \| \| Column B \| DYRK1A KD \| \|  \|  \| \| Unpaired t test \|  \| \| P value \| 0.0002 \| \| P value summary \| *** \| \| Significantly different (P < 0.05)? \| Yes \| \| One- or two-tailed P value? \| Two-tailed \| \| t, df \| t=4.732, df=18 \| |
| **Figure 2C. Y- maze, Total arm entry in AAV-shCon or AAV-shDYRK1A-treated 5xFAD mice** |
| \| Table Analyzed \| Figure 2C \| \| --- \| --- \| \|  \|  \| \| Column C \| Con \| \| vs. \| vs. \| \| Column D \| DYRK1A KD \| \|  \|  \| \| Unpaired t test \|  \| \| P value \| 0.8894 \| \| P value summary \| ns \| \| Significantly different (P < 0.05)? \| No \| \| One- or two-tailed P value? \| Two-tailed \| \| t, df \| t=0.1411, df=18 \| |
| **Figure 2D. NOR Training in AAV-shCon or AAV-shDYRK1A-treated 5xFAD mice** |
| \| Table Analyzed \| Figure 2D \| \| --- \| --- \| \|  \|  \| \| Column A \| Con \| \| vs. \| vs. \| \| Column B \| DYRK1A KD \| \|  \|  \| \| Unpaired t test \|  \| \| P value \| 0.2284 \| \| P value summary \| ns \| \| Significantly different (P < 0.05)? \| No \| \| One- or two-tailed P value? \| Two-tailed \| \| t, df \| t=1.247, df=18 \| |
| **Figure 2D. NOR Test in AAV-shCon or AAV-shDYRK1A-treated 5xFAD mice** |
| \| Table Analyzed \| Figure 2D \| \| --- \| --- \| \|  \|  \| \| Column C \| Con \| \| vs. \| vs. \| \| Column D \| DYRK1A KD \| \|  \|  \| \| Unpaired t test \|  \| \| P value \| 0.0165 \| \| P value summary \| * \| \| Significantly different (P < 0.05)? \| Yes \| \| One- or two-tailed P value? \| Two-tailed \| \| t, df \| t=2.644, df=18 \| |
| **Figure 2E. p-CaMKIIα level in AAV-shCon or AAV-shDYRK1A-treated 5xFAD mice** |
| \| Table Analyzed \| pCaMKIIa, CaMKIIA \| \| --- \| --- \| \|  \|  \| \| Column B \| DYRK1A KD \| \| vs. \| vs. \| \| Column A \| Con \| \|  \|  \| \| Unpaired t test \|  \| \| P value \| 0.0370 \| \| P value summary \| * \| \| Significantly different (P < 0.05)? \| Yes \| \| One- or two-tailed P value? \| One-tailed \| \| t, df \| t=1.931, df=14 \| |
| **Figure 2E. CaMKIIα level in AAV-shCon or AAV-shDYRK1A-treated 5xFAD mice** |
| \| Table Analyzed \| pCaMKIIa, CaMKIIA \| \| --- \| --- \| \|  \|  \| \| Column D \| DYRK1A KD \| \| vs. \| vs. \| \| Column C \| CON \| \|  \|  \| \| Unpaired t test \|  \| \| P value \| 0.0800 \| \| P value summary \| ns \| \| Significantly different (P < 0.05)? \| No \| \| One- or two-tailed P value? \| Two-tailed \| \| t, df \| t=1.887, df=14 \| |
| **Figure 2E. p-CREB level in AAV-shCon or AAV-shDYRK1A-treated 5xFAD mice** |
| \| Table Analyzed \| pCREB,CREB \| \| --- \| --- \| \|  \|  \| \| Column B \| DYRK1A KD \| \| vs. \| vs. \| \| Column A \| Con \| \|  \|  \| \| Unpaired t test \|  \| \| P value \| 0.0203 \| \| P value summary \| * \| \| Significantly different (P < 0.05)? \| Yes \| \| One- or two-tailed P value? \| Two-tailed \| \| t, df \| t=2.616, df=14 \| |
| **Figure 2F. CREB level in AAV-shCon or AAV-shDYRK1A-treated 5xFAD mice** |
| \| Table Analyzed \| pCREB,CREB \| \| --- \| --- \| \|  \|  \| \| Column D \| DYRK1A KD \| \| vs. \| vs. \| \| Column C \| CON \| \|  \|  \| \| Unpaired t test \|  \| \| P value \| 0.3605 \| \| P value summary \| ns \| \| Significantly different (P < 0.05)? \| No \| \| One- or two-tailed P value? \| Two-tailed \| \| t, df \| t=0.9454, df=14 \| |
| **Figure 2G. DYRK1A mRNA level in AAV-shCon or AAV-shDYRK1A-treated 5xFAD mice** |
| \| Table Analyzed \| qPCR \| \| --- \| --- \| \|  \|  \| \| Column B \| DYRK1AKD \| \| vs. \| vs. \| \| Column A \| Con \| \|  \|  \| \| Unpaired t test \|  \| \| P value \| 0.0149 \| \| P value summary \| * \| \| Significantly different (P < 0.05)? \| Yes \| \| One- or two-tailed P value? \| Two-tailed \| \| t, df \| t=2.803, df=13 \| |
| **Figure 2H. Y-maze, Spontaneous alternation in AAV-shCon or AAV-shDYRK1A-treated 5xFAD mice** |
| \| Table Analyzed \| Y-Maze \|  \| \| --- \| --- \| --- \| \|  \|  \|  \| \| Column B \| DYRK1AKD \|  \| \| vs. \| vs. \|  \| \| Column A \| Con \|  \| \|  \|  \|  \| \| Unpaired t test \|  \|  \| \| P value \| 0.1492 \|  \| \| P value summary \| ns \|  \| \| Significantly different (P < 0.05)? \| No \|  \| \| One- or two-tailed P value? \| Two-tailed \|  \| \| t, df \| t=1.533, df=13 \|  \| |
| **Figure 2H. Y-maze, Total arm entry in AAV-shCon or AAV-shDYRK1A-treated 5xFAD mice** |
| \| Table Analyzed \| Y-Maze \| \| --- \| --- \| \|  \|  \| \| Column D \| DYRK1A KD \| \| vs. \| vs. \| \| Column C \| Con \| \|  \|  \| \| Unpaired t test \|  \| \| P value \| 0.3881 \| \| P value summary \| ns \| \| Significantly different (P < 0.05)? \| No \| \| One- or two-tailed P value? \| Two-tailed \| \| t, df \| t=0.8929, df=13 \| |
| **Figure 2I. NOR training in AAV-shCon or AAV-shDYRK1A-treated 5xFAD mice** |
| \| Table Analyzed \| NORT \| \| --- \| --- \| \|  \|  \| \| Column B \| DYRK1AKD \| \| vs. \| vs. \| \| Column A \| Con \| \|  \|  \| \| Unpaired t test \|  \| \| P value \| 0.4408 \| \| P value summary \| ns \| \| Significantly different (P < 0.05)? \| No \| \| One- or two-tailed P value? \| Two-tailed \| \| t, df \| t=0.7951, df=13 \| |
| **Figure 2I. NOR test in AAV-shCon or AAV-shDYRK1A-treated 5xFAD mice** |
| \| Table Analyzed \| NORT \| \| --- \| --- \| \|  \|  \| \| Column D \| DYRK1A KD \| \| vs. \| vs. \| \| Column C \| Con \| \|  \|  \| \| Unpaired t test \|  \| \| P value \| 0.0126 \| \| P value summary \| * \| \| Significantly different (P < 0.05)? \| Yes \| \| One- or two-tailed P value? \| Two-tailed \| \| t, df \| t=2.891, df=13 \| |
| **Figure 3A. il-1β mRNA level in AAV-shCon or AAV-shDYRK1A-treated 5xFAD mice** |
| \| Table Analyzed \| Figure 3A \| \| --- \| --- \| \|  \|  \| \| Column B \| DYRK1A KD \| \| vs. \| vs. \| \| Column A \| CON \| \|  \|  \| \| Unpaired t test \|  \| \| P value \| 0.0032 \| \| P value summary \| ** \| \| Significantly different (P < 0.05)? \| Yes \| \| One- or two-tailed P value? \| Two-tailed \| \| t, df \| t=3.544, df=14 \| |
| **Figure 3A. tnf-α mRNA level in AAV-shCon or AAV-shDYRK1A-treated 5xFAD mice** |
| \| Table Analyzed \| Figure 3A \| \| --- \| --- \| \|  \|  \| \| Column D \| DYRK1A KD \| \| vs. \| vs. \| \| Column C \| CON \| \|  \|  \| \| Unpaired t test \|  \| \| P value \| 0.0002 \| \| P value summary \| *** \| \| Significantly different (P < 0.05)? \| Yes \| \| One- or two-tailed P value? \| Two-tailed \| \| t, df \| t=5.034, df=14 \| |
| **Figure 3A. cox-2 mRNA level in AAV-shCon or AAV-shDYRK1A-treated 5xFAD mice** |
| \| Table Analyzed \| Figure 3A \| \| --- \| --- \| \|  \|  \| \| Column F \| DYRK1A KD \| \| vs. \| vs. \| \| Column E \| CON \| \|  \|  \| \| Unpaired t test \|  \| \| P value \| 0.0356 \| \| P value summary \| * \| \| Significantly different (P < 0.05)? \| Yes \| \| One- or two-tailed P value? \| Two-tailed \| \| t, df \| t=2.325, df=14 \| |
| **Figure 3A. il-6 mRNA level in AAV-shCon or AAV-shDYRK1A-treated 5xFAD mice** |
| \| Table Analyzed \| Figure 3A \| \| --- \| --- \| \|  \|  \| \| Column H \| DYRK1A KD \| \| vs. \| vs. \| \| Column G \| CON \| \|  \|  \| \| Unpaired t test \|  \| \| P value \| 0.0143 \| \| P value summary \| * \| \| Significantly different (P < 0.05)? \| Yes \| \| One- or two-tailed P value? \| Two-tailed \| \| t, df \| t=2.795, df=14 \| |
| **Figure 3B. Il-1β protein level in AAV-shCon or AAV-shDYRK1A-treated 5xFAD mice** |
| \| Table Analyzed \| Data 1 \| \| --- \| --- \| \|  \|  \| \| Column B \| Data Set-B \| \| vs. \| vs. \| \| Column A \| Data Set-A \| \|  \|  \| \| Unpaired t test \|  \| \| P value \| 0.0018 \| \| P value summary \| ** \| \| Significantly different (P < 0.05)? \| Yes \| \| One- or two-tailed P value? \| Two-tailed \| \| t, df \| t=3.913, df=13 \| |
| **Figure 3B. TNF-α protein level in AAV-shCon or AAV-shDYRK1A-treated 5xFAD mice** |
| \| Table Analyzed \| Data 1 \| \| --- \| --- \| \|  \|  \| \| Column D \| Data Set-D \| \| vs. \| vs. \| \| Column C \| Data Set-C \| \|  \|  \| \| Unpaired t test \|  \| \| P value \| 0.0003 \| \| P value summary \| *** \| \| Significantly different (P < 0.05)? \| Yes \| \| One- or two-tailed P value? \| Two-tailed \| \| t, df \| t=4.842, df=13 \| |
| **Figure 3B. COX-2 protein level in AAV-shCon or AAV-shDYRK1A-treated 5xFAD mice** |
| \| Table Analyzed \| Data 1 \| \| --- \| --- \| \|  \|  \| \| Column F \| Data Set-F \| \| vs. \| vs. \| \| Column E \| Data Set-E \| \|  \|  \| \| Unpaired t test \|  \| \| P value \| 0.0059 \| \| P value summary \| ** \| \| Significantly different (P < 0.05)? \| Yes \| \| One- or two-tailed P value? \| Two-tailed \| \| t, df \| t=3.283, df=13 \| |
| **Figure 3B. IL-6 protein level in AAV-shCon or AAV-shDYRK1A-treated 5xFAD mice** |
| \| Table Analyzed \| Data 1 \| \| --- \| --- \| \|  \|  \| \| Column H \| Data Set-H \| \| vs. \| vs. \| \| Column G \| Data Set-G \| \|  \|  \| \| Unpaired t test \|  \| \| P value \| 0.0012 \| \| P value summary \| ** \| \| Significantly different (P < 0.05)? \| Yes \| \| One- or two-tailed P value? \| Two-tailed \| \| t, df \| t=4.126, df=13 \| |
| **Figure 3C. nlrp3 mRNA level in AAV-shCon or AAV-shDYRK1A-treated 5xFAD mice** |
| \| Table Analyzed \| Figure 3B \| \| --- \| --- \| \|  \|  \| \| Column B \| DYRK1A KD \| \| vs. \| vs. \| \| Column A \| CON \| \|  \|  \| \| Unpaired t test \|  \| \| P value \| 0.0017 \| \| P value summary \| ** \| \| Significantly different (P < 0.05)? \| Yes \| \| One- or two-tailed P value? \| Two-tailed \| \| t, df \| t=3.872, df=14 \| |
| **Figure 3C. sod2 mRNA level in AAV-shCon or AAV-shDYRK1A-treated 5xFAD mice** |
| \| Table Analyzed \| Figure 3B \| \| --- \| --- \| \|  \|  \| \| Column D \| DYRK1A KD \| \| vs. \| vs. \| \| Column C \| CON \| \|  \|  \| \| Unpaired t test \|  \| \| P value \| 0.2110 \| \| P value summary \| ns \| \| Significantly different (P < 0.05)? \| No \| \| One- or two-tailed P value? \| Two-tailed \| \| t, df \| t=1.311, df=14 \| |
| **Figure 3D. gfap mRNA level in AAV-shCon or AAV-shDYRK1A-treated 5xFAD mice** |
| \| Table Analyzed \| Figure 3C \| \| --- \| --- \| \|  \|  \| \| Column B \| DYRK1A KD \| \| vs. \| vs. \| \| Column A \| CON \| \|  \|  \| \| Unpaired t test \|  \| \| P value \| 0.0017 \| \| P value summary \| ** \| \| Significantly different (P < 0.05)? \| Yes \| \| One- or two-tailed P value? \| Two-tailed \| \| t, df \| t=3.872, df=14 \| |
| **Figure 3D. gbp2 mRNA level in AAV-shCon or AAV-shDYRK1A-treated 5xFAD mice** |
| \| Table Analyzed \| Figure 3D \| \| --- \| --- \| \|  \|  \| \| Column B \| DYRK1A KD \| \| vs. \| vs. \| \| Column A \| CON \| \|  \|  \| \| Unpaired t test \|  \| \| P value \| 0.0019 \| \| P value summary \| ** \| \| Significantly different (P < 0.05)? \| Yes \| \| One- or two-tailed P value? \| Two-tailed \| \| t, df \| t=3.826, df=14 \| |
| **Figure 3D. cxcl10 mRNA level in AAV-shCon or AAV-shDYRK1A-treated 5xFAD mice** |
| \| Table Analyzed \| Figure 3E \| \| --- \| --- \| \|  \|  \| \| Column B \| DYRK1A KD \| \| vs. \| vs. \| \| Column A \| CON \| \|  \|  \| \| Unpaired t test \|  \| \| P value \| 0.0008 \| \| P value summary \| *** \| \| Significantly different (P < 0.05)? \| Yes \| \| One- or two-tailed P value? \| Two-tailed \| \| t, df \| t=4.284, df=14 \| |
| **Figure 3E. dst mRNA level in AAV-shCon or AAV-shDYRK1A-treated 5xFAD mice** |
| \| Table Analyzed \|  \| Figure 3F \| \| --- \| --- \| --- \| \|  \|  \|  \| \| Column D \|  \| DYRK1A KD \| \| vs. \|  \| vs. \| \| Column C \|  \| CON \| \|  \|  \|  \| \| Unpaired t test \|  \|  \| \| P value \|  \| 0.0062 \| \| P value summary \|  \| ** \| \| Significantly different (P < 0.05)? \|  \| Yes \| \| One- or two-tailed P value? \|  \| Two-tailed \| \| t, df \|  \| t=3.216, df=14 \| |
| **Figure 3E. nestin mRNA level in AAV-shCon or AAV-shDYRK1A-treated 5xFAD mice** |
| \| Table Analyzed \| Figure 3F \| \| --- \| --- \| \|  \|  \| \| Column B \| DYRK1A KD \| \| vs. \| vs. \| \| Column A \| CON \| \|  \|  \| \| Unpaired t test \|  \| \| P value \| 0.1693 \| \| P value summary \| ns \| \| Significantly different (P < 0.05)? \| No \| \| One- or two-tailed P value? \| Two-tailed \| \| t, df \| t=1.449, df=14 \| |
| **Figure 3F. iba-1 mRNA level in AAV-shCon or AAV-shDYRK1A-treated 5xFAD mice** |
| \| Table Analyzed \| Figure 3G \| \| --- \| --- \| \|  \|  \| \| Column B \| DYRK1A KD \| \| vs. \| vs. \| \| Column A \| CON \| \|  \|  \| \| Unpaired t test \|  \| \| P value \| 0.0003 \| \| P value summary \| *** \| \| Significantly different (P < 0.05)? \| Yes \| \| One- or two-tailed P value? \| Two-tailed \| \| t, df \| t=4.766, df=14 \| |
| **Figure 3G. itgax mRNA level in AAV-shCon or AAV-shDYRK1A-treated 5xFAD mice** |
| \| Table Analyzed \| Figure 3H \| \| --- \| --- \| \|  \|  \| \| Column B \| DYRK1A KD \| \| vs. \| vs. \| \| Column A \| CON \| \|  \|  \| \| Unpaired t test \|  \| \| P value \| 0.0034 \| \| P value summary \| ** \| \| Significantly different (P < 0.05)? \| Yes \| \| One- or two-tailed P value? \| Two-tailed \| \| t, df \| t=3.521, df=14 \| |
| **Figure 3G. trem2 mRNA level in AAV-shCon or AAV-shDYRK1A-treated 5xFAD mice** |
| \| Table Analyzed \| Figure 3H \| \| --- \| --- \| \|  \|  \| \| Column F \| DYRK1A KD \| \| vs. \| vs. \| \| Column E \| CON \| \|  \|  \| \| Unpaired t test \|  \| \| P value \| 0.0179 \| \| P value summary \| * \| \| Significantly different (P < 0.05)? \| Yes \| \| One- or two-tailed P value? \| Two-tailed \| \| t, df \| t=2.681, df=14 \| |
| **Figure 3G. clec7a mRNA level in AAV-shCon or AAV-shDYRK1A-treated 5xFAD mice** |
| \| Table Analyzed \| Figure 3H \| \| --- \| --- \| \|  \|  \| \| Column D \| DYRK1A KD \| \| vs. \| vs. \| \| Column C \| CON \| \|  \|  \| \| Unpaired t test \|  \| \| P value \| 0.0526 \| \| P value summary \| ns \| \| Significantly different (P < 0.05)? \| No \| \| One- or two-tailed P value? \| Two-tailed \| \| t, df \| t=2.117, df=14 \| |
| **Figure 3H. cr3 mRNA level in AAV-shCon or AAV-shDYRK1A-treated 5xFAD mice** |
| \| Table Analyzed \| Figure 3I \| \| --- \| --- \| \|  \|  \| \| Column B \| DYRK1A KD \| \| vs. \| vs. \| \| Column A \| CON \| \|  \|  \| \| Unpaired t test \|  \| \| P value \| <0.0001 \| \| P value summary \| **** \| \| Significantly different (P < 0.05)? \| Yes \| \| One- or two-tailed P value? \| Two-tailed \| \| t, df \| t=7.312, df=14 \| |
| **Figure 3H. c1qa mRNA level in AAV-shCon or AAV-shDYRK1A-treated 5xFAD mice** |
| \| Table Analyzed \| Figure 3I \| \| --- \| --- \| \|  \|  \| \| Column D \| DYRK1A KD \| \| vs. \| vs. \| \| Column C \| CON \| \|  \|  \| \| Unpaired t test \|  \| \| P value \| 0.0010 \| \| P value summary \| *** \| \| Significantly different (P < 0.05)? \| Yes \| \| One- or two-tailed P value? \| Two-tailed \| \| t, df \| t=4.162, df=14 \| |
| **Figure 4A. il-1β mRNA level in AAV-shCon or AAV-shDYRK1A-treated 5xFAD mice** |
| \| Table Analyzed \| Data 1 \| \| --- \| --- \| \|  \|  \| \| Column B \| Data Set-B \| \| vs. \| vs. \| \| Column A \| Data Set-A \| \|  \|  \| \| Unpaired t test \|  \| \| P value \| 0.0240 \| \| P value summary \| * \| \| Significantly different (P < 0.05)? \| Yes \| \| One- or two-tailed P value? \| Two-tailed \| \| t, df \| t=2.554, df=13 \| |
| **Figure 4A. tnf-α mRNA level in AAV-shCon or AAV-shDYRK1A-treated 5xFAD mice** |
| \| Table Analyzed \| Data 1 \| \| --- \| --- \| \|  \|  \| \| Column D \| Data Set-D \| \| vs. \| vs. \| \| Column C \| Data Set-C \| \|  \|  \| \| Unpaired t test \|  \| \| P value \| 0.0422 \| \| P value summary \| * \| \| Significantly different (P < 0.05)? \| Yes \| \| One- or two-tailed P value? \| Two-tailed \| \| t, df \| t=2.253, df=13 \| |
| **Figure 4A. cox-2 mRNA level in AAV-shCon or AAV-shDYRK1A-treated 5xFAD mice** |
| \| Table Analyzed \| Data 1 \| \| --- \| --- \| \|  \|  \| \| Column F \| Data Set-F \| \| vs. \| vs. \| \| Column E \| Data Set-E \| \|  \|  \| \| Unpaired t test \|  \| \| P value \| 0.5655 \| \| P value summary \| ns \| \| Significantly different (P < 0.05)? \| No \| \| One- or two-tailed P value? \| Two-tailed \| \| t, df \| t=0.5897, df=13 \| |
| **Figure 4A. il-6 mRNA level in AAV-shCon or AAV-shDYRK1A-treated 5xFAD mice** |
| \| Table Analyzed \| Data 1 \| \| --- \| --- \| \|  \|  \| \| Column H \| Data Set-H \| \| vs. \| vs. \| \| Column G \| Data Set-G \| \|  \|  \| \| Unpaired t test \|  \| \| P value \| 0.1390 \| \| P value summary \| ns \| \| Significantly different (P < 0.05)? \| No \| \| One- or two-tailed P value? \| Two-tailed \| \| t, df \| t=1.585, df=12 \| |
| **Figure 4B. nlrp3 mRNA level in AAV-shCon or AAV-shDYRK1A-treated 5xFAD mice** |
| \| Table Analyzed \| Data 1 \| \| --- \| --- \| \|  \|  \| \| Column B \| Data Set-B \| \| vs. \| vs. \| \| Column A \| Data Set-A \| \|  \|  \| \| Unpaired t test \|  \| \| P value \| 0.0050 \| \| P value summary \| ** \| \| Significantly different (P < 0.05)? \| Yes \| \| One- or two-tailed P value? \| Two-tailed \| \| t, df \| t=3.373, df=13 \| |
| **Figure 4B. sod2 mRNA level in AAV-shCon or AAV-shDYRK1A-treated 5xFAD mice** |
| \| Table Analyzed \| Data 1 \| \| --- \| --- \| \|  \|  \| \| Column D \| Data Set-D \| \| vs. \| vs. \| \| Column C \| Data Set-C \| \|  \|  \| \| Unpaired t test \|  \| \| P value \| 0.1140 \| \| P value summary \| ns \| \| Significantly different (P < 0.05)? \| No \| \| One- or two-tailed P value? \| Two-tailed \| \| t, df \| t=1.694, df=13 \| |
| **Figure 4C. gfap mRNA level in AAV-shCon or AAV-shDYRK1A-treated 5xFAD mice** |
| \| Table Analyzed \| Data 1 \| \| --- \| --- \| \|  \|  \| \| Column B \| Data Set-B \| \| vs. \| vs. \| \| Column A \| Data Set-A \| \|  \|  \| \| Unpaired t test \|  \| \| P value \| 0.0374 \| \| P value summary \| * \| \| Significantly different (P < 0.05)? \| Yes \| \| One- or two-tailed P value? \| Two-tailed \| \| t, df \| t=2.318, df=13 \| |
| **Figure 4D. gbp2 mRNA level in AAV-shCon or AAV-shDYRK1A-treated 5xFAD mice** |
| \| Table Analyzed \| Data 1 \| \| --- \| --- \| \|  \|  \| \| Column B \| Data Set-B \| \| vs. \| vs. \| \| Column A \| Data Set-A \| \|  \|  \| \| Unpaired t test \|  \| \| P value \| 0.0181 \| \| P value summary \| * \| \| Significantly different (P < 0.05)? \| Yes \| \| One- or two-tailed P value? \| Two-tailed \| \| t, df \| t=2.703, df=13 \| |
| **Figure 4E. cxcl10 mRNA level in AAV-shCon or AAV-shDYRK1A-treated 5xFAD mice** |
| \| Table Analyzed \| Data 1 \| \| --- \| --- \| \|  \|  \| \| Column B \| Data Set-B \| \| vs. \| vs. \| \| Column A \| Data Set-A \| \|  \|  \| \| Unpaired t test \|  \| \| P value \| 0.0108 \| \| P value summary \| * \| \| Significantly different (P < 0.05)? \| Yes \| \| One- or two-tailed P value? \| Two-tailed \| \| t, df \| t=2.972, df=13 \| |
| **Figure 4F. dst mRNA level in AAV-shCon or AAV-shDYRK1A-treated 5xFAD mice** |
| \| Table Analyzed \| Data 1 \| \| --- \| --- \| \|  \|  \| \| Column B \| Data Set-B \| \| vs. \| vs. \| \| Column A \| Data Set-A \| \|  \|  \| \| Unpaired t test \|  \| \| P value \| 0.7618 \| \| P value summary \| ns \| \| Significantly different (P < 0.05)? \| No \| \| One- or two-tailed P value? \| Two-tailed \| \| t, df \| t=0.3095, df=13 \| |
| **Figure 4G. nestin mRNA level in AAV-shCon or AAV-shDYRK1A-treated 5xFAD mice** |
| \| Table Analyzed \| Data 1 \| \| --- \| --- \| \|  \|  \| \| Column B \| Data Set-B \| \| vs. \| vs. \| \| Column A \| Data Set-A \| \|  \|  \| \| Unpaired t test \|  \| \| P value \| 0.1070 \| \| P value summary \| ns \| \| Significantly different (P < 0.05)? \| No \| \| One- or two-tailed P value? \| Two-tailed \| \| t, df \| t=1.732, df=13 \| |
| **Figure 4H. iba-1 mRNA level in AAV-shCon or AAV-shDYRK1A-treated 5xFAD mice** |
| \| Table Analyzed \| Data 1 \| \| --- \| --- \| \|  \|  \| \| Column B \| Data Set-B \| \| vs. \| vs. \| \| Column A \| Data Set-A \| \|  \|  \| \| Unpaired t test \|  \| \| P value \| 0.0020 \| \| P value summary \| ** \| \| Significantly different (P < 0.05)? \| Yes \| \| One- or two-tailed P value? \| Two-tailed \| \| t, df \| t=3.847, df=13 \| |
| **Figure 4I. itgax mRNA level in AAV-shCon or AAV-shDYRK1A-treated 5xFAD mice** |
| \| Table Analyzed \| Data 1 \| \| --- \| --- \| \|  \|  \| \| Column B \| Data Set-B \| \| vs. \| vs. \| \| Column A \| Data Set-A \| \|  \|  \| \| Unpaired t test \|  \| \| P value \| 0.0387 \| \| P value summary \| * \| \| Significantly different (P < 0.05)? \| Yes \| \| One- or two-tailed P value? \| Two-tailed \| \| t, df \| t=2.300, df=13 \| |
| **Figure 4I. trem2 mRNA level in AAV-shCon or AAV-shDYRK1A-treated 5xFAD mice** |
| \| Table Analyzed \| Data 1 \| \| --- \| --- \| \|  \|  \| \| Column D \| Data Set-D \| \| vs. \| vs. \| \| Column C \| Data Set-C \| \|  \|  \| \| Unpaired t test \|  \| \| P value \| 0.1780 \| \| P value summary \| ns \| \| Significantly different (P < 0.05)? \| No \| \| One- or two-tailed P value? \| Two-tailed \| \| t, df \| t=1.424, df=13 \| |
| **Figure 4I. clec7a mRNA level in AAV-shCon or AAV-shDYRK1A-treated 5xFAD mice** |
| \| Table Analyzed \| Data 1 \| \| --- \| --- \| \|  \|  \| \| Column F \| Data Set-F \| \| vs. \| vs. \| \| Column E \| Data Set-E \| \|  \|  \| \| Unpaired t test \|  \| \| P value \| 0.0004 \| \| P value summary \| *** \| \| Significantly different (P < 0.05)? \| Yes \| \| One- or two-tailed P value? \| Two-tailed \| \| t, df \| t=4.665, df=13 \| |
| **Figure 4J. cr3 mRNA level in AAV-shCon or AAV-shDYRK1A-treated 5xFAD mice** |
| \| Table Analyzed \| Data 1 \| \| --- \| --- \| \|  \|  \| \| Column B \| Data Set-B \| \| vs. \| vs. \| \| Column A \| Data Set-A \| \|  \|  \| \| Unpaired t test \|  \| \| P value \| 0.0052 \| \| P value summary \| ** \| \| Significantly different (P < 0.05)? \| Yes \| \| One- or two-tailed P value? \| Two-tailed \| \| t, df \| t=3.352, df=13 \| |
| **Figure 4J. c1qa mRNA level in AAV-shCon or AAV-shDYRK1A-treated 5xFAD mice** |
| \| Table Analyzed \| Data 1 \| \| --- \| --- \| \|  \|  \| \| Column D \| Data Set-D \| \| vs. \| vs. \| \| Column C \| Data Set-C \| \|  \|  \| \| Unpaired t test \|  \| \| P value \| 0.0039 \| \| P value summary \| ** \| \| Significantly different (P < 0.05)? \| Yes \| \| One- or two-tailed P value? \| Two-tailed \| \| t, df \| t=3.507, df=13 \| |
| **Figure 5A. DYRK1A mRNA level in AAV-Con or AAV-DYRK1A-treated 5xFAD mice** |
| \| Table Analyzed \| Figure 4A \| \| --- \| --- \| \|  \|  \| \| Column B \| DYRK1A OE \| \| vs. \| vs. \| \| Column A \| CON \| \|  \|  \| \| Unpaired t test \|  \| \| P value \| 0.0453 \| \| P value summary \| * \| \| Significantly different (P < 0.05)? \| Yes \| \| One- or two-tailed P value? \| Two-tailed \| \| t, df \| t=2.287, df=10 \| |
| **Figure 5B. il-1β mRNA level in AAV-Con or AAV-DYRK1A-treated 5xFAD mice** |
| \| Table Analyzed \| Figure 4B \| \| --- \| --- \| \|  \|  \| \| Column B \| DYRK1A OE \| \| vs. \| vs. \| \| Column A \| CON \| \|  \|  \| \| Unpaired t test \|  \| \| P value \| 0.0014 \| \| P value summary \| ** \| \| Significantly different (P < 0.05)? \| Yes \| \| One- or two-tailed P value? \| Two-tailed \| \| t, df \| t=4.363, df=10 \| |
| **Figure 5B. tnf-a mRNA level in AAV-Con or AAV-DYRK1A-treated 5xFAD mice** |
| \| Table Analyzed \| Figure 4B \| \| --- \| --- \| \|  \|  \| \| Column D \| DYRK1A OE \| \| vs. \| vs. \| \| Column C \| CON \| \|  \|  \| \| Unpaired t test \|  \| \| P value \| 0.1262 \| \| P value summary \| ns \| \| Significantly different (P < 0.05)? \| No \| \| One- or two-tailed P value? \| Two-tailed \| \| t, df \| t=1.668, df=10 \| |
| **Figure 5B. cox-2 mRNA level in AAV-Con or AAV-DYRK1A-treated 5xFAD mice** |
| \| Table Analyzed \| Figure 4B \| \| --- \| --- \| \|  \|  \| \| Column F \| DYRK1A OE \| \| vs. \| vs. \| \| Column E \| CON \| \|  \|  \| \| Unpaired t test \|  \| \| P value \| 0.3135 \| \| P value summary \| ns \| \| Significantly different (P < 0.05)? \| No \| \| One- or two-tailed P value? \| Two-tailed \| \| t, df \| t=1.061, df=10 \| |
| **Figure 5B. il-6 mRNA level in AAV-Con or AAV-DYRK1A-treated 5xFAD mice** |
| \| Table Analyzed \| Figure 4B \| \| --- \| --- \| \|  \|  \| \| Column H \| DYRK1A OE \| \| vs. \| vs. \| \| Column G \| CON \| \|  \|  \| \| Unpaired t test \|  \| \| P value \| 0.5999 \| \| P value summary \| ns \| \| Significantly different (P < 0.05)? \| No \| \| One- or two-tailed P value? \| Two-tailed \| \| t, df \| t=0.5416, df=10 \| |
| **Figure 5C nlrp3 mRNA level in AAV-Con or AAV-DYRK1A-treated 5xFAD mice** |
| \| Table Analyzed \| Figure 4C \| \| --- \| --- \| \|  \|  \| \| Column B \| DYRK1A OE \| \| vs. \| vs. \| \| Column A \| CON \| \|  \|  \| \| Unpaired t test \|  \| \| P value \| 0.0048 \| \| P value summary \| ** \| \| Significantly different (P < 0.05)? \| Yes \| \| One- or two-tailed P value? \| Two-tailed \| \| t, df \| t=3.608, df=10 \| |
| **Figure5C. sod2 mRNA level in AAV-Con or AAV-DYRK1A-treated 5xFAD mice** |
| \| Table Analyzed \| Figure 4C \| \| --- \| --- \| \|  \|  \| \| Column D \| DYRK1A OE \| \| vs. \| vs. \| \| Column C \| CON \| \|  \|  \| \| Unpaired t test \|  \| \| P value \| 0.0355 \| \| P value summary \| * \| \| Significantly different (P < 0.05)? \| Yes \| \| One- or two-tailed P value? \| Two-tailed \| \| t, df \| t=2.428, df=10 \| |
| **Figure 5D. gfap mRNA level in AAV-Con or AAV-DYRK1A-treated 5xFAD mice** |
| \| Table Analyzed \| Figure 4D \| \| --- \| --- \| \|  \|  \| \| Column B \| DYRK1A OE \| \| vs. \| vs. \| \| Column A \| CON \| \|  \|  \| \| Unpaired t test \|  \| \| P value \| 0.2394 \| \| P value summary \| ns \| \| Significantly different (P < 0.05)? \| No \| \| One- or two-tailed P value? \| Two-tailed \| \| t, df \| t=1.251, df=10 \| |
| **Figure 5E. bp2 mRNA level in AAV-Con or AAV-DYRK1A-treated 5xFAD mice** |
| \| Table Analyzed \| Figure 4E \| \| --- \| --- \| \|  \|  \| \| Column B \| DYRK1A OE \| \| vs. \| vs. \| \| Column A \| CON \| \|  \|  \| \| Unpaired t test \|  \| \| P value \| 0.0126 \| \| P value summary \| * \| \| Significantly different (P < 0.05)? \| Yes \| \| One- or two-tailed P value? \| Two-tailed \| \| t, df \| t=3.033, df=10 \| |
| **Figure 5F. cxcl10 mRNA level in AAV-Con or AAV-DYRK1A-treated 5xFAD mice** |
| \| Table Analyzed \| Figure 4F \| \| --- \| --- \| \|  \|  \| \| Column B \| DYRK1A OE \| \| vs. \| vs. \| \| Column A \| CON \| \|  \|  \| \| Unpaired t test \|  \| \| P value \| 0.0297 \| \| P value summary \| * \| \| Significantly different (P < 0.05)? \| Yes \| \| One- or two-tailed P value? \| Two-tailed \| \| t, df \| t=2.534, df=10 \| |
| **Figure 5G. dst mRNA level in AAV-Con or AAV-DYRK1A-treated 5xFAD mice** |
| \| Table Analyzed \| Figure 4G \| \| --- \| --- \| \|  \|  \| \| Column D \| DYRK1A OE \| \| vs. \| vs. \| \| Column C \| CON \| \|  \|  \| \| Unpaired t test \|  \| \| P value \| 0.0323 \| \| P value summary \| * \| \| Significantly different (P < 0.05)? \| Yes \| \| One- or two-tailed P value? \| Two-tailed \| \| t, df \| t=2.528, df=9 \| |
| **Figure 5H. nestin mRNA level in AAV-Con or AAV-DYRK1A-treated 5xFAD mice** |
| \| Table Analyzed \| Figure 4G \| \| --- \| --- \| \|  \|  \| \| Column B \| DYRK1A OE \| \| vs. \| vs. \| \| Column A \| CON \| \|  \|  \| \| Unpaired t test \|  \| \| P value \| 0.0481 \| \| P value summary \| * \| \| Significantly different (P < 0.05)? \| Yes \| \| One- or two-tailed P value? \| Two-tailed \| \| t, df \| t=2.251, df=10 \| |
| **Figure 5I. iba-1 mRNA level in AAV-Con or AAV-DYRK1A-treated 5xFAD mice** |
| \| Table Analyzed \| Figure 4H \| \| --- \| --- \| \|  \|  \| \| Column B \| DYRK1A OE \| \| vs. \| vs. \| \| Column A \| CON \| \|  \|  \| \| Unpaired t test \|  \| \| P value \| 0.0055 \| \| P value summary \| ** \| \| Significantly different (P < 0.05)? \| Yes \| \| One- or two-tailed P value? \| Two-tailed \| \| t, df \| t=3.520, df=10 \| |
| **Figure 5J. itgax mRNA level in AAV-Con or AAV-DYRK1A-treated 5xFAD mice** |
| \| Table Analyzed \| Figure 4I \| \| --- \| --- \| \|  \|  \| \| Column B \| DYRK1A OE \| \| vs. \| vs. \| \| Column A \| CON \| \|  \|  \| \| Unpaired t test \|  \| \| P value \| 0.1318 \| \| P value summary \| ns \| \| Significantly different (P < 0.05)? \| No \| \| One- or two-tailed P value? \| Two-tailed \| \| t, df \| t=1.641, df=10 \| |
| **Figure 5J. trem2 mRNA level in AAV-Con or AAV-DYRK1A-treated 5xFAD mice** |
| \| Table Analyzed \| Figure 4I \| \| --- \| --- \| \|  \|  \| \| Column F \| DYRK1A OE \| \| vs. \| vs. \| \| Column E \| CON \| \|  \|  \| \| Unpaired t test \|  \| \| P value \| 0.4998 \| \| P value summary \| ns \| \| Significantly different (P < 0.05)? \| No \| \| One- or two-tailed P value? \| Two-tailed \| \| t, df \| t=0.7002, df=10 \| |
| **Figure 5J. clec7a mRNA level in AAV-Con or AAV-DYRK1A-treated 5xFAD mice** |
| \| Table Analyzed \| Figure 4I \| \| --- \| --- \| \|  \|  \| \| Column D \| DYRK1A OE \| \| vs. \| vs. \| \| Column C \| CON \| \|  \|  \| \| Unpaired t test \|  \| \| P value \| 0.1187 \| \| P value summary \| ns \| \| Significantly different (P < 0.05)? \| No \| \| One- or two-tailed P value? \| Two-tailed \| \| t, df \| t=1.706, df=10 \| |
| **Figure 5K. cr3 mRNA level in AAV-Con or AAV-DYRK1A-treated 5xFAD mice** |
| \| Table Analyzed \| Figure 4J \| \| --- \| --- \| \|  \|  \| \| Column B \| DYRK1A OE \| \| vs. \| vs. \| \| Column A \| CON \| \|  \|  \| \| Unpaired t test \|  \| \| P value \| 0.0339 \| \| P value summary \| * \| \| Significantly different (P < 0.05)? \| Yes \| \| One- or two-tailed P value? \| Two-tailed \| \| t, df \| t=2.456, df=10 \| |
| **Figure 5L. c1qa mRNA level in AAV-Con or AAV-DYRK1A-treated 5xFAD mice** |
| \| Table Analyzed \| Figure 4J \| \| --- \| --- \| \|  \|  \| \| Column D \| DYRK1A OE \| \| vs. \| vs. \| \| Column C \| CON \| \|  \|  \| \| Unpaired t test \|  \| \| P value \| 0.3475 \| \| P value summary \| ns \| \| Significantly different (P < 0.05)? \| No \| \| One- or two-tailed P value? \| Two-tailed \| \| t, df \| t=0.9858, df=10 \| |
| **Figure 6A. HO-1 level in AAV-shCon or AAV-shDYRK1A-treated 5xFAD mice** |
| \| Table Analyzed \| HO-1 \| \| --- \| --- \| \|  \|  \| \| Column B \| Data Set-B \| \| vs. \| vs. \| \| Column A \| Data Set-A \| \|  \|  \| \| Mann Whitney test \|  \| \| P value \| 0.0262 \| \| Exact or approximate P value? \| Exact \| \| P value summary \| * \| \| Significantly different (P < 0.05)? \| Yes \| \| One- or two-tailed P value? \| Two-tailed \| \| Sum of  ranks in column A,B \| 35 , 70 \| \| Mann-Whitney U \| 7 \| |
| **Figure 6B. p-AKT level in AAV-shCon or AAV-shDYRK1A-treated 5xFAD mice** |
| \| Table Analyzed \| pAKT \| \| --- \| --- \| \|  \|  \| \| Column B \| DYRK1A KD \| \| vs. \| vs. \| \| Column A \| Con \| \|  \|  \| \| Unpaired t test \|  \| \| P value \| 0.3061 \| \| P value summary \| ns \| \| Significantly different (P < 0.05)? \| No \| \| One- or two-tailed P value? \| Two-tailed \| \| t, df \| t=1.069, df=12 \| |
| **Figure 6B. AKT level in AAV-shCon or AAV-shDYRK1A-treated 5xFAD mice** |
| \| Table Analyzed \| AKT \| \| --- \| --- \| \|  \|  \| \| Column D \| DYRK1A KD \| \| vs. \| vs. \| \| Column C \| CON \| \|  \|  \| \| Unpaired t test \|  \| \| P value \| 0.1770 \| \| P value summary \| ns \| \| Significantly different (P < 0.05)? \| No \| \| One- or two-tailed P value? \| Two-tailed \| \| t, df \| t=0.9858, df=10 \| |
| **Figure 6C. p-STAT3 level in AAV-shCon or AAV-shDYRK1A-treated 5xFAD mice** |
| \| Table Analyzed \| pSTAT3 \| \| --- \| --- \| \|  \|  \| \| Column B \| DYRK1A KD \| \| vs. \| vs. \| \| Column A \| Con \| \|  \|  \| \| Unpaired t test \|  \| \| P value \| 0.1061 \| \| P value summary \| ns \| \| Significantly different (P < 0.05)? \| No \| \| One- or two-tailed P value? \| Two-tailed \| \| t, df \| t=1.747, df=12 \| |
| **Figure 6C. STAT3 level in AAV-shCon or AAV-shDYRK1A-treated 5xFAD mice** |
| \| Table Analyzed \| STAT3 \| \| --- \| --- \| \|  \|  \| \| Column D \| DYRK1A KD \| \| vs. \| vs. \| \| Column C \| CON \| \|  \|  \| \| Unpaired t test \|  \| \| P value \| 0.3776 \| \| P value summary \| ns \| \| Significantly different (P < 0.05)? \| No \| \| One- or two-tailed P value? \| Two-tailed \| \| t, df \| t=0.9162, df=12 \| |
| **Figure 6D. p-NF-κB level in AAV-shCon or AAV-shDYRK1A-treated 5xFAD mice** |
| \| Table Analyzed \| pNFKB \| \| --- \| --- \| \|  \|  \| \| Column B \| DYRK1A KD \| \| vs. \| vs. \| \| Column A \| Con \| \|  \|  \| \| Unpaired t test \|  \| \| P value \| 0.2904 \| \| P value summary \| ns \| \| Significantly different (P < 0.05)? \| No \| \| One- or two-tailed P value? \| Two-tailed \| \| t, df \| t=1.106, df=12 \| |
| **Figure 6D. NF-κB level in AAV-shCon or AAV-shDYRK1A-treated 5xFAD mice** |
| \| Table Analyzed \| NFKB \| \| --- \| --- \| \|  \|  \| \| Column D \| DYRK1A KD \| \| vs. \| vs. \| \| Column C \| CON \| \|  \|  \| \| Unpaired t test \|  \| \| P value \| 0.9479 \| \| P value summary \| ns \| \| Significantly different (P < 0.05)? \| No \| \| One- or two-tailed P value? \| Two-tailed \| \| t, df \| t=0.06674, df=12 \| |
| **Figure 6E. ROS level in AAV-Con or AAV-DYRK1A-treated 5xFAD mice** |
| \| Table Analyzed \| ROS \| \| --- \| --- \| \|  \|  \| \| Column B \| Data Set-B \| \| vs. \| vs. \| \| Column A \| Data Set-A \| \|  \|  \| \| Lognormal t test \|  \| \| P value \| 0.6993 \| \| P value summary \| ns \| \| Significantly different (P < 0.05)? \| No \| \| One- or two-tailed P value? \| Two-tailed \| \| t, df \| t=0.3950, df=13 \| |
| **Figure 6F. p-AKT level in AAV-Con or AAV-DYRK1A-treated 5xFAD mice** |
| \| Table Analyzed \| pAKT \| \| --- \| --- \| \|  \|  \| \| Column B \| DYRK1A KD \| \| vs. \| vs. \| \| Column A \| Con \| \|  \|  \| \| Unpaired t test \|  \| \| P value \| 0.3341 \| \| P value summary \| ns \| \| Significantly different (P < 0.05)? \| No \| \| One- or two-tailed P value? \| Two-tailed \| \| t, df \| t=1.006, df=12 \| |
| **Figure 6F. AKT level in AAV-Con or AAV-DYRK1A-treated 5xFAD mice** |
| \| Table Analyzed \| pAKT, AKT \| \| --- \| --- \| \|  \|  \| \| Column C \| CON \| \| vs. \| vs. \| \| Column D \| DYRK1A KD \| \|  \|  \| \| Kolmogorov-Smirnov test \|  \| \| P value \| 0.2121 \| \| Exact or approximate P value? \| Exact \| \| P value summary \| ns \| \| Significantly different (P < 0.05)? \| No \| \| Kolmogorov-Smirnov D \| 0.5714 \| |
| **Figure 6G. p-STAT3 level in AAV-Con or AAV-DYRK1A-treated 5xFAD mice** |
| \| \| Table Analyzed \| pSTAT3 \| \| --- \| --- \| \|  \|  \| \| Column B \| DYRK1A KD \| \| vs. \| vs. \| \| Column A \| Con \| \|  \|  \| \| Lognormal Welch’s t test \|  \| \| P value \| 0.0417 \| \| P value summary \| * \| \| Significantly different (P < 0.05)? \| Yes \| \| One- or two-tailed P value? \| Two-tailed \| \| Welch-corrected  t, df \| t=2.444, df=7.627 \| \|  \| \| --- \| --- \| --- \| --- \| --- \| --- \| --- \| --- \| --- \| --- \| --- \| --- \| --- \| --- \| --- \| --- \| --- \| --- \| --- \| --- \| --- \| --- \| --- \| --- \| --- \| --- \| \|  \|  \| |
| **Figure 6G. STAT3 level in AAV-Con or AAV-DYRK1A-treated 5xFAD mice** |
| \| Table Analyzed \| pSTAT3 \| \| --- \| --- \| \|  \|  \| \| Column D \| DYRK1A KD \| \| vs. \| vs. \| \| Column C \| CON \| \|  \|  \| \| Unpaired t test \|  \| \| P value \| 0.2919 \| \| P value summary \| ns \| \| Significantly different (P < 0.05)? \| No \| \| One- or two-tailed P value? \| Two-tailed \| \| t, df \| t=1.102, df=12 \| |
| **Figure 6H. p-NF-κB level in AAV-Con or AAV-DYRK1A-treated 5xFAD mice** |
| \| Table Analyzed \| pNFkB \| \| --- \| --- \| \|  \|  \| \| Column B \| DYRK1A KD \| \| vs. \| vs. \| \| Column A \| Con \| \|  \|  \| \| Unpaired t test \|  \| \| P value \| 0.0417 \| \| P value summary \| * \| \| Significantly different (P < 0.05)? \| Yes \| \| One- or two-tailed P value? \| Two-tailed \| \| t, df \| t=2.279, df=12 \| |
| **Figure 6H. NF-κB level in AAV-Con or AAV-DYRK1A-treated 5xFAD mice** |
| \| Table Analyzed \| NFkB \| \| --- \| --- \| \|  \|  \| \| Column D \| DYRK1A KD \| \| vs. \| vs. \| \| Column C \| CON \| \|  \|  \| \| Unpaired t test \|  \| \| P value \| 0.2218 \| \| P value summary \| ns \| \| Significantly different (P < 0.05)? \| No \| \| One- or two-tailed P value? \| Two-tailed \| \| t, df \| t=1.289, df=12 \| |
| **Figure 7A-B. Aβ plaque number in hippocampal CA1 of AAV-shCon or AAV-shDYRK1A-treated 5xFAD mice** |
| \| Table Analyzed \| CA1+DG \| \| --- \| --- \| \|  \|  \| \| Column B \| DYRK1A KD \| \| vs. \| vs. \| \| Column A \| Con \| \|  \|  \| \| Unpaired t test \|  \| \| P value \| 0.0134 \| \| P value summary \| * \| \| Significantly different (P < 0.05)? \| Yes \| \| One- or two-tailed P value? \| Two-tailed \| \| t, df \| t=2.558, df=53 \| |
| **Figure 7A-B. Aβ plaque number in hippocampal DG of AAV-shCon or AAV-shDYRK1A-treated 5xFAD mice** |
| \| Table Analyzed \| CA1+DG \| \| --- \| --- \| \|  \|  \| \| Column D \| DYRK1A KD \| \| vs. \| vs. \| \| Column C \| CON \| \|  \|  \| \| Unpaired t test \|  \| \| P value \| 0.0001 \| \| P value summary \| *** \| \| Significantly different (P < 0.05)? \| Yes \| \| One- or two-tailed P value? \| Two-tailed \| \| t, df \| t=4.134, df=53 \| |
| **Figure 7C. Soluble Aβ40 level in AAV-shCon or AAV-shDYRK1A-treated 5xFAD mice** |
| \| Table Analyzed \| Ab40 \| \| --- \| --- \| \|  \|  \| \| Column B \| DYRK1A KD \| \| vs. \| vs. \| \| Column A \| Con \| \|  \|  \| \| Unpaired t test \|  \| \| P value \| 0.0158 \| \| P value summary \| * \| \| Significantly different (P < 0.05)? \| Yes \| \| One- or two-tailed P value? \| Two-tailed \| \| t, df \| t=2.563, df=29 \| |
| **Figure 7D. Soluble Aβ40 level in AAV-shCon or AAV-shDYRK1A-treated 5xFAD mice** |
| \| Table Analyzed \| Soluble \| \| --- \| --- \| \|  \|  \| \| Column B \| DYRK1A KD \| \| vs. \| vs. \| \| Column A \| Con \| \|  \|  \| \| Unpaired t test \|  \| \| P value \| 0.0004 \| \| P value summary \| *** \| \| Significantly different (P < 0.05)? \| Yes \| \| One- or two-tailed P value? \| Two-tailed \| \| t, df \| t=4.768, df=13 \| |
| **Figure 7D. Soluble Aβ42 level in AAV-shCon or AAV-shDYRK1A-treated 5xFAD mice** |
| \| Table Analyzed \| Soluble \| \| --- \| --- \| \|  \|  \| \| Column D \| DYRK1A KD \| \| vs. \| vs. \| \| Column C \| CON \| \|  \|  \| \| Unpaired t test \|  \| \| P value \| <0.0001 \| \| P value summary \| **** \| \| Significantly different (P < 0.05)? \| Yes \| \| One- or two-tailed P value? \| Two-tailed \| \| t, df \| t=26.40, df=13 \| |
| **Figure 7E. Insoluble Aβ40 level in AAV-shCon or AAV-shDYRK1A-treated 5xFAD mice** |
| \| Table Analyzed \| Insoluble \| \| --- \| --- \| \|  \|  \| \| Column B \| DYRK1A KD \| \| vs. \| vs. \| \| Column A \| Con \| \|  \|  \| \| Unpaired t test \|  \| \| P value \| 0.1683 \| \| P value summary \| ns \| \| Significantly different (P < 0.05)? \| No \| \| One- or two-tailed P value? \| Two-tailed \| \| t, df \| t=1.459, df=13 \| |
| **Figure 7E. Insoluble Aβ42 level in AAV-shCon or AAV-shDYRK1A-treated 5xFAD mice** |
| \| Table Analyzed \| Insoluble \| \| --- \| --- \| \|  \|  \| \| Column D \| DYRK1A KD \| \| vs. \| vs. \| \| Column C \| CON \| \|  \|  \| \| Unpaired t test \|  \| \| P value \| 0.0196 \| \| P value summary \| * \| \| Significantly different (P < 0.05)? \| Yes \| \| One- or two-tailed P value? \| Two-tailed \| \| t, df \| t=2.661, df=13 \| |
| **Figure 7F. DYRK1A level in AAV-shCon or AAV-shDYRK1A-treated 5xFAD mice** |
| \| Table Analyzed \| DYRK1A WB \| \| --- \| --- \| \|  \|  \| \| Column B \| DYRK1A KD \| \| vs. \| vs. \| \| Column A \| Con \| \|  \|  \| \| Unpaired t test \|  \| \| P value \| 0.0145 \| \| P value summary \| * \| \| Significantly different (P < 0.05)? \| Yes \| \| One- or two-tailed P value? \| Two-tailed \| \| t, df \| t=2.856, df=12 \| |
| **Figure 7G. DYRK1A level in AAV-shCon or AAV-shDYRK1A-treated 5xFAD mice** |
| \| Table Analyzed \| DYRK1A \| \| --- \| --- \| \|  \|  \| \| Column B \| DYRK1A KD \| \| vs. \| vs. \| \| Column A \| Con \| \|  \|  \| \| Unpaired t test \|  \| \| P value \| 0.0306 \| \| P value summary \| * \| \| Significantly different (P < 0.05)? \| Yes \| \| One- or two-tailed P value? \| Two-tailed \| \| t, df \| t=2.450, df=12 \| |
| **Figure 7H. BACE-1 activity in AAV-shCon or AAV-shDYRK1A-treated 5xFAD mice** |
| \| Table Analyzed \| BACE-1 \| \| --- \| --- \| \|  \|  \| \| Column B \| DYRK1A KD \| \| vs. \| vs. \| \| Column A \| Con \| \|  \|  \| \| Unpaired t test \|  \| \| P value \| 0.0272 \| \| P value summary \| * \| \| Significantly different (P < 0.05)? \| Yes \| \| One- or two-tailed P value? \| Two-tailed \| \| t, df \| t=2.488, df=13 \| |
| **Figure 7I. ADAM17 activity in AAV-shCon or AAV-shDYRK1A-treated 5xFAD mice** |
| \| Table Analyzed \| ADAM17 \| \| --- \| --- \| \|  \|  \| \| Column B \| DYRK1A KD \| \| vs. \| vs. \| \| Column A \| Con \| \|  \|  \| \| Unpaired t test \|  \| \| P value \| 0.1463 \| \| P value summary \| ns \| \| Significantly different (P < 0.05)? \| No \| \| One- or two-tailed P value? \| Two-tailed \| \| t, df \| t=1.545, df=13 \| |
| **Figure 7J. BACE-1 activity in AAV-shCon or AAV-shDYRK1A-treated 5xFAD mice** |
| \| Table Analyzed \| BACE-1 \| \| --- \| --- \| \|  \|  \| \| Column B \| DYRK1A KD \| \| vs. \| vs. \| \| Column A \| Con \| \|  \|  \| \| Unpaired t test \|  \| \| P value \| 0.0408 \| \| P value summary \| * \| \| Significantly different (P < 0.05)? \| Yes \| \| One- or two-tailed P value? \| Two-tailed \| \| t, df \| t=2.291, df=12 \| |
| **Figure 7K. ADAM17 activity in AAV-shCon or AAV-shDYRK1A-treated 5xFAD mice** |
| \| Table Analyzed \| ADAM17 \| \| --- \| --- \| \|  \|  \| \| Column B \| DYRK1A KD \| \| vs. \| vs. \| \| Column A \| Con \| \|  \|  \| \| Unpaired t test \|  \| \| P value \| 0.2385 \| \| P value summary \| ns \| \| Significantly different (P < 0.05)? \| No \| \| One- or two-tailed P value? \| Two-tailed \| \| t, df \| t=1.240, df=12 \| |
| **Figure 8A. DYRK1A level in AAV-shCon or AAV-shDYRK1A-treated 5xFAD mice** |
| \| Table Analyzed \| DYRK1A WB \| \| --- \| --- \| \|  \|  \| \| Column B \| DYRK1AKD \| \| vs. \| vs. \| \| Column A \| Con \| \|  \|  \| \| Unpaired t test \|  \| \| P value \| <0.0001 \| \| P value summary \| **** \| \| Significantly different (P < 0.05)? \| Yes \| \| One- or two-tailed P value? \| Two-tailed \| \| t, df \| t=6.704, df=14 \| |
| **Figure 8B. Soluble p-Tau^S202/T205^ level in AAV-shCon or AAV-shDYRK1A-treated 5xFAD mice** |
| \| Table Analyzed \| AT8 \| \| --- \| --- \| \|  \|  \| \| Column B \| DYRK1A KD \| \| vs. \| vs. \| \| Column A \| Con \| \|  \|  \| \| Unpaired t test \|  \| \| P value \| 0.2631 \| \| P value summary \| ns \| \| Significantly different (P < 0.05)? \| No \| \| One- or two-tailed P value? \| Two-tailed \| \| t, df \| t=1.166, df=14 \| |
| **Figure 8B. Insoluble p-Tau^S202/T205^ level in AAV-shCon or AAV-shDYRK1A-treated 5xFAD mice** |
| \| Table Analyzed \| AT8 \| \| --- \| --- \| \|  \|  \| \| Column D \| DYRK1A KD \| \| vs. \| vs. \| \| Column C \| CON \| \|  \|  \| \| Unpaired t test \|  \| \| P value \| 0.5903 \| \| P value summary \| ns \| \| Significantly different (P < 0.05)? \| No \| \| One- or two-tailed P value? \| Two-tailed \| \| t, df \| t=0.5510, df=14 \| |
| **Figure 8C. Soluble p-Tau^T212/S214^ level in AAV-shCon or AAV-shDYRK1A-treated 5xFAD mice** |
| \| Table Analyzed \| AT100 \| \| --- \| --- \| \|  \|  \| \| Column B \| DYRK1A KD \| \| vs. \| vs. \| \| Column A \| Con \| \|  \|  \| \| Unpaired t test \|  \| \| P value \| 0.3951 \| \| P value summary \| ns \| \| Significantly different (P < 0.05)? \| No \| \| One- or two-tailed P value? \| Two-tailed \| \| t, df \| t=0.8773, df=14 \| |
| **Figure 8C. Insoluble p-Tau^T212/S214^ level in AAV-shCon or AAV-shDYRK1A-treated 5xFAD mice** |
| \| \| Table Analyzed \| AT100 \| \| --- \| --- \| \|  \|  \| \| Column D \| DYRK1A KD \| \| vs. \| vs. \| \| Column C \| CON \| \|  \|  \| \| Unpaired t test \|  \| \| P value \| 0.3974 \| \| P value summary \| ns \| \| Significantly different (P < 0.05)? \| No \| \| One- or two-tailed P value? \| Two-tailed \| \| t, df \| t=0.8730, df=14 \| \|  \| \| --- \| --- \| --- \| --- \| --- \| --- \| --- \| --- \| --- \| --- \| --- \| --- \| --- \| --- \| --- \| --- \| --- \| --- \| --- \| --- \| --- \| --- \| --- \| --- \| --- \| --- \| \|  \|  \| |
| **Figure 8D. Soluble p-Tau^T231^ level in AAV-shCon or AAV-shDYRK1A-treated 5xFAD mice** |
| \| Table Analyzed \| AT180 \| \| --- \| --- \| \|  \|  \| \| Column B \| DYRK1A KD \| \| vs. \| vs. \| \| Column A \| Con \| \|  \|  \| \| Unpaired t test \|  \| \| P value \| 0.2477 \| \| P value summary \| ns \| \| Significantly different (P < 0.05)? \| No \| \| One- or two-tailed P value? \| Two-tailed \| \| t, df \| t=1.206, df=14 \| |
| **Figure 8D. Insoluble p-Tau^T231^ level in AAV-shCon or AAV-shDYRK1A-treated 5xFAD mice** |
| \| Table Analyzed \| AT180 \| \| --- \| --- \| \|  \|  \| \| Column D \| DYRK1A KD \| \| vs. \| vs. \| \| Column C \| CON \| \|  \|  \| \| Unpaired t test \|  \| \| P value \| 0.2012 \| \| P value summary \| ns \| \| Significantly different (P < 0.05)? \| No \| \| One- or two-tailed P value? \| Two-tailed \| \| t, df \| t=1.341, df=14 \| |
| **Figure 8E. Soluble p-Tau^S396^ level in AAV-shCon or AAV-shDYRK1A-treated 5xFAD mice** |
| \| Table Analyzed \| Ser396 \| \| --- \| --- \| \|  \|  \| \| Column B \| DYRK1A KD \| \| vs. \| vs. \| \| Column A \| Con \| \|  \|  \| \| Unpaired t test \|  \| \| P value \| 0.2861 \| \| P value summary \| ns \| \| Significantly different (P < 0.05)? \| No \| \| One- or two-tailed P value? \| Two-tailed \| \| t, df \| t=1.109, df=14 \| |
| **Figure 8E. Insoluble p-Tau^S396^ level in AAV-shCon or AAV-shDYRK1A-treated 5xFAD mice** |
| \| Table Analyzed \| Ser396 \| \| --- \| --- \| \|  \|  \| \| Column D \| DYRK1A KD \| \| vs. \| vs. \| \| Column C \| CON \| \|  \|  \| \| Unpaired t test \|  \| \| P value \| 0.0082 \| \| P value summary \| ** \| \| Significantly different (P < 0.05)? \| Yes \| \| One- or two-tailed P value? \| Two-tailed \| \| t, df \| t=3.076, df=14 \| |
| **Figure 8F. Soluble p-Tau^S404^ level in AAV-shCon or AAV-shDYRK1A-treated 5xFAD mice** |
| \| Table Analyzed \| Ser404 \| \| --- \| --- \| \|  \|  \| \| Column B \| DYRK1A KD \| \| vs. \| vs. \| \| Column A \| Con \| \|  \|  \| \| Unpaired t test \|  \| \| P value \| 0.3626 \| \| P value summary \| ns \| \| Significantly different (P < 0.05)? \| No \| \| One- or two-tailed P value? \| Two-tailed \| \| t, df \| t=0.9412, df=14 \| |
| **Figure 8F. Insoluble p-Tau^S404^ level in AAV-shCon or AAV-shDYRK1A-treated 5xFAD mice** |
| \| Table Analyzed \| Ser404 \| \| --- \| --- \| \|  \|  \| \| Column D \| DYRK1A KD \| \| vs. \| vs. \| \| Column C \| CON \| \|  \|  \| \| Unpaired t test \|  \| \| P value \| 0.0313 \| \| P value summary \| * \| \| Significantly different (P < 0.05)? \| Yes \| \| One- or two-tailed P value? \| Two-tailed \| \| t, df \| t=2.393, df=14 \| |
| **Figure 8G. p-CDK5 level in AAV-shCon or AAV-shDYRK1A-treated 5xFAD mice** |
| \| Table Analyzed \| pCDK5 \| \| --- \| --- \| \|  \|  \| \| Column B \| DYRK1A KD \| \| vs. \| vs. \| \| Column A \| Con \| \|  \|  \| \| Unpaired t test \|  \| \| P value \| 0.9274 \| \| P value summary \| ns \| \| Significantly different (P < 0.05)? \| No \| \| One- or two-tailed P value? \| Two-tailed \| \| t, df \| t=0.09278, df=14 \| |
| **Figure 8H. p-GSK3α/β level in AAV-shCon or AAV-shDYRK1A-treated 5xFAD mice** |
| \| Table Analyzed \| pGSK3b \| \| --- \| --- \| \|  \|  \| \| Column B \| DYRK1A KD \| \| vs. \| vs. \| \| Column A \| Con \| \|  \|  \| \| Unpaired t test \|  \| \| P value \| 0.0827 \| \| P value summary \| ns \| \| Significantly different (P < 0.05)? \| No \| \| One- or two-tailed P value? \| Two-tailed \| \| t, df \| t=1.869, df=14 \| |
| **Figure 9A. IL-1β mRNA level in AAV-shCon or AAV-shDYRK1A-treated PS19 mice** |
| \| Table Analyzed \| Data 1 \| \| --- \| --- \| \|  \|  \| \| Column B \| Data Set-B \| \| vs. \| vs. \| \| Column A \| Data Set-A \| \|  \|  \| \| Unpaired t test \|  \| \| P value \| <0.0001 \| \| P value summary \| **** \| \| Significantly different (P < 0.05)? \| Yes \| \| One- or two-tailed P value? \| Two-tailed \| \| t, df \| t=6.549, df=13 \| |
| **Figure 9A. TNF-α mRNA level in AAV-shCon or AAV-shDYRK1A-treated PS19 mice** |
| \| Table Analyzed \| Data 1 \| \| --- \| --- \| \|  \|  \| \| Column D \| Data Set-D \| \| vs. \| vs. \| \| Column C \| Data Set-C \| \|  \|  \| \| Unpaired t test \|  \| \| P value \| 0.0183 \| \| P value summary \| * \| \| Significantly different (P < 0.05)? \| Yes \| \| One- or two-tailed P value? \| Two-tailed \| \| t, df \| t=2.730, df=12 \| |
| **Figure 9B. COX-2 mRNA level in AAV-shCon or AAV-shDYRK1A-treated PS19 mice** |
| \| Table Analyzed \| Data 1 \| \| --- \| --- \| \|  \|  \| \| Column B \| Data Set-B \| \| vs. \| vs. \| \| Column A \| Data Set-A \| \|  \|  \| \| Unpaired t test \|  \| \| P value \| 0.4306 \| \| P value summary \| ns \| \| Significantly different (P < 0.05)? \| No \| \| One- or two-tailed P value? \| Two-tailed \| \| t, df \| t=0.8157, df=12 \| |
| **Figure 9B. IL-6 mRNA level in AAV-shCon or AAV-shDYRK1A-treated PS19 mice** |
| \| Table Analyzed \| Data 1 \| \| --- \| --- \| \|  \|  \| \| Column D \| Data Set-D \| \| vs. \| vs. \| \| Column C \| Data Set-C \| \|  \|  \| \| Unpaired t test \|  \| \| P value \| 0.1189 \| \| P value summary \| ns \| \| Significantly different (P < 0.05)? \| No \| \| One- or two-tailed P value? \| Two-tailed \| \| t, df \| t=1.679, df=12 \| |
| **Figure 9C. NLRP3 mRNA level in AAV-shCon or AAV-shDYRK1A-treated PS19 mice** |
| \| Table Analyzed \| Data 1 \| \| --- \| --- \| \|  \|  \| \| Column B \| Data Set-B \| \| vs. \| vs. \| \| Column A \| Data Set-A \| \|  \|  \| \| Unpaired t test \|  \| \| P value \| 0.0330 \| \| P value summary \| * \| \| Significantly different (P < 0.05)? \| Yes \| \| One- or two-tailed P value? \| Two-tailed \| \| t, df \| t=2.408, df=12 \| |
| **Figure 9C. SOD2 mRNA level in AAV-shCon or AAV-shDYRK1A-treated PS19 mice** |
| \| Table Analyzed \| Data 1 \| \| --- \| --- \| \|  \|  \| \| Column D \| Data Set-D \| \| vs. \| vs. \| \| Column C \| Data Set-C \| \|  \|  \| \| Unpaired t test \|  \| \| P value \| 0.0032 \| \| P value summary \| ** \| \| Significantly different (P < 0.05)? \| Yes \| \| One- or two-tailed P value? \| Two-tailed \| \| t, df \| t=3.667, df=12 \| |
| **Figure 9C. SOD2 mRNA level in AAV-shCon or AAV-shDYRK1A-treated PS19 mice** |
| \| Table Analyzed \| Data 1 \| \| --- \| --- \| \|  \|  \| \| Column D \| Data Set-D \| \| vs. \| vs. \| \| Column C \| Data Set-C \| \|  \|  \| \| Unpaired t test \|  \| \| P value \| 0.0032 \| \| P value summary \| ** \| \| Significantly different (P < 0.05)? \| Yes \| \| One- or two-tailed P value? \| Two-tailed \| \| t, df \| t=3.667, df=12 \| |
| **Figure 9D. GFAP mRNA level in AAV-shCon or AAV-shDYRK1A-treated PS19 mice** |
| \| Table Analyzed \| Data 1 \| \| --- \| --- \| \|  \|  \| \| Column B \| Data Set-B \| \| vs. \| vs. \| \| Column A \| Data Set-A \| \|  \|  \| \| Unpaired t test \|  \| \| P value \| 0.0104 \| \| P value summary \| * \| \| Significantly different (P < 0.05)? \| Yes \| \| One- or two-tailed P value? \| Two-tailed \| \| t, df \| t=2.990, df=13 \| |
| **Figure 9E. GBP2 mRNA level in AAV-shCon or AAV-shDYRK1A-treated PS19 mice** |
| \| Table Analyzed \| Data 1 \| \| --- \| --- \| \|  \|  \| \| Column B \| Data Set-B \| \| vs. \| vs. \| \| Column A \| Data Set-A \| \|  \|  \| \| Unpaired t test \|  \| \| P value \| 0.0068 \| \| P value summary \| ** \| \| Significantly different (P < 0.05)? \| Yes \| \| One- or two-tailed P value? \| Two-tailed \| \| t, df \| t=3.266, df=12 \| |
| **Figure 9F. DST mRNA level in AAV-shCon or AAV-shDYRK1A-treated PS19 mice** |
| \| Table Analyzed \| Data 1 \| \| --- \| --- \| \|  \|  \| \| Column B \| Data Set-B \| \| vs. \| vs. \| \| Column A \| Data Set-A \| \|  \|  \| \| Unpaired t test \|  \| \| P value \| 0.9557 \| \| P value summary \| ns \| \| Significantly different (P < 0.05)? \| No \| \| One- or two-tailed P value? \| Two-tailed \| \| t, df \| t=0.05662, df=13 \| |
| **Figure 9F. NESTIN mRNA level in AAV-shCon or AAV-shDYRK1A-treated PS19 mice** |
| \| Table Analyzed \| Data 1 \| \| --- \| --- \| \|  \|  \| \| Column D \| Data Set-D \| \| vs. \| vs. \| \| Column C \| Data Set-C \| \|  \|  \| \| Unpaired t test \|  \| \| P value \| 0.0465 \| \| P value summary \| * \| \| Significantly different (P < 0.05)? \| Yes \| \| One- or two-tailed P value? \| Two-tailed \| \| t, df \| t=2.200, df=13 \| |
| **Figure 9G. CXCL10 mRNA level in AAV-shCon or AAV-shDYRK1A-treated PS19 mice** |
| \| Table Analyzed \| Data 1 \| \| --- \| --- \| \|  \|  \| \| Column B \| Data Set-B \| \| vs. \| vs. \| \| Column A \| Data Set-A \| \|  \|  \| \| Unpaired t test \|  \| \| P value \| 0.0213 \| \| P value summary \| * \| \| Significantly different (P < 0.05)? \| Yes \| \| One- or two-tailed P value? \| Two-tailed \| \| t, df \| t=2.618, df=13 \| |
| **Figure 9H. IBA-1 mRNA level in AAV-shCon or AAV-shDYRK1A-treated PS19 mice** |
| \| Table Analyzed \| Data 1 \| \| --- \| --- \| \|  \|  \| \| Column B \| Data Set-B \| \| vs. \| vs. \| \| Column A \| Data Set-A \| \|  \|  \| \| Unpaired t test \|  \| \| P value \| 0.0188 \| \| P value summary \| * \| \| Significantly different (P < 0.05)? \| Yes \| \| One- or two-tailed P value? \| Two-tailed \| \| t, df \| t=2.684, df=13 \| |
| **Figure 9I. ITGAX mRNA level in AAV-shCon or AAV-shDYRK1A-treated PS19 mice** |
| \| Table Analyzed \| Data 1 \| \| --- \| --- \| \|  \|  \| \| Column B \| Data Set-B \| \| vs. \| vs. \| \| Column A \| Data Set-A \| \|  \|  \| \| Unpaired t test \|  \| \| P value \| 0.0343 \| \| P value summary \| * \| \| Significantly different (P < 0.05)? \| Yes \| \| One- or two-tailed P value? \| Two-tailed \| \| t, df \| t=2.387, df=12 \| |
| **Figure 9I. TREM2 mRNA level in AAV-shCon or AAV-shDYRK1A-treated PS19 mice** |
| \| Table Analyzed \| Data 1 \| \| --- \| --- \| \|  \|  \| \| Column D \| Data Set-D \| \| vs. \| vs. \| \| Column C \| Data Set-C \| \|  \|  \| \| Unpaired t test \|  \| \| P value \| 0.0203 \| \| P value summary \| * \| \| Significantly different (P < 0.05)? \| Yes \| \| One- or two-tailed P value? \| Two-tailed \| \| t, df \| t=2.643, df=13 \| |
| **Figure 9I. CLEC7A mRNA level in AAV-shCon or AAV-shDYRK1A-treated PS19 mice** |
| \| Table Analyzed \| Data 1 \| \| --- \| --- \| \|  \|  \| \| Column F \| Data Set-F \| \| vs. \| vs. \| \| Column E \| Data Set-E \| \|  \|  \| \| Unpaired t test \|  \| \| P value \| 0.0320 \| \| P value summary \| * \| \| Significantly different (P < 0.05)? \| Yes \| \| One- or two-tailed P value? \| Two-tailed \| \| t, df \| t=2.402, df=13 \| |
| **Figure 9J. CR3 mRNA level in AAV-shCon or AAV-shDYRK1A-treated PS19 mice** |
| \| Table Analyzed \| Data 1 \| \| --- \| --- \| \|  \|  \| \| Column B \| Data Set-B \| \| vs. \| vs. \| \| Column A \| Data Set-A \| \|  \|  \| \| Unpaired t test \|  \| \| P value \| 0.0040 \| \| P value summary \| ** \| \| Significantly different (P < 0.05)? \| Yes \| \| One- or two-tailed P value? \| Two-tailed \| \| t, df \| t=3.491, df=13 \| |
| **Figure 9J. C1QA mRNA level in AAV-shCon or AAV-shDYRK1A-treated PS19 mice** |
| \| Table Analyzed \| Data 1 \| \| --- \| --- \| \|  \|  \| \| Column D \| Data Set-D \| \| vs. \| vs. \| \| Column C \| Data Set-C \| \|  \|  \| \| Unpaired t test \|  \| \| P value \| 0.0428 \| \| P value summary \| * \| \| Significantly different (P < 0.05)? \| Yes \| \| One- or two-tailed P value? \| Two-tailed \| \| t, df \| t=2.245, df=13 \| |
| **Supplementary Figure 1A. p-CaMKIIα level in AAV-Con or AAV-DYRK1A-treated WT mice** |
| \| Table Analyzed \| p-CaMKIIa \| \| --- \| --- \| \|  \|  \| \| Column B \| Data Set-B \| \| vs. \| vs. \| \| Column A \| Data Set-A \| \|  \|  \| \| Unpaired t test \|  \| \| P value \| 0.4288 \| \| P value summary \| ns \| \| Significantly different (P < 0.05)? \| No \| \| One- or two-tailed P value? \| One-tailed \| \| t, df \| t=0.1826, df=14 \| |
| **Supplementary Figure 1A. CaMKIIa level in AAV-Con or AAV-DYRK1A-treated WT mice** |
| \| Table Analyzed \| CaMKIIa \| \| --- \| --- \| \|  \|  \| \| Column D \| Data Set-D \| \| vs. \| vs. \| \| Column C \| Data Set-C \| \|  \|  \| \| Unpaired t test \|  \| \| P value \| 0.1669 \| \| P value summary \| ns \| \| Significantly different (P < 0.05)? \| No \| \| One- or two-tailed P value? \| Two-tailed \| \| t, df \| t=1.458, df=14 \| |
| **Supplementary Figure 1B. p-CREB level in AAV-Con or AAV-DYRK1A-treated WT mice** |
| \| Table Analyzed \| p-CREB \| \| --- \| --- \| \|  \|  \| \| Column B \| Data Set-B \| \| vs. \| vs. \| \| Column A \| Data Set-A \| \|  \|  \| \| Unpaired t test \|  \| \| P value \| 0.3864 \| \| P value summary \| ns \| \| Significantly different (P < 0.05)? \| No \| \| One- or two-tailed P value? \| One-tailed \| \| t, df \| t=0.2944, df=14 \| |
| **Supplementary Figure 1B. CREB level in AAV-Con or AAV-DYRK1A-treated WT mice** |
| \| Table Analyzed \| CREB \| \| --- \| --- \| \|  \|  \| \| Column D \| Data Set-D \| \| vs. \| vs. \| \| Column C \| Data Set-C \| \|  \|  \| \| Unpaired t test \|  \| \| P value \| 0.0557 \| \| P value summary \| ns \| \| Significantly different (P < 0.05)? \| No \| \| One- or two-tailed P value? \| Two-tailed \| \| t, df \| t=2.086, df=14 \| |
| **Supplementary Figure 1C. p-ERK level in AAV-Con or AAV-DYRK1A-treated WT mice** |
| \| Table Analyzed \| p-ERK \| \| --- \| --- \| \|  \|  \| \| Column B \| Data Set-B \| \| vs. \| vs. \| \| Column A \| Data Set-A \| \|  \|  \| \| Unpaired t test with Welch's correction \|  \| \| P value \| 0.0638 \| \| P value summary \| ns \| \| Significantly different (P < 0.05)? \| No \| \| One- or two-tailed P value? \| Two-tailed \| \| Welch-corrected  t, df \| t=2.198, df=7.023 \| |
| **Supplementary Figure 1C. ERK level in AAV-Con or AAV-DYRK1A-treated WT mice** |
| \| Table Analyzed \| ERK \| \| --- \| --- \| \|  \|  \| \| Column D \| Data Set-D \| \| vs. \| vs. \| \| Column C \| Data Set-C \| \|  \|  \| \| Unpaired t test \|  \| \| P value \| 0.8317 \| \| P value summary \| ns \| \| Significantly different (P < 0.05)? \| No \| \| One- or two-tailed P value? \| Two-tailed \| \| t, df \| t=0.2165, df=14 \| |
| **Supplementary Figure 1D. PLK2 level in AAV-Con or AAV-DYRK1A-treated WT mice** |
| \| Table Analyzed \| PLK2 \| \| --- \| --- \| \|  \|  \| \| Column B \| Data Set-B \| \| vs. \| vs. \| \| Column A \| Data Set-A \| \|  \|  \| \| Unpaired t test \|  \| \| P value \| 0.2433 \| \| P value summary \| ns \| \| Significantly different (P < 0.05)? \| No \| \| One- or two-tailed P value? \| One-tailed \| \| t, df \| t=0.7146, df=14 \| |
| **Supplementary Figure 2A, B. Y-maze, Spontaneous alternation in WT and 5xFAD mice** |
| \| Table Analyzed \| Figure 1E \| \| --- \| --- \| \|  \|  \| \| Column B \| 5xFAD \| \| vs. \| vs. \| \| Column A \| Con \| \|  \|  \| \| Unpaired t test \|  \| \| P value \| 0.0315 \| \| P value summary \| * \| \| Significantly different (P < 0.05)? \| Yes \| \| One- or two-tailed P value? \| Two-tailed \| \| t, df \| t=2.305, df=21 \| |
| **Supplementary Figure 2A, B. Y- maze, Total arm entry in WT and 5xFAD mice** |
| \| Table Analyzed \| Figure 1E \| \| --- \| --- \| \|  \|  \| \| Column D \| 5xFAD \| \| vs. \| vs. \| \| Column C \| Con \| \|  \|  \| \| Mann Whitney test \|  \| \| P value \| 0.0657 \| \| Exact or approximate P value? \| Exact \| \| P value summary \| ns \| \| Significantly different (P < 0.05)? \| No \| \| One- or two-tailed P value? \| Two-tailed \| |
| **Supplementary Figure 2C, D. NOR Training in WT and 5xFAD mice** |
| \| Table Analyzed \| Figure 1F \| \| --- \| --- \| \|  \|  \| \| Column B \| 5xFAD \| \| vs. \| vs. \| \| Column A \| Con \| \|  \|  \| \| Unpaired t test \|  \| \| P value \| 0.3184 \| \| P value summary \| ns \| \| Significantly different (P < 0.05)? \| No \| \| One- or two-tailed P value? \| Two-tailed \| \| t, df \| t=1.019, df=24 \| |
| **Supplementary Figure 2C, D. NOR Test in WT and 5xFAD mice** |
| \| Table Analyzed \| Figure 1F \| \| --- \| --- \| \|  \|  \| \| Column D \| 5xFAD \| \| vs. \| vs. \| \| Column C \| Con \| \|  \|  \| \| Unpaired t test \|  \| \| P value \| 0.0346 \| \| P value summary \| * \| \| Significantly different (P < 0.05)? \| Yes \| \| One- or two-tailed P value? \| Two-tailed \| \| t, df \| t=2.240, df=24 \| |
| **Supplementary Figure 3A. NR2A level in AAV-shCon or AAV-shDYRK1A-treated 5xFAD mice** |
| \| Table Analyzed \| NR2A \| \| --- \| --- \| \|  \|  \| \| Column B \| DYRK1A KD \| \| vs. \| vs. \| \| Column A \| Con \| \|  \|  \| \| Unpaired t test \|  \| \| P value \| 0.3888 \| \| P value summary \| ns \| \| Significantly different (P < 0.05)? \| No \| \| One- or two-tailed P value? \| One-tailed \| \| t, df \| t=0.2879, df=14 \| |
| **Supplementary Figure 3B. NR2B level in AAV-shCon or AAV-shDYRK1A-treated 5xFAD mice** |
| \| Table Analyzed \| NR2B \| \| --- \| --- \| \|  \|  \| \| Column B \| DYRK1A KD \| \| vs. \| vs. \| \| Column A \| Con \| \|  \|  \| \| Unpaired t test \|  \| \| P value \| 0.1798 \| \| P value summary \| ns \| \| Significantly different (P < 0.05)? \| No \| \| One- or two-tailed P value? \| One-tailed \| \| t, df \| t=0.9472, df=14 \| |
| **Supplementary Figure 3C. Glu1A level in AAV-shCon or AAV-shDYRK1A-treated 5xFAD mice** |
| \| Table Analyzed \| GluA1 \| \| --- \| --- \| \|  \|  \| \| Column B \| DYRK1A KD \| \| vs. \| vs. \| \| Column A \| Con \| \|  \|  \| \| Unpaired t test \|  \| \| P value \| 0.3426 \| \| P value summary \| ns \| \| Significantly different (P < 0.05)? \| No \| \| One- or two-tailed P value? \| One-tailed \| \| t, df \| t=0.4139, df=14 \| |
| **Supplementary Figure 3C. Glu2A level in AAV-shCon or AAV-shDYRK1A-treated 5xFAD mice** |
| \| Table Analyzed \| GluA2 \| \| --- \| --- \| \|  \|  \| \| Column D \| DYRK1A KD \| \| vs. \| vs. \| \| Column C \| CON \| \|  \|  \| \| Unpaired t test \|  \| \| P value \| 0.1897 \| \| P value summary \| ns \| \| Significantly different (P < 0.05)? \| No \| \| One- or two-tailed P value? \| Two-tailed \| \| t, df \| t=1.378, df=14 \| |
| **Supplementary Figure 3D. EAAT1 level in AAV-shCon or AAV-shDYRK1A-treated 5xFAD mice** |
| \| Table Analyzed \| EAAT1 \| \| --- \| --- \| \|  \|  \| \| Column B \| DYRK1A KD \| \| vs. \| vs. \| \| Column A \| Con \| \|  \|  \| \| Unpaired t test \|  \| \| P value \| 0.2065 \| \| P value summary \| ns \| \| Significantly different (P < 0.05)? \| No \| \| One- or two-tailed P value? \| Two-tailed \| \| t, df \| t=1.335, df=12 \| |
| **Supplementary Figure 3D. EAAT2 level in AAV-shCon or AAV-shDYRK1A-treated 5xFAD mice** |
| \| Table Analyzed \| EAAT2 \| \| --- \| --- \| \|  \|  \| \| Column B \| DYRK1A KD \| \| vs. \| vs. \| \| Column A \| Con \| \|  \|  \| \| Unpaired t test \|  \| \| P value \| 0.5424 \| \| P value summary \| ns \| \| Significantly different (P < 0.05)? \| No \| \| One- or two-tailed P value? \| Two-tailed \| \| t, df \| t=0.6270, df=12 \| |
| **Supplementary Figure 3E. p-ERK level in AAV-shCon or AAV-shDYRK1A-treated 5xFAD mice** |
| \| Table Analyzed \| pERK \| \| --- \| --- \| \|  \|  \| \| Column B \| DYRK1A KD \| \| vs. \| vs. \| \| Column A \| Con \| \|  \|  \| \| Unpaired t test \|  \| \| P value \| 0.3027 \| \| P value summary \| ns \| \| Significantly different (P < 0.05)? \| No \| \| One- or two-tailed P value? \| Two-tailed \| \| t, df \| t=1.070, df=14 \| |
| **Supplementary Figure 3E. ERK level in AAV-shCon or AAV-shDYRK1A-treated 5xFAD mice** |
| \| Table Analyzed \| ERK \| \| --- \| --- \| \|  \|  \| \| Column C \| CON \| \| vs. \| vs. \| \| Column D \| DYRK1A KD \| \|  \|  \| \| Kolmogorov-Smirnov test \|  \| \| P value \| 0.0870 \| \| Exact or approximate P value? \| Exact \| \| P value summary \| ns \| \| Significantly different (P < 0.05)? \| No \| \| Kolmogorov-Smirnov D \| 0.6250 \| |
| **Supplementary Figure 4A. NEP activity in AAV-shCon or AAV-shDYRK1A-treated 5xFAD mice** |
| \| Table Analyzed \| NEP \| \| --- \| --- \| \|  \|  \| \| Column B \| DYRK1A KD \| \| vs. \| vs. \| \| Column A \| Con \| \|  \|  \| \| Unpaired t test \|  \| \| P value \| 0.2493 \| \| P value summary \| ns \| \| Significantly different (P < 0.05)? \| No \| \| One- or two-tailed P value? \| One-tailed \| \| t, df \| t=0.6936, df=15 \| |
| **Supplementary Figure 4B. IDE activity in AAV-shCon or AAV-shDYRK1A-treated 5xFAD mice** |
| \| Table Analyzed \| IDE \| \| --- \| --- \| \|  \|  \| \| Column B \| DYRK1A KD \| \| vs. \| vs. \| \| Column A \| Con \| \|  \|  \| \| Unpaired t test \|  \| \| P value \| 0.2754 \| \| P value summary \| ns \| \| Significantly different (P < 0.05)? \| No \| \| One- or two-tailed P value? \| One-tailed \| \| t, df \| t=0.6114, df=14 \| |
| **Supplementary Figure 4C, D. PS1-CTF level in AAV-shCon or AAV-shDYRK1A-treated 5xFAD mice** |
| \| Table Analyzed \| PS1-CTF \| \| --- \| --- \| \|  \|  \| \| Column B \| DYRK1A KD \| \| vs. \| vs. \| \| Column A \| Con \| \|  \|  \| \| Unpaired t test \|  \| \| P value \| 0.4251 \| \| P value summary \| ns \| \| Significantly different (P < 0.05)? \| No \| \| One- or two-tailed P value? \| One-tailed \| \| t, df \| t=0.1931, df=12 \| |
| **Supplementary Figure 4E, F. p-APP^Thr668^ level in AAV-shCon or AAV-shDYRK1A-treated 5xFAD mice** |
| \| Table Analyzed \| pAPP \| \| --- \| --- \| \|  \|  \| \| Column B \| DYRK1A KD \| \| vs. \| vs. \| \| Column A \| Con \| \|  \|  \| \| Unpaired t test \|  \| \| P value \| 0.0976 \| \| P value summary \| ns \| \| Significantly different (P < 0.05)? \| No \| \| One- or two-tailed P value? \| One-tailed \| \| t, df \| t=1.372, df=12 \| |
| **Supplementary Figure 4G, H. p-APP^Thr668^ level in AAV-shCon or AAV-shDYRK1A-treated 5xFAD mice** |
| \| Table Analyzed \| pAPP \| \| --- \| --- \| \|  \|  \| \| Column B \| DYRK1A KD \| \| vs. \| vs. \| \| Column A \| Con \| \|  \|  \| \| Unpaired t test \|  \| \| P value \| 0.0891 \| \| P value summary \| ns \| \| Significantly different (P < 0.05)? \| No \| \| One- or two-tailed P value? \| One-tailed \| \| t, df \| t=1.430, df=12 \| |
| **Supplementary Figure 5A, B. p-Tau^S202/T205^ level in AAV-shCon or AAV-shDYRK1A-treated 5xFAD mice** |
| \| Table Analyzed \| AT8 \| \| --- \| --- \| \|  \|  \| \| Column B \| DYRK1A KD \| \| vs. \| vs. \| \| Column A \| Con \| \|  \|  \| \| Unpaired t test \|  \| \| P value \| 0.6055 \| \| P value summary \| ns \| \| Significantly different (P < 0.05)? \| No \| \| One- or two-tailed P value? \| Two-tailed \| \| t, df \| t=0.5304, df=12 \| |
| **Supplementary Figure 5C, D. p-Tau^T231^ level in AAV-shCon or AAV-shDYRK1A-treated 5xFAD mice** |
| \| Table Analyzed \| at180 \| \| --- \| --- \| \|  \|  \| \| Column B \| DYRK1A KD \| \| vs. \| vs. \| \| Column A \| Con \| \|  \|  \| \| Unpaired t test \|  \| \| P value \| 0.1108 \| \| P value summary \| ns \| \| Significantly different (P < 0.05)? \| No \| \| One- or two-tailed P value? \| Two-tailed \| \| t, df \| t=1.702, df=14 \| |
